# Boron-mediated one-pot access to salicylaldehydes *via ortho*-C–H hydroxylation of benzaldehydes†

**DOI:** 10.1039/d4ra02994a

**Published:** 2024-06-20

**Authors:** Ruiyang Wang, Xu Feng, Boya Feng, Yu Chen

**Affiliations:** a Jiangsu Key Laboratory for the Research and Utilization of Plant Resources, Jiangsu Province Engineering Research Center of Eco-cultivation and High-value Utilization of Chinese Medicinal Materials, Institute of Botany, Jiangsu Province and Chinese Academy of Sciences (Nanjing Botanical Garden Mem. Sun Yat-Sen) Nanjing 210014 China boyafeng@gmail.com ychen@jib.ac.cn

## Abstract

A novel protocol has been devised for the *ortho*-C–H hydroxylation of benzaldehydes. Directed by a transient imine group, the borylation of benzaldehydes, sequentially followed by the hydroxylation, furnishes diverse salicylaldehydes in a one-pot manner. The resultant salicylaldehydes could be readily applied in the downstream synthesis to produce bioactive molecules.

## Abstract 2

Described herein is a boron-mediated *ortho*-C–H hydroxylation of aromatic aldehydes assisted by a transient imine directing group, providing an efficient method for the synthesis of salicylaldehydes.

## Graphical abstract

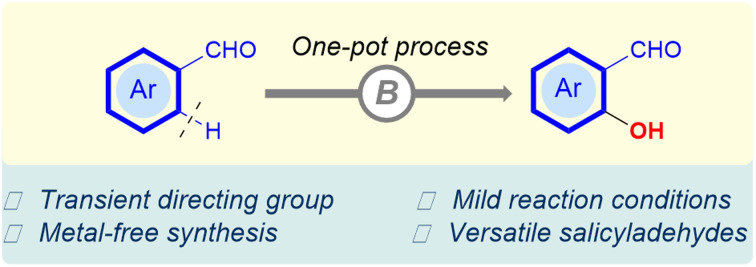

## Introduction

**Scheme 1:**
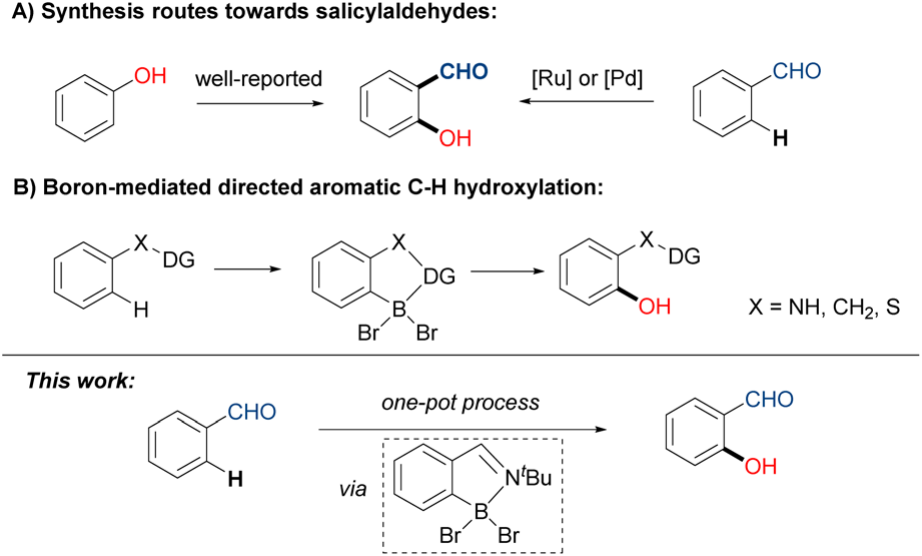
Synthetic strategies of *ortho*-substituted phenols: (A) generation of salicylaldehydes; (B) boron-mediated directed C–H hydroxylation.

Salicylaldehyde and its derivatives represent ubiquitous bioactive motifs found in cosmetics, natural products, and fragrances.^1^ They also function as valuable synthetic building blocks or even photocatalysts in the construction of various complex scaffolds.^2^ The conventional preparation of salicylaldehyde derivatives relies on the formylation of phenols through reactions such as the Reimer–Tiemann reaction, Vilsmeier–Haack reaction, and Duff reaction.^3^ Thanks to the rapid development of C–H activation, diverse opportunities of aromatic C–H hydroxylation have been unlocked, presenting alternative synthetic approaches to produce salicylaldehyde derivatives. Transition-metal catalyzed C–H hydroxylation, assisted by directing groups like pyridine and amide, enable the streamlined synthesis of various *ortho*-substituted phenols.^4^ However, the direct hydroxylation on the adjacent position of aldehyde has been less developed,^5^ likely hampered by the weak coordinating ability and the undesired oxidation tendency of –CHO group. The elegant catalyzed examples including a ruthenium system developed by Ackermann^5*a*^ and a palladium system achieved by Sorensen (Scheme 1A).^5*b*^ Despite their high regioselectivity, these protocols suffered from complicated operations, transition-metal loading, and costly reagents (hypervalent iodine or the F^+^ oxidant).

On the other hand, metal-free C–H borylation offers an alternative for regioselective C–H activation, as pioneering work by Maclean demonstrated.^6^ However, this method requires extremely high temperatures. In 2019, the BBr_3_-mediated borylation of indoles was developed by Shi and Ingleson, independently.^7^ Following the establishment of C–B bonds, Shi and co-workers further expanded their concept, realizing the hydroxylation of anilines and indoles.^8^ Ji's group also reported a boron-mediated hydroxylation with 3,4,5-tribromopyrazole as chelating group.^9^ Due to the nature of electrophilic aromatic substitution (S_E_Ar), the BBr_3_-mediated transformations primarily depend on the electron-rich arenes (Scheme 1B), such as indoles,^7^ anilines,^7*a*,10^ phenols,^11^ thiophenols^12^ and pyrroles.^13^ Thus, the modification of electron-poor aromatic systems like benzaldehydes are challenging. To address the drawback, the adaption of an imine transient directing group (TDG) in boron-mediated borylation of benzaldehydes was elegantly proposed and illustrated by Rej and Chatani.^14^ Very recently, Ji and co-workers also demonstrated a borylation of benzophenones with hydrazone as traceless directing group.^15^ Inspired by Chatani's work, we anticipate the transient directing group strategy will be feasible for forging *ortho*-C–O bonds on ubiquitous benzaldehyde derivatives. We are thereby interested in combining sequential TDG installation/borylation/hydroxylation/TDG removal in one pot, targeting the straightforward synthesis of structurally significant salicylaldehyde motifs.

## Results and discussion

We initiated our investigation with the hydroxylation of 2-methylbenzaldehyde (1a) as the model substrate (Table 1). The reaction was carried out in three steps: (i) 2-methylbenzaldehyde was reacted with excess ^*t*^BuNH_2_ (4.0 eq.) in DCE at 70 °C for 4 h for fully conversion to imine; (ii) the borylation was then carried out by the addition of BBr_3_ (2.0 eq.) and 2,6 lutidine (2.0 eq.) in DCE at room temperature for 12 h; (iii) cosolvent MTBE (methyl *t*-butyl ether) and H_2_O (v/v, 1 : 1) was injected followed by the addition of NaBO_3_·4H_2_O (3.0 eq.) to furnish the hydroxylation and removal of directing group. Pleasingly, the primary reaction conditions resulted in a total yield of 63% (entry 1). Either less amount of ^*t*^BuNH_2_ or elevation of temperature (ii) led to a slightly diminished yield (entries 2 and 3). The overall yield remarkably decreased when the solvent for borylation process (ii) was altered to DCM (entry 4). The effect of base in borylation was also examined as product was hardly formed without base (entry 5). 2,6-Lutidine was recognized as an superior base balancing both borenium species generation and reversible acid/base interaction.^16^ On the contrary, triethylamine, pyridine and 2,3,5,6-tetramethylpyrazine were totally ineffective while the desired product 2a was detected in 31% yield with 2,4,6-trimethylpyridine (entries 6–9). Neat BBr_3_ appeared as same effective as its solution in dichloromethane while BCl_3_ was totally ineffective (entries 10 and 11). We further explored the reaction conditions with BBr_3_ (2*N* in DCM) as its more stable under laboratory-bench storage conditions and readily commercially available. A slight increase in BBr_3_ to 2.5 equivalent promoted the yield to 79% (entry 12). Further adjustments on the stoichiometry showed no benefits (entries 13 and 14). For the oxygenation process (iii), other common oxidants including oxone, hydrogen peroxide and sodium percarbonate failed to give effective yields (entries 15–17). The organic component of cosolvent utilized in step (iii) turned out to be an impressive factor for the transformation. THF was competitive while other ether solvents led to diminished yields (entries 18–20). The desired product 2a was merely obtained by the switch of imine directing group to rigid amantadine (entry 21).

**Table 1:** Optimization of the reaction conditions^a^

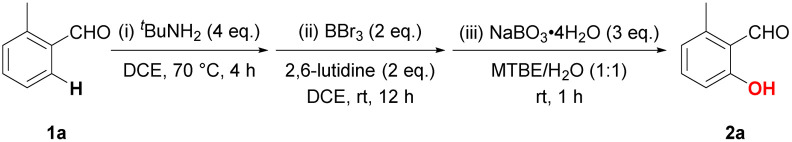		
Entry	Variation from the “primary conditions”	Yield^b^
1	None	63
2	^*t*^BuNH_2_ (2 eq.)	46
3	40 °C(II)	48
4	DCM instead of DCE (II)	25
5	Without 2,6-lutidine	1
6	Triethylamine instead of 2,6-lutidine	0
7	2,4,6-Trimethylpyridine instead of 2,6-lutidine	31
8	Pyridine instead of 2,6-lutidine	0
9	2,3,5,6-Tetramethylpyrazine instead of 2,6-lutidine	1
10	BBr_3_ (neat) instead of BBr_3_ (2*N* in DCM)	63
11	BCl_3_ instead of BBr_3_	0
12	BBr_3_ (2.5 eq., 2*N* in DCM)	79
13	BBr_3_ (3.0 eq., 2*N* in DCM)	74
14	2,6-Lutidine (1.2 eq.), BBr_3_ (1.5 eq., 2*N* in DCM)	23
15	Oxone instead of NaBO_3_·4H_2_O	0
16	H_2_O_2_ instead of NaBO_3_·4H_2_O	2
17	2Na_2_CO_3_·3H_2_O_2_ instead of NaBO_3_·4H_2_O	11
18	1,4-Dioxane instead of MTBE	40
19	THF instead of MTBE	55
20	Ether instead of MTBE	31
21	1-AdNH_2_ instead of ^*t*^BuNH_2_	1

a Primary conditions: (i) 1a (0.4 mmol, 1 eq.) and ^*t*^BuNH_2_ (1.6 mmol, 4 eq.) in 1,2-DCE (1.5 mL) at 70 °C for 4 h under argon, then evaporation under reduced pressure to remove excess ^*t*^BuNH_2_, water and DCE; (ii) BBr_3_ (0.4 mL, 2*N* in DCM, 2.0 eq.) and 2,6-lutidine (0.8 mmol, 2 eq.) in 1,2-DCE (1 mL) at r.t. for 12 h under argon, then evaporation under reduced pressure; (iii) NaBO_3_·4H_2_O (1.2 mmol, 3 eq.) in MTBE/H_2_O (v/v = 1 : 1, 2 mL) at r.t. under argon for 1 h.

b GC yield (internal standard: mesitylene).

Based on the optimal reaction conditions, we subsequently tested the substrate scope with a number of benzaldehyde derivatives (Table 2). Similar to the standard product 2a, the introduction or deletion of a methyl group on the phenyl ring hardly affects the reaction. Treatment of aldehyde 1b and 1c under standard conditions led to good yields of 78% and 89%, respectively. However, diminished yields were detected when a bulkier phenyl substituent was introduced to either *ortho*- or *para*-position, reducing the yields to 63% and 43%, respectively (2d and 2e). The *para*-benzyl substituted benzaldehyde exhibited better reactivity, as product 2f was obtained in 61% yield. To our delight, electron-withdrawn halogen groups (F, Cl and Br) were compatible with our protocol, exemplified by the successful generation of products 2g–2k, which could also serve as useful synthetic handles for the further elaboration of salicylaldehydes. Due to the intrinsic electrophilicity of boron, the *ortho*-C–H hydroxylation preferentially react on the presumably electron-richer position (*e.g.*^1^H NMR chemical for 1k: *δ*_H-6_ = 7.45 ppm; *δ*_H-2_ = 7.51 ppm).^17^ Despite the decomposition of substituents caused by BBr_3_, methoxy and phenyloxy group were also tolerated under the standard conditions with decreased yields (2l and 2m). Moreover, condensed (hetero)aromatic substrates could also undergo the hydroxylation reaction to furnish the desired products in moderate to good yields (2n–2s). Notably, the hydroxylation of 2-naphthaldehyde resulted in a mixture of 1-hydroxylated and 3-hydroxylated products which could be isolated with yields of 42% and 30%, respectively (2o and 2o′). On the other side of the coin, 9*H*-fluorene-2-carbaldehyde and 9-methyl-9*H*-carbazole-2-carbaldehyde delivered respectively sole products 2q and 2r, whereas their selectivity is opposite (determined by the HMBC NMR, Fig. S1 and S2†).

**Table 2:** Substrate scope^a^

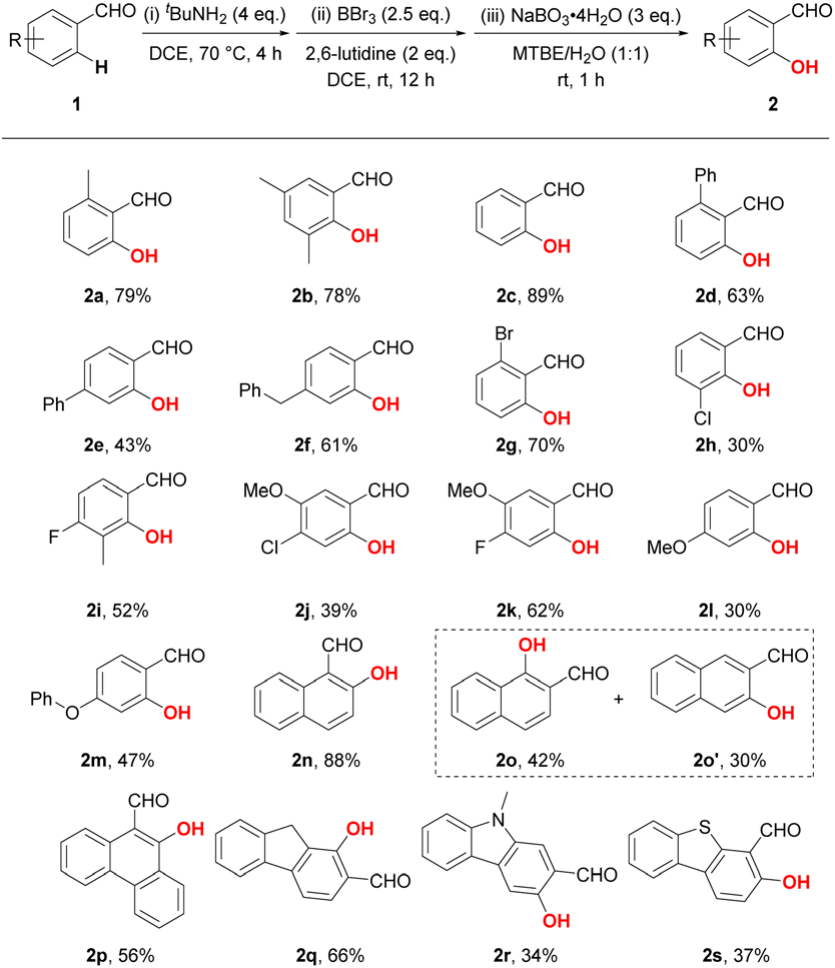

a Reaction conditions: (i) 1 (0.4 mmol, 1 eq.) and ^*t*^BuNH_2_ (1.6 mmol, 4 eq.) in 1,2-DCE (1.5 mL) at 70 °C for 4 h under argon, then evaporation under reduced pressure; (ii) BBr_3_ (0.5 mL, 2*N* in DCM, 2.5 eq.) and 2,6-lutidine (0.8 mmol, 2 eq.) in 1,2-DCE (1 mL) at r.t. for 12 h under argon, then evaporation under reduced pressure; (iii) NaBO_3_·4H_2_O (1.2 mmol, 3 eq.) in MTBE/H_2_O (1 : 1, 2 mL) at r.t. under argon for 1 h.

**Scheme 2:**
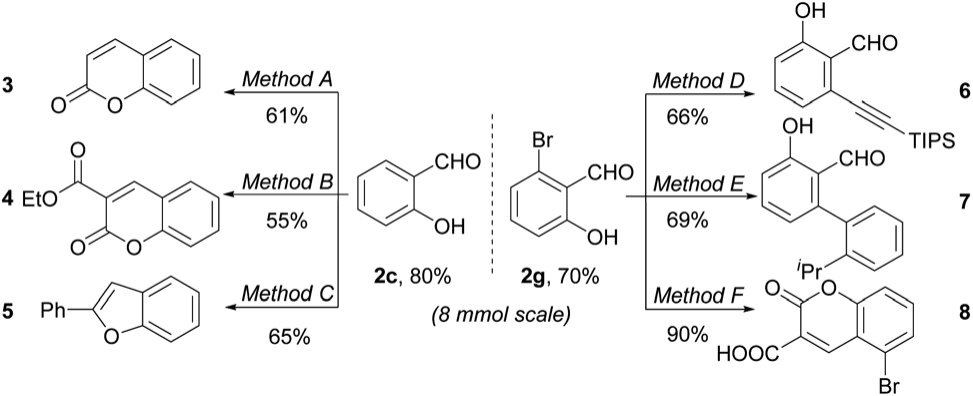
Examples for downstream derivation. Reaction conditions: method A: (1) MeI, NaH, THF, DMF, 0 °C, 2 h, then r.t., 3 h; (2) Ph_3_P=CHCOOEt, DCM, 40 °C, 4 h; (3) BBr_3_, DCM, 50 °C, 4 h. Method B: ethyl cyanoacetate, FeCl_3_, EtOH, 80 °C, 24 h. Method C: (1) Ph_3_P=CBr_2_, DCM, 0 °C, 0.5 h, then r.t., 2 h; (2) PhB(OH)_2_, Pd(OAc)_2_(5 mol%), CuI (5 mol%), PPh_3_, K_3_PO_4_, 1,4-dioxane, 100 °C, 24 h. Method D: Pd(dppf)Cl_2_ (10 mol%), (triisopropylsilyl)acetylene, CuI (20 mol%), Et_3_N, 90 °C, overnight. Method E: 2-isopropylphenylboronic acid, Pd(PPh_3_)_4_ (3 mol%), Na_2_CO_3_, H_2_O/MeOH/DME, 80 °C, overnight. Method F: (1) 2,2-dimethyl-1,3-dioxane-4,6-dione, ammonium acetate, H_2_O, r.t. 3 h; (2) HCl, pH = 4.5.

To further illustrate the utility of our protocol, 8 mmol synthesis of 2c and 2g were conducted. As shown in Scheme 2, the salicylaldehydes 2c and 2g were isolated in yields of 80% and 70%, respectively, with no starting material left, proving the scale-up robustness of the protocol. The downstream derivation of formyl and phenol group has also been exploited. Valuable bioactive heterocycles including substituted coumarins and benzofuran could be easily prepared from the salicylaldehyde motifs.^5*a*,18^ Moreover, the compatible –Br group could be further functionalized, exemplified by alkynylation and arylation. Further applications of the resultant salicylaldehyde in antimicrobials are undergoing in our laboratory.

## Experimental

### General procedure for the *ortho*-C–H hydroxylation

(i) An oven-dried Schlenk tube equipped with a stirring bar was charged with benzaldehyde 1 (0.4 mmol, 1.0 equiv.), ^*t*^BuNH_2_ (168 μL, 4.0 equiv.) under argon. Then solvent DCE (1 mL) was added at room temperature. The reaction mixture was placed in a preheated oil bath at 70 °C, and stirred for 4 h.

(ii) Next, ^*t*^BuNH_2_ and solvent was removed under reduced pressure. 2,6-Lutidine (85.7 mg, 2.0 equiv.), DCE (1 mL) and BBr_3_ (0.5 mL, 2*N* in DCM, 2.5 equiv.) were added under argon. The reaction mixture was stirred at r.t. for 12 h.

(iii) Then, solvent was removed under reduced pressure, followed by the addition of MTBE (1 mL), sodium perborate tetrahydrate (185 mg, 1.2 mmol, 3.0 equiv.) and water (1 mL) under argon. The reaction mixture was stirred at r.t. for another 1 h. Afterwards, the reaction mixture was extracted with ethyl acetate (10 mL × 3). The combined organic layer was further purified by column chromatography on silica gel (200–300 mesh) to afford the corresponding product 2.

## Conclusions

In conclusion, we have developed a novel one-pot procedure for the rapid synthesis of salicylaldehydes. The BBr_3_-mediated *ortho*-C–H hydroxylation assisted by a transient imine directing group features high regioselectivity, mild reaction conditions and good compatibility. By patiently tuning the reaction conditions, TDG installation/borylation/hydroxylation/TDG removal, are combined in one pot, omitting isolation of intermediates. The strategy rapidly assembles structurally diverse salicylaldehydes, which are capable to serve as key intermediates in the synthetic and medicinal chemistry.

## Data availability

A data availability statement (DAS) is required to be submitted alongside all articles. Please read our full guidance on data availability statements (https://www.rsc.org/journals-books-databases/author-and-reviewer-hub/authors-information/prepare-and-format/data-sharing/) for more details and examples of suitable statements you can use.

## Author contributions

BF conceived the project; RW and BF performed the research; XF contributed to the chemical characterization/data analysis; RW and BF wrote the final manuscript; BF and YC revised the manuscript and supervised the project.

## Conflicts of interest

There are no conflicts to declare.

## Supplementary Material

RA-014-D4RA02994A-s001
