# Supplementary material for: Boron-mediated one-pot access to salicylaldehydes via ortho-C–H hydroxylation of benzaldehydes
Source: RSC Adv. 2024 Jun 20;14(28):19922–5. doi: 10.1039/d4ra02994a (PMC11187805; doi:10.1039/d4ra02994a)

Electronic Supplementary Material (ESI) for RSC Advances.
This journal is © The Royal Society of Chemistry 2024

Supporting Information

Boron-mediated One-pot Access to Salicylaldehydes via *ortho*-C–H hydroxylation of benzaldehydes

Ruiyang Wang, Xu Feng, Boya Feng and Yu Chen**

Jiangsu Key Laboratory for the Research and Utilization of Plant Resources,

Jiangsu Province Engineering Research Center of Eco-cultivation and High-value Utilization of Chinese Medicinal Materials,

Institute of Botany, Jiangsu Province and Chinese Academy of Sciences (Nanjing Botanical Garden Mem. Sun Yat-Sen),

Nanjing, 210014, China

E-mail: boyafeng@gmail.com; ychen@jib.ac.cn

Contents

I. General remarks S1

II. General procedure for the optimization study S1

III. Experimental data for the described substances S2

IV. Unsuccessful substrates S13

V. Scale-up reaction and downstream synthesis S13

VI. References S19

VII. Copies of NMR spectra S20

I. General remarks

NMR spectra were prepared on an Agilent 400-MR DD2 spectrometer (^{1}H NMR at 400 MHz, ^{13}C NMR at 100 MHz, ^{19}F NMR at 376 MHz). The ^{1}H NMR (400 MHz) chemical shifts and the ^{13}C NMR (100 MHz) chemical shifts were measured relative to $CDCl_3$ as the internal reference. High resolution mass spectra (HRMS) were prepared with a Shimadzu LCMS-IT-TOF (ESI) or an Agilent 1260/6530 (ESI). GC-MS spectra were recorded by an Agilent 8860-5977B.

Unless otherwise noted, all reagents were obtained from commercial suppliers and used without further purification. DCE were distilled over $CaCl_2$ before use. ^{t}BuNH$_2$, benzaldehydes (**1a-1e**, **1g-1r**, **1t),** BBr_3 (2*N* in DCM), BBr_3 (neat), CuI, $Pd(PPh_3)_4$ and Pd(dppf)Cl_2 were purchased from Energy Chemical. $NaBO_3{\cdot}4H_2O$ and $2Na_2CO_3{\cdot}3H_2O_2$ were purchased from Aladdin Chemical. $Pd(OAc)_2$ was purchased from Shanxi Kaida Chemical Engineering. 4-Benzylbenzaldehyde (**1f**)[1] and 9-methyl-9*H*-carbazole-2-carbaldehyde (**1r**)[2] were synthesized according to the literature. The yields of compound **2a** were determined by GC-MS analysis using calibration curves based on the data from authentic samples of the corresponding compound. For all GC-MS calibration curves, the ratio of molar concentration is taking as the horizontal axis and the ratio of GC area is taking as the vertical axis. Unless otherwise noted, all reactions were performed with dry solvents under argon in dried glassware with standard vacuum-line techniques.

II. General procedure for the optimization study

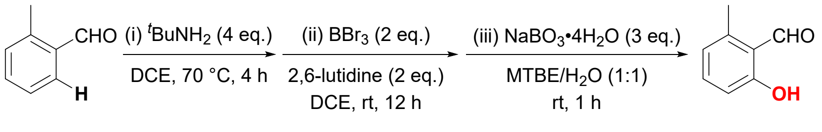

1a **2a**

(i) An oven-dried Schlenk tube equipped with a stirring bar was charged with 2-methylbenzaldehyde (48 mg, 0.4 mmol, 1 equiv), ^{t}BuNH$_2$ (168 μL, 1.6 mmol, 4 equiv) under argon. Then solvent DCE (1 mL) was added at room temperature. The reaction mixture was placed in a preheated oil bath at 70 °C, and stirred for 4 h.

(ii) Next, ^{t}BuNH$_2$ and solvent was removed under reduced pressure. Base (0.8 mmol, 2.0 equiv), DCE (1 mL) and BBr_3 (0.8 mmol, 2 equiv) were added under argon. The reaction mixture was stirred at rt for 12h.

(iii) Then, solvent was removed under reduced pressure, followed by the addition of

methyl *tert*-butyl ether (MTBE, 1 mL), sodium perborate tetrahydrate (185 mg, 1.2 mmol, 3.0 equiv), H_2O (1 mL) under argon. The reaction mixture was stirred at rt for another 1h. Afterwards, the reaction mixture was diluted with ethyl acetate (5 mL), mesitylene (55.6 μL, 0.4 mmol) was subjected as internal standard. The yield of product was determined by GC-MS analysis using standard calibration curves (Scheme S1) based on data from the authentic sample of **2a** and mesitylene.

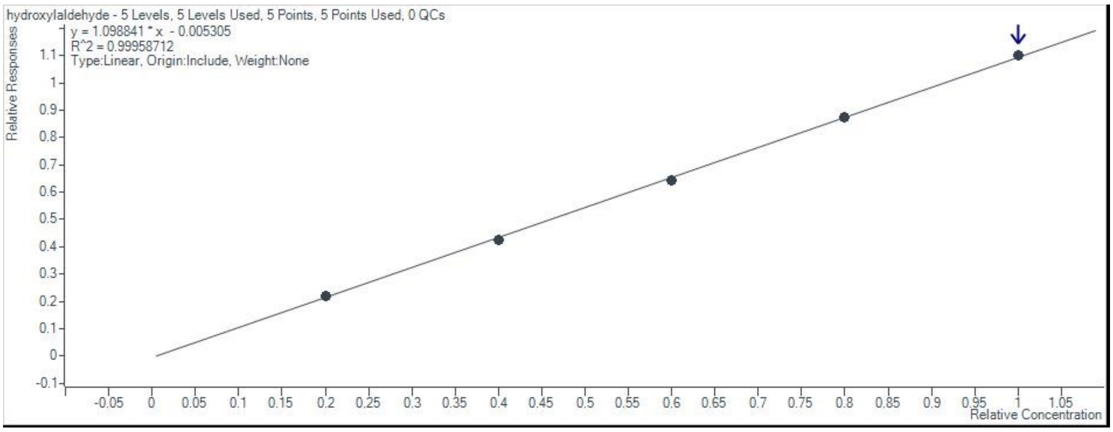

Sample					hydroxylaldehyde Results			
Name	Data File	Type	Level	Acq. Date-Time	RT	Area	Final Conc.	S/N
WRY-STD1	STDSIM1.D	Cal	1	2022/7/20 17:51	6.961	105163	1.0074505	34735.242
WRY-STD2	STDSIM2.D	Cal	2	2022/7/20 18:12	6.966	97328.6	0.8014354	12175.949
WRY-STD3	STDSIM3.D	Cal	3	2022/7/20 18:34	6.966	66091.2	0.58966234	6590.6087
WRY-STD4	STDSIM4.D	Cal	4	2022/7/20 18:54	6.966	44906	0.39139499	8088.5755
WRY-STD5	STDSIM5.D	Cal	5	2022/7/20 19:16	6.971	29166	0.20522891	2240.9359

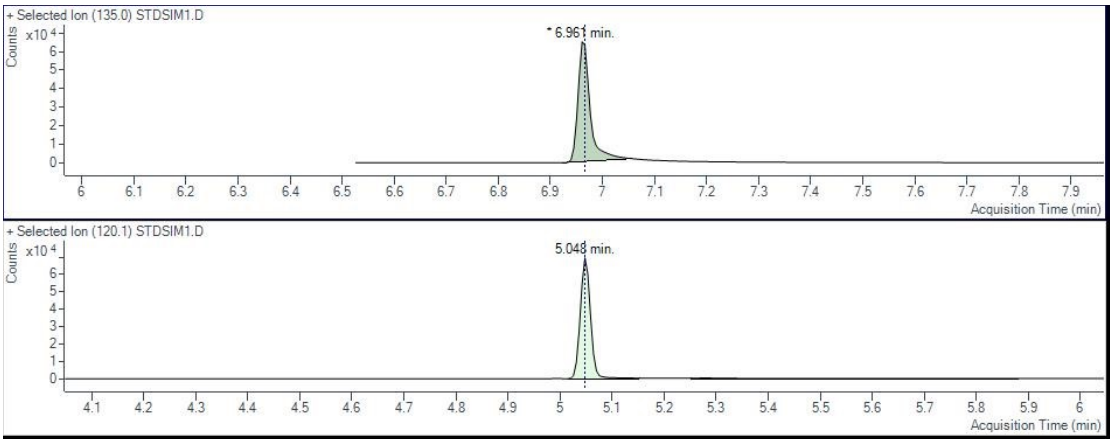

Scheme S1 Standard GC-MS calibration curves of **2a**

III. Experimental data for the described substances

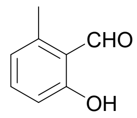

2-Hydroxy-6-methylbenzaldehyde (2a)

According to the general one-pot procedure for *ortho*-hydroxylation of benzaldehyde, purification by column chromatography (petroleum ether/ethyl acetate = 50/1) on silica gel afforded compound **2a** as colorless oil (43 mg, 79% yield).

^{1}H NMR (400 MHz, Chloroform-*d*) δ = 2.60 (s, 3H), 6.70 (d, J = 7.6 Hz, 1H), 6.80 (d, J = 8.4 Hz, 1H), 7.37 (t, J = 8.0 Hz, 1H), 10.31 (s, 1H), 11.91 (s, 1H) ppm.

^{13}C NMR (101 MHz, Chloroform-*d*) δ = 17.4, 115.7, 118.6, 121.9, 137.5, 142.2, 163.3, 194.7 ppm.

The NMR spectrum data are consistent with the literature.[3]

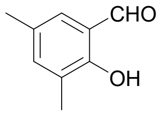

2-Hydroxy-3,5-dimethylbenzaldehyde (2b)

According to the general one-pot procedure for *ortho*-hydroxylation of benzaldehyde, purification by column chromatography (petroleum ether/ethyl acetate = 50/1) on silica gel afforded compound **2b** as yellow oil (47 mg, 78% yield).

^{1}H NMR (400 MHz, Chloroform-*d*) δ = 2.24 (s, 3H), 2.30 (s, 3H), 7.17 (s, 1H), 7.21 (s, 1H), 9.83 (s, 1H), 11.08 (s, 1H) ppm.

^{13}C NMR (101 MHz, Chloroform-*d*) δ = 15.1, 20.4, 119.9, 126.7, 128.7, 131.1, 139.2, 158.1, 196.8 ppm.

The NMR spectrum data are consistent with the literature.[4]

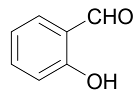

2-Hydroxybenzaldehyde (2c)

According to the general one-pot procedure for *ortho*-hydroxylation of benzaldehyde, purification by column chromatography (petroleum ether/ethyl acetate = 50/1) on silica

gel afforded compound **2c** as colorless oil (43 mg, 89% yield).

^{1}H NMR (400 MHz, Chloroform-*d*) δ = 6.95 – 7.04 (m, 2H), 7.49 – 7.57 (m, 2H), 9.88 (s, 1H), 11.02 (s, 1H) ppm.

^{13}C NMR (101 MHz, Chloroform-*d*) δ = 117.6, 119.9, 120.7, 133.8, 137.0, 161.6, 196.7 ppm.

The NMR spectrum data are consistent with the literature.[5]

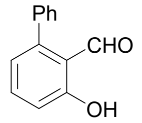

3-Hydroxy-[1,1'-biphenyl]-2-carbaldehyde (2d)

According to the general one-pot procedure for *ortho*-hydroxylation of benzaldehyde, purification by column chromatography (petroleum ether/ethyl acetate = 50/1) on silica gel afforded compound **2d** as pale yellow oil (50 mg, 63% yield).

^{1}H NMR (400 MHz, Chloroform-*d*) δ = 6.90 (dd, *J* = 7.6, 1.2 Hz, 1H), 7.00 (d, *J* = 8.4 Hz, 1H), 7.37 (m, 2H), 7.45 (m, 2H), 7.46 – 7.49 (m, 1H), 7.51 – 7.57 (t, *J* = 8.0 Hz, 1H), 9.84 (s, 1H), 11.93 (s, 1H) ppm.

^{13}C NMR (101 MHz, Chloroform-*d*) δ = 117.1, 118.1, 121.7, 128.4, 128.6, 130.2, 136.8, 137.5, 147.6, 162.9, 197.3 ppm.

The NMR spectrum data are consistent with the literature.[3]

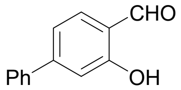

3-Hydroxy-[1,1'-biphenyl]-4-carbaldehyde (2e)

According to the general one-pot procedure for *ortho*-hydroxylation of benzaldehyde, purification by column chromatography (petroleum ether/ethyl acetate = 20/1) on silica gel afforded compound **2e** as a white solid (34 mg, 43% yield).

^{1}H NMR (400 MHz, Chloroform-*d*) δ 7.23 (s, 1H), 7.26 (dd, J = 8.0, 1.7 Hz, 1H), 7.42 – 7.45 (m, 1H), 7.46 – 7.50 (m, 2H), 7.60 – 7.63 (m, 2H), 7.64 (d, J = 1.7 Hz, 1H), 9.92 (s, 1H), 11.13 (s, 1H) ppm.

^{13}C NMR (101 MHz, Chloroform-*d*) δ 116.0, 119.0, 119.7, 127.5, 129.0, 129.1, 134.2, 139.4, 150.0, 162.0, 196.2 ppm.

Melting Point: 80-82 °C.

The analytical data are consistent with the literature.[3]

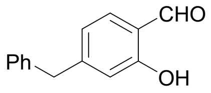

4-Benzyl-2-hydroxybenzaldehyde (2f)

According to the general one-pot procedure for *ortho*-hydroxylation of benzaldehyde, purification by column chromatography (petroleum ether/ethyl acetate = 80/1) on silica gel afforded compound **2f** as orange oil (52 mg, 61% yield).

^{1}H NMR (400 MHz, Chloroform-*d*) δ = 3.99 (s, 2H), 6.86 (m, 2H), 7.18 – 7.22 (m, 2H), 7.25 (t, *J* = 7.6 Hz, 1H), 7.28 – 7.35 (m, 2H), 7.46 (d, *J* = 8.0 Hz, 1H), 9.83 (s, 1H), 11.07 (s, 1H) ppm.

^{13}C NMR (101 MHz, Chloroform-*d*) δ = 42.4, 117.8, 119.1, 120.9, 126.7, 128.8, 129.1, 133.9, 139.4, 151.8, 161.9, 196.1 ppm.

The ^{1}H NMR spectrum data are consistent with the literature.[6]

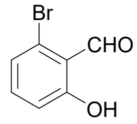

2-Bromo-6-hydroxybenzaldehyde (2g)

According to the general one-pot procedure for *ortho*-hydroxylation of benzaldehyde, purification by column chromatography (petroleum ether/ethyl acetate = 30/1) on silica gel afforded compound **2g** as a yellow solid (56 mg, 70% yield).

^{1}H NMR (400 MHz, Chloroform-*d*) δ 7.12 – 7.21 (m, 2H), 7.41 (d, *J* = 8.4 Hz, 1H), 9.86 (s, 1H), 11.12 (s, 1H) ppm.

^{13}C NMR (101 MHz, Chloroform-*d*) δ 119.7, 121.2, 123.7, 131.4, 134.7, 162.6, 195.9 ppm.

Melting Point: 53-56 °C.

The analytical data are consistent with the literature.[3]

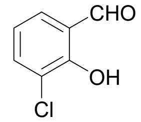

3-Chloro-2-hydroxybenzaldehyde (2h)

According to the general one-pot procedure for *ortho*-hydroxylation of benzaldehyde, purification by column chromatography (petroleum ether/ethyl acetate = 12/1) on silica gel afforded compound **2h** as an orange solid (18 mg, 30% yield).

^{1}H NMR (400 MHz, Chloroform-*d*) δ = 7.00 (t, J = 7.6 Hz, 1H), 7.51 (dd, J = 8.0, 1.6 Hz, 1H), 7.62 (dd, J = 8.0, 1.6 Hz, 1H), 9.90 (s, 1H), 11.49 (s, 1H) ppm.

^{13}C NMR (101 MHz, Chloroform-*d*) δ = 120.4, 121.6, 122.4, 132.2, 137.0, 157.3, 196.2 ppm.

Melting Point: 53-55 °C.

The analytical data are consistent with the literature.[7]

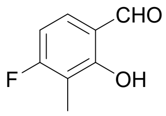

4-Fluoro-2-hydroxy-3-methylbenzaldehyde (2i)

According to the general one-pot procedure for *ortho*-hydroxylation of benzaldehyde, purification by column chromatography (petroleum ether/ethyl acetate = 20/1) on silica gel afforded compound **2i** as a white solid (32 mg, 52% yield).

^{1}H NMR (400 MHz, Chloroform-*d*) δ = 2.17 (d, J = 2.0 Hz, 3H), 6.71 (t, J = 8.8 Hz, 1H), 7.39 (dd, J = 8.8, 6.4 Hz, 1H), 9.81 (s, 1H), 11.61 (d, J = 2.0 Hz, 1H) ppm.

^{13}C NMR (101 MHz, Chloroform-*d*) δ = 7.0, 107.9 (d, $J_{C–F}$ = 25.3 Hz), 113.8 (d, $J_{C–F}$ = 24.2 Hz), 117.3 (d, $J_{C–F}$ = 2 Hz), 132.9 (d, $J_{C–F}$ = 13.1 Hz) 162.6 (d, $J_{C–F}$ = 12.1 Hz), 166.4 (d, $J_{C–F}$ = 256.5 Hz), 195.6 ppm.

^{19}F NMR (376 MHz, Chloroform-*d*) δ = -101.69 – -101.56 (m) ppm.

HRMS (ESI$^+$) calcd for $C_8H_8FO_2$ [M+H]$^+$ 155.0503, found 155.0505.

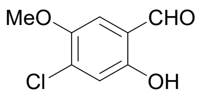

4-Chloro-2-hydroxy-3-methoxybenzaldehyde (2j)

According to the general one-pot procedure for *ortho*-hydroxylation of benzaldehyde, purification by column chromatography (petroleum ether/ethyl acetate = 8/1) on silica gel afforded compound **2j** as a pale yellow solid (29 mg, 39% yield).

^{1}H NMR (400 MHz, Chloroform-*d*) δ = 3.91 (s, 3H), 7.02 (s, 1H), 7.07 (s, 1H), 9.85 (s, 1H), 10.77 (s, 1H) ppm.

^{13}C NMR (101 MHz, Chloroform-*d*) δ = 56.9, 114.6, 118.9, 119.9, 133.3, 148.9, 156.2, 195.3 ppm.

The NMR spectrum data are consistent with the literature.[8]

Melting Point: 137-139 °C.

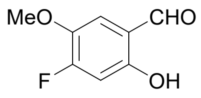

4-Fluoro-2-hydroxy-3-methoxybenzaldehyde (2k)

According to the general one-pot procedure for *ortho*-hydroxylation of benzaldehyde, purification by column chromatography (petroleum ether/ethyl acetate = 5/1) on silica gel afforded compound **2k** as a brown solid (42 mg, 62% yield).

^{1}H NMR (400 MHz, Chloroform-*d*) δ = 3.89 (s, 3H), 6.73 (d, J = 12.0 Hz, 1H), 7.07 (d, J = 8.0 Hz, 1H), 9.79 (s, 1H), 11.07 (s, 1H) ppm.

^{13}C NMR (101 MHz, Chloroform-*d*) δ = 57.1, 106.1 (d, $J_{C–F}$ = 21.7 Hz), 116.5 (d, $J_{C–F}$ = 2.5 Hz), 116.8 (d, J = 5.3 Hz, 1H), 142.0 (d, $J_{C–F}$ = 12.1 Hz), 158.1 (d, $J_{C–F}$ = 12.8 Hz), 158.7 (d, $J_{C–F}$ = 261.6 Hz), 194.9 ppm.

^{19}F NMR (376 MHz, Chloroform-*d*) δ = -117.92 (dd, J = 11.9, 9.2 Hz) ppm.

HRMS (ESI$^+$) calcd for $C_8H_7FO_3$ [M+Na]$^+$ 193.0271, found 193.0281.

Melting Point: 83-85 °C.

The analytical data are consistent with the literature.[3]

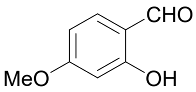

2-Hydroxy-4-methoxybenzaldehyde (2l)

According to the general one-pot procedure for *ortho*-hydroxylation of benzaldehyde, purification by column chromatography (petroleum ether/ethyl acetate = 20/1) on silica gel afforded compound **2l** as a yellow solid (18 mg, 30% yield).

^{1}H NMR (400 MHz, Chloroform-*d*) δ = 3.86 (s, 3H), 6.43 (d, *J* = 2.4 Hz, 1H), 6.54 (dd, *J* = 8.4, 2.4 Hz, 1H), 7.43 (d, *J* = 8.8 Hz, 1H), 9.71 (s, 1H), 11.48 (s, 1H) ppm.

^{13}C NMR (101 MHz, Chloroform-*d*) δ = 55.8, 100.1, 108.5, 114.4, 135.4, 164.7, 167.0, 194.5 ppm.

Melting Point: 40-42 °C.

The analytical data are consistent with the literature.[3]

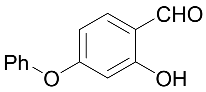

2-Hydroxy-4-phenoxybenzaldehyde (2m)

According to the general one-pot procedure for *ortho*-hydroxylation of benzaldehyde, purification by column chromatography (petroleum ether/ethyl acetate = 30/1) on silica gel afforded compound **2m** as brown oil (40 mg, 47% yield).

^{1}H NMR (400 MHz, Chloroform-*d*) δ = 6.41 (d, *J* = 2.4 Hz, 1H), 6.61 (dd, *J* = 8.4, 2.4 Hz, 1H), 7.10 (d, *J* = 7.6 Hz, 2H), 7.25 (t, *J* = 7.6 Hz, 1H), 7.39 – 7.45 (m, 2H), 7.47 (d, *J* = 8.4 Hz, 1H), 9.76 (s, 1H), 11.38 (s, 1H) ppm.

^{1}H NMR (101 MHz, Chloroform-*d*) δ = 104.5, 109.8, 109.9, 116.3, 121.0, 125.5, 130.3, 135.7, 154.4, 164.2, 165.7, 194.8 ppm.

The NMR spectrum data are consistent with the literature.[3]

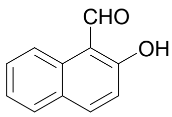

2-Hydroxy-1-naphthaldehyde (2n)

According to the general one-pot procedure for *ortho*-hydroxylation of benzaldehyde, purification by column chromatography (petroleum ether/ethyl acetate = 15/1) on silica gel afforded compound **2n** as a yellow solid (60 mg, 88% yield).

^{1}H NMR (400 MHz, Chloroform-*d*) δ = 7.17 (d, *J* = 7.6 Hz, 1H), 7.40 (d, *J* = 8.0 Hz, 1H), 7.46 – 7.58 (m, 2H), 8.02 (d, *J* = 7.2 Hz, 1H), 8.12 (d, *J* = 8.4 Hz, 1H), 9.84 (s, 1H), 11.65 (s, 1H) ppm.

^{13}C NMR (101 MHz, Chloroform-*d*) δ = 116.1, 120.6, 121.3, 124.4, 129.2, 132.5, 136.4, 139.3, 143.1, 155.3, 198.0 ppm.

Melting Point: 79-82 °C.

The analytical data are consistent with the literature.[9]

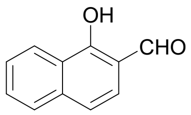

1-Hydroxy-2-naphthaldehyde (2o)

According to the general one-pot procedure for *ortho*-hydroxylation of benzaldehyde, purification by column chromatography (petroleum ether/ethyl acetate = 30/1) on silica gel afforded compound **2o** as a dark green solid (30 mg, 42% yield).

^{1}H NMR (400 MHz, Chloroform-*d*) δ 7.38 (d, *J* = 8.4 Hz, 1H), 7.50 (d, *J* = 8.8 Hz, 1H), 7.52 – 7.59 (m, 1H), 7.67 (t, *J* = 7.6 Hz, 1H), 7.79 (d, *J* = 8.0 Hz, 1H), 8.45 (d, *J* = 8.4 Hz, 1H), 9.98 (s, 1H), 12.66 (s, 1H) ppm.

^{13}C NMR (101 MHz, Chloroform-*d*) δ 114.0, 119.6, 124.5, 124.7, 126.3, 126.6, 127.8, 130.8, 137.7, 162.1, 196.5 ppm.

Melting Point: 56-58 °C.

The analytical data are consistent with the literature.[9]

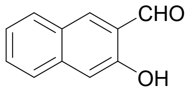

3-Hydroxy-2-naphthaldehyde(2o’)

According to the general one-pot procedure for *ortho*-hydroxylation of benzaldehyde, purification by column chromatography (petroleum ether/ethyl acetate = 30/1) on silica gel afforded compound **2o’** as a yellow solid (21 mg, 30% yield).

^{1}H NMR (400 MHz, Chloroform-*d*) δ = 7.28 (s, 1H), 7.38 (t, *J* = 7.2 Hz, 1H), 7.57 (t, *J* = 6.8 Hz, 1H), 7.72 (d, *J* = 8.4 Hz, 1H), 7.87 (d, *J* = 8.0 Hz, 1H), 8.15 (s, 1H), 10.09 (s, 1H), 10.32 (s, 1H) ppm.

^{13}C NMR (101 MHz, Chloroform-*d*) δ = 112.1, 122.5, 124.6, 126.8, 127.6, 129.5, 130.4, 138.0, 138.4, 156.0, 196.8 ppm.

Melting Point: 97-99 °C.

The analytical data are consistent with the literature.[3]

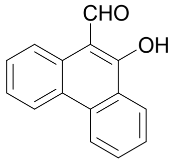

10-Hydroxyphenanthrene-9-carbaldehyde (2p)

According to the general one-pot procedure for *ortho*-hydroxylation of benzaldehyde, purification by column chromatography (petroleum ether/ethyl acetate = 10/1) on silica gel afforded compound **2p** as a yellow solid (50 mg, 56% yield).

^{1}H NMR (400 MHz, Chloroform-*d*) δ = 7.23 (d, *J* = 8.0 Hz, 1H), 7.64 (m, 2H), 7.82 (t, *J* = 7.6 Hz, 1H), 7.97 (d, *J* = 8.0 Hz, 1H), 8.21 (d, *J* = 8.4 Hz, 1H), 8.32 (s, 1H), 8.63 (d, *J* = 8.4 Hz, 1H), 9.88 (s, 1H), 11.65 (s, 1H) ppm.

^{13}C NMR (101 MHz, Chloroform-*d*) δ = 114.6, 116.8, 117.4, 123.9, 127.8, 129.4, 129.7, 130.7, 131.8, 131.9, 133.3, 134.6, 147.8, 156.3, 197.8 ppm.

The NMR spectrum data are consistent with the literature.[10]

Melting Point: 133-136 °C.

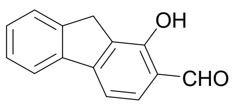

3-Hydroxy-9*H*-fluorene-2-carbaldehyde (2q)

According to the general one-pot procedure for *ortho*-hydroxylation of benzaldehyde, purification by column chromatography (petroleum ether/ethyl acetate = 10/1) on silica gel afforded compound **2q** as a white solid (55 mg, 66% yield).

^{1}H NMR (400 MHz, Chloroform-*d*) δ = 3.90 (s, 2H), 7.39 – 7.48 (m, 3H), 7.57 (d, *J* = 8.0 Hz, 1H), 7.59 – 7.64 (m, 1H), 7.79 – 7.86 (m, 1H), 9.91 (s, 1H), 11.35 (s, 1H) ppm.

^{13}C NMR (101 MHz, Chloroform-*d*) δ = 33.8, 112.1, 119.4, 121.4, 125.6, 127.1, 128.9, 130.4, 134.8, 140.4, 145.1, 150.9, 158.3, 196.3 ppm.

HRMS (ESI$^+$) calcd for $C_{14}H_{11}O_2$ [M+H]$^+$ 211.0754, found 211.0753.

Melting Point: 92-95 °C.

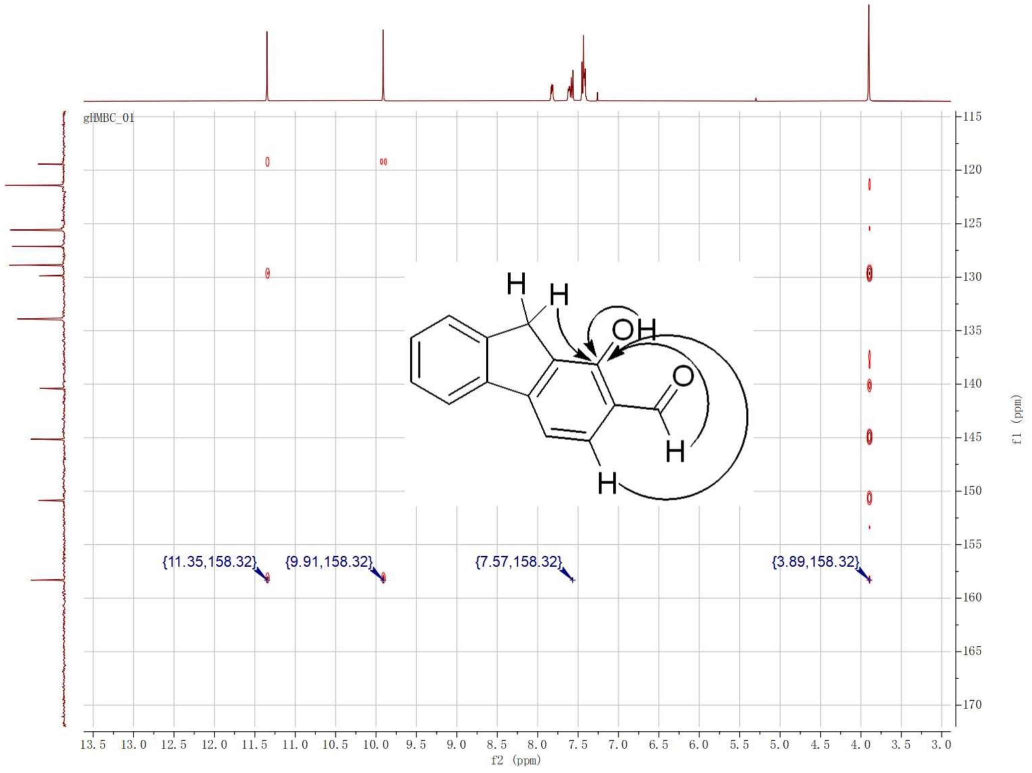

Figure S1 Fragment of HMBC spectrum of **2q**

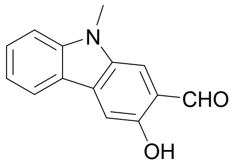

2-Hydroxy-9-methyl-9*H*-carbazole-2-carbaldehyde (2r)

According to the general procedure, purification by column chromatography (petroleum ether/ethyl acetate = 10/1) on silica gel afforded compound **2r** as a yellow solid (31 mg, 34% yield).

^{1}H NMR (400 MHz, Chloroform-*d*) δ = 3.81 (s, 3H), 7.21 (t, J = 8.0 Hz, 1H), 7.34 (d, J = 8.4 Hz, 1H), 7.43 (s, 1H), 7.55 (ddt, J = 8.4, 7.2, 1.2 Hz, 1H), 7.58 (s, 1H), 8.05 (d, J = 8 Hz, 1H), 9.99 (s, 1H), 10.76 (s, 1H) ppm.

^{13}C NMR (101 MHz, Chloroform-*d*) δ = 29.4, 107.0, 109.0, 113.6, 118.9, 119.2, 121.5, 122.1, 128.7, 131.2, 135.7, 144.5, 154.9, 196.1 ppm.

HRMS (ESI$^+$) calcd for $C_{14}H_{11}NO_2$ $[M+H]^+$ 226.0863, found 226.0858.

Melting Point: 113-116 °C.

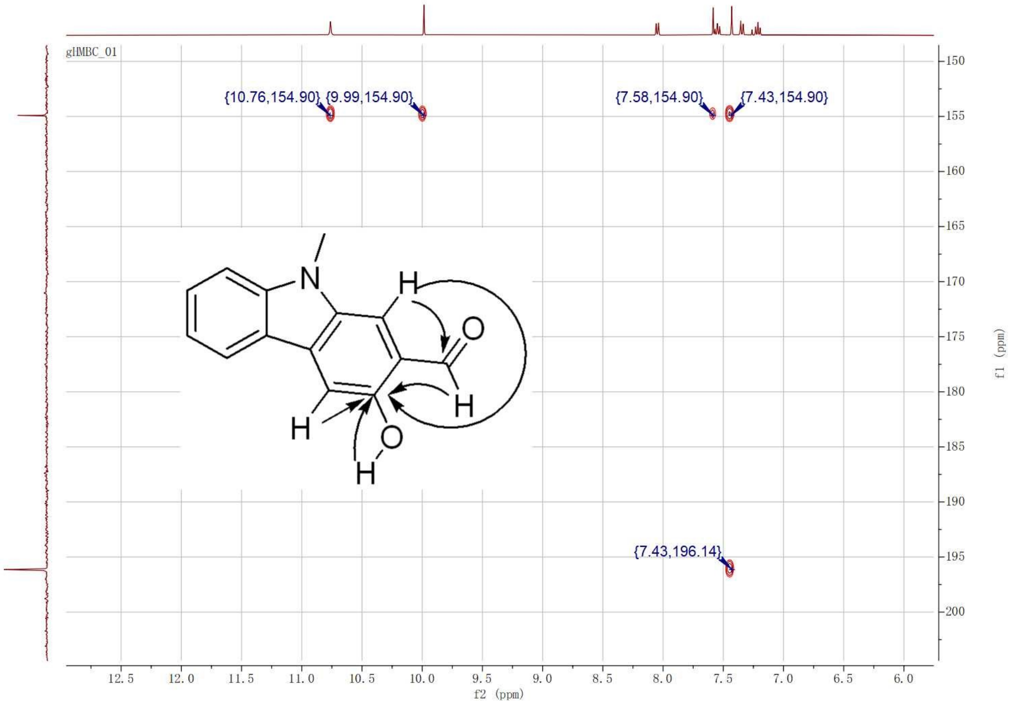

Figure S2 Fragment of HMBC spectrum of **2r**

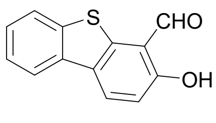

3-Hydroxydibenzo[*b,d*]thiophene-4-carbaldehyde (2s)

According to the general procedure, purification by column chromatography (petroleum ether/ethyl acetate = 20/1) on silica gel afforded compound **2s** as a brown solid (34 mg, 37% yield).

^{1}H NMR (400 MHz, Chloroform-*d*) δ = 7.06 (d, *J* = 8.8 Hz, 1H), 7.42 – 7.51 (m, 2H), 7.85 (d, *J* = 6.8 Hz, 1H), 8.01 (d, *J* = 8.0 Hz, 1H), 8.18 (d, *J* = 8.8 Hz, 1H), 10.34 (s, 1H), 11.57 (s, 1H) ppm.

^{13}C NMR (101 MHz, Chloroform-*d*) δ = 114.5, 115.9, 120.9, 122.9, 125.3, 126.4, 129.3, 129.8, 134.7, 137.6, 143.7, 163.1, 192.9 ppm.

HRMS (ESI$^+$) calcd for $C_{13}H_8O_2S$ $[M+H]^+$ 229.0318, found 229.0321.

Melting Point: 176-178 °C.

IV. Unsuccessful substrates

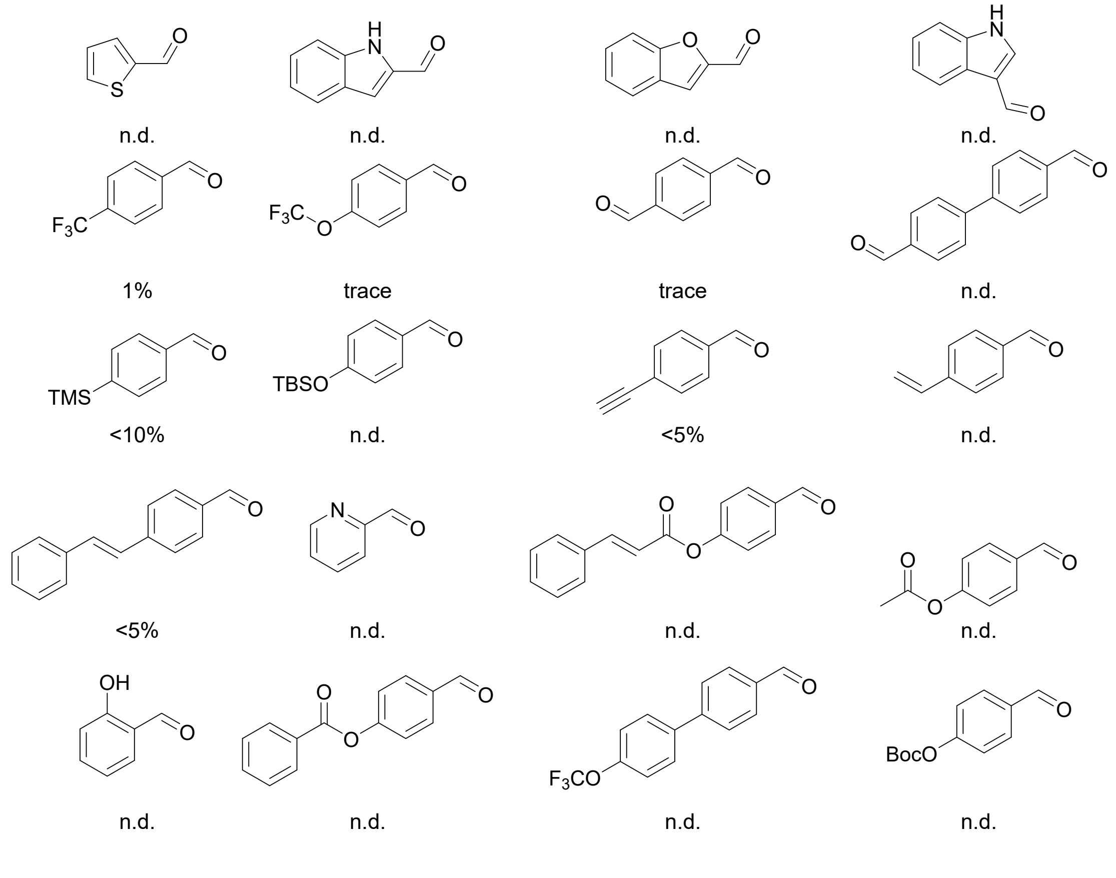

V. Scale-up reaction and downstream synthesis

(1) Scale-up synthesis of 2c/2g

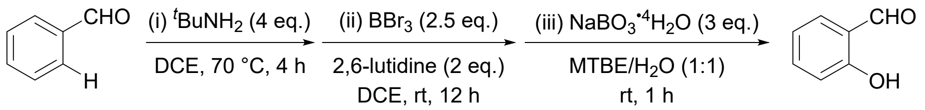

1c / 1g **2c / 2g**

(i) An oven-dried 100 mL Schlenk flask equipped with a stirring bar was charged with benzaldehyde **1** (8 mmol, 1.0 equiv), ^{t}BuNH$_2$ (3.4 mL, 4.0 equiv) under argon. Then solvent DCE (20 mL) was added at room temperature. The reaction mixture was placed in a preheated oil bath at 70 °C, and stirred for 4 h.

(ii) Next, ^{t}BuNH$_2$ and solvent was removed under reduced pressure. 2,6-Lutidine (1.72 g, 2.0 equiv), then a mixture of DCE (20 mL) and BBr_3 (10 mL, 2*N* in DCM, 2.5 equiv) were added dropwisely under Ar. The reaction mixture was stirred at rt for 12h.

(iii) Then, solvent was removed under reduced pressure, followed by the addition of MTBE (20 mL), sodium perborate tetrahydrate(3.7 g, 16 mmol, 2.0 equiv) and water (20 mL) under argon. The reaction mixture was stirred at rt for another 1 h. Afterwards, the reaction mixture was extracted with ethyl acetate (100 mL×3). The combined organic layer was further purified by column chromatography on silica gel (petroleum ether/ethyl acetate = 50/1) to afford the corresponding product **2c** or **2g**.

(2) Synthesis of 2*H*-chromen-2-one (3)

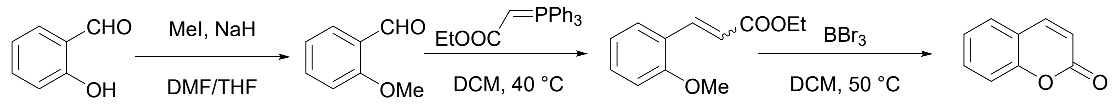

2c **3**

A modified literature procedure was used for the synthesis of **3**:[3]

To a 100 mL flask were added NaH (0.12 g, 5.0 mmol), DMF (8.0 mL), and THF (2.0 mL), then cooled to 0 °C, followed by dropwise addition of a mixture of **2c** (0.61 g, 5.0 mmol) and MeI (2.13 g, 15.0 mmol) in THF (5.0 mL). The mixture was stirred for 2 h at 0 °C, and then the reaction was allow to warm to rt and stir for another 3 h. The reaction was quenched with H_2O and extracted with EA (3 × 20 mL). The combined organic phase was washed with brine, dried over Na_2SO_4 and purified by flash chromatography on silica gel (petroleum ether/ethyl acetate = 20/1) to yield the

methylated product *o*-anisaldehyde as colorless oil.

The crude *o*-anisaldehyde was dissolved in CH_2Cl_2 (10 mL), Ph_3P=CHCOOEt (2.1 g, 6 mmol) was added. The mixture was stirred at 40 °C for 4 h. The reaction was quenched with H_2O and extracted with CH_2Cl_2 (3 × 15 mL). Purification by flash chromatography on silica gel (petroleum ether/ethyl acetate = 12/1) gave crude ethyl 3-(2-methoxyphenyl)acrylate as colorless oil.

The crude ethyl 3-(2-methoxyphenyl) acrylate was directly transferred to a 100 mL Schlenk tube under Ar, followed by the addition of CH_2Cl_2 (15.0 mL) and BBr_3 (4 mmol, 1*N* in CH_2Cl_2). The mixture was stirred at 50 °C for 4 h. After cooling to rt, the reaction was quenched with H_2O (20 mL), extracted with EA (3×20 mL), washed with brine, and dried over Na_2SO_4. Purification by column chromatography on silica gel (petroleum ether/ethyl acetate = 5/1) gave **3** (446 mg, 61% overall yield) as a white solid.

^{1}H NMR (400 MHz, Chloroform-*d*) δ = 6.42 (d, *J* = 9.6 Hz, 1H), 7.25 – 7.33 (m, 2H), 7.44 – 7.56 (m, 2H), 7.71 (d, *J* = 9.6 Hz, 1H) ppm.

^{13}C NMR (101 MHz, Chloroform-*d*) δ = 116.8, 117.0, 118.9, 124.5, 128.0, 131.9, 143.6, 154.1, 160.9 ppm.

Melting Point: 76-78 °C.

The analytical spectrum data are consistent with the literature.[3]

(3) Synthesis of ethyl 2-oxo-2H-chromene-3-carboxylate (4)

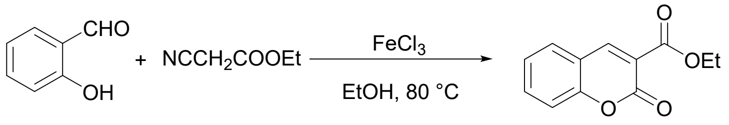

2c **4**

A modified literature procedure was used for the synthesis of **4**:[11]

To a stirred solution of salicyladehyde **2c** (49 mg, 0.4 mmol) and ethyl cyanoacetate (68 mg, 0.6 mmol) in ethanol (2 mL) was added anhydrous $FeCl_3$ (5 mg, 0.03mmol). The mixture was heated in an oil bath at 80 °C for 24 h and cooled down to room temperature. The reaction was quenched with H_2O (5 mL), extracted with ethyl acetate (3×5 mL), washed with brine, and dried over Na_2SO_4. Purification by column chromatography on silica gel (petroleum ether/ethyl acetate = 8/1) gave **4** (47.9 mg, 55%) as a white solid.

^1H NMR (400 MHz, Chloroform-*d*) δ = 1.41 (t, *J* = 6.8 Hz, 3H), 4.41 (q, *J* = 7.2 Hz, 2H), 7.30 – 7.38 (m, 2H), 7.59 – 7.68 (m, 2H), 8.53 (s, 1H) ppm.

^{13}C NMR (101 MHz, Chloroform-*d*) δ = 14.4, 61.7, 116.6, 118.0, 118.4, 125.7, 128.6, 134.5, 148.2, 154.9, 156.7, 163.2 ppm.

Melting Point: 94-96 °C.

The analytical data are consistent with the literature.[11]

(4) Synthesis of ethyl 2-oxo-2H-chromene-3-carboxylate (5)

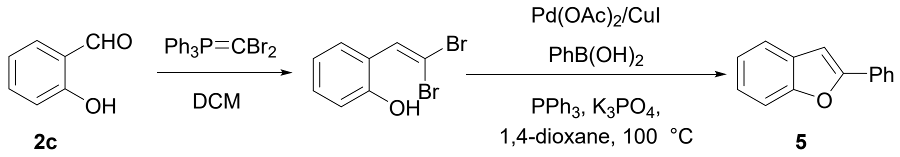

A modified literature procedure was used for the synthesis of **5**:[3]

To a 25 mL flask were added PPh_3 (1.57 g, 6 mmol) and CH_2Cl_2 (6 mL). The mixture was cooled to 0 °C, after which CBr_4 (1 g, 3 mmol) dissolved in CH_2Cl_2 (3 mL) was added and stirred for 10 min. NEt_3 (0.61 g, 6 mmol) was added dropwise and stirred for an additional 5 min, after which **2c** (122 mg, 1 mmol, 1 equiv) in CH_2Cl_2 (1 mL) was added dropwise. The mixture was stirred for another 30 min at 0 °C, then warmed to rt and stirred for 2 h. The reaction was quenched by saturated aqueous NH_4Cl solution (10 mL), extracted with CH_2Cl_2 (3×10 mL). The combined organic layers were concentrated and purified by flash chromatography on silica gel (petroleum ether/ethyl acetate = 8/1) to give the crude 2-(2,2-dibromovinyl)phenol as a white solid.

The crude 2-(2,2-dibromovinyl)phenol was transferred to a Schlenk tube, followed by the addition of $PhB(OH)_2$ (244 mg, 2 mmol), $Pd(OAc)_2$ (11.3 mg, 5.0 mol %), CuI (9.5 mg, 5.0 mol %), PPh_3 (39.3 mg, 15 mol %), K_3PO_4 (254 mg, 1.2 mmol), and 1,4-dioxane (10 mL) under Ar. The mixture was stirred at 100 °C for 24 h, extracted with EtOAc (10 mL), washed with brine, and dried over Na_2SO_4. Purification by column chromatography on silica gel (petroleum ether) gave **5** (126 mg, 65% overall yield) as a white solid.

^1H NMR (400 MHz, Chloroform-*d*) δ = 7.05 (s, 1H), 7.25 (t, *J* = 7.6 Hz, 1H), 7.28 – 7.33 (m, 1H), 7.37 (t, *J* = 7.2 Hz, 1H), 7.47 (t, *J* = 7.6 Hz, 2H), 7.55 (d, *J* = 7.6 Hz, 1H), 7.60 (d, *J* = 7.2 Hz, 1H), 7.89 (d, *J* = 6.8 Hz, 2H) ppm.

^{13}C NMR (101 MHz, Chloroform-*d*) δ = 101.0, 111.3, 121.0, 123.1, 124.4, 125.1, 127.6,

128.9, 129.3, 130.6, 155.0, 156.0 ppm.

Melting Point: 119-121 °C.

The analytical data are consistent with the literature.[3]

(5) Synthesis of 2-Hydroxy-6-((triisopropylsilyl)ethynyl)benzaldehyde (6)

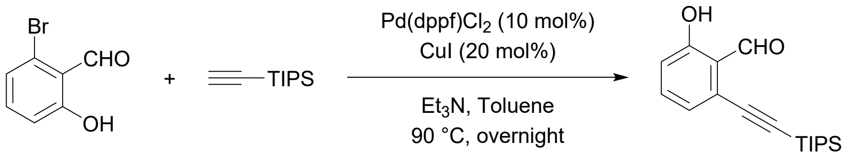

An oven-dried Schlenk tube equipped with a stirring bar was charged with 2-Bromo-6-hydroxybenzaldehyde (201 mg, 1 mmol), Pd(dppf)Cl_2 (73 mg, 0.1 mmol), (triisopropylsilyl)acetylene (673 μL, 3 mmol), CuI (57 mg, 0.3 mmol) and triethylamine (4 mL) under argon. Then solvent toluene (15 mL) was added under argon at room temperature. The reaction was stirred at 90 °C overnight. The crude solution was then taken up in CH_2Cl_2, washed with water, dried over $MgSO_4$, and concentrated in vacuo. The crude residue was purified by column chromatography on silica gel (petroleum ether/dichloromethane = 4/1) to afford 2-hydroxy-6-((triisopropylsilyl)ethynyl)benzaldehyde as a pale yellow solid (199.6 mg, 66% yield).

^{1}H NMR (400 MHz, Chloroform-*d*) δ = 1.11 – 1.15 (m, 21H), 6.94 (d, *J* = 8.4 Hz, 1H), 7.10 (d, *J* = 7.6 Hz, 1H), 7.43 (dd, *J* = 8.4, 7.6 Hz, 1H), 10.50 (s, 1H), 11.66 (s, 1H) ppm.

^{13}C NMR (101 MHz, Chloroform-*d*) δ = 11.4, 18.8, 99.5, 101.7, 118.5, 119.9, 125.3, 127.7, 136.7, 162.4, 197.0 ppm.

The NMR spectrum data are consistent with the literature.[12]

Melting Point: 38-39 °C.

(6) Synthesis of 3-Hydroxy-2'-isopropyl-[1,1'-biphenyl]-2-carbaldehyde (7)

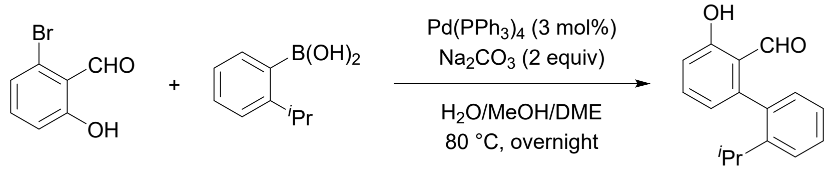

An oven-dried Schlenk tube was charged with a stirring bar, 2-Bromo-6-

hydroxybenzaldehyde (201 mg, 1 mmol), 2-isopropylphenylboronic acid (180.4 mg, 1.1 mmol), $Pd(PPh_3)_4$ (35 mg, 0.03 mmol), and Na_2CO_3 (212 mg, 2 mmol) under argon. Then H_2O (1 mL), MeOH (1 mL), and DME (3 mL) was added. The reaction mixture was stirred at 80 °C overnight. After cooling to rt, the mixture was quenched with H_2O (5 mL) and extracted with CH_2Cl_2 (3×10 mL). The combined organic layers were dried over Na_2SO_4, concentrated, and purified by column chromatography on silica gel (petroleum ether/dichloromethane = 2/1) to afford 3-hydroxy-2'-isopropyl-[1,1'-biphenyl]-2-carbaldehyde as a pale yellow solid (165.8 mg, 69% yield).

^{1}H NMR (400 MHz, Chloroform-*d*) δ = 1.14 (td, *J* = 6.9, 1.8 Hz, 6H), 2.67 – 2.86 (m, 1H), 6.80 (d, *J* = 7.4 Hz, 1H), 7.01 (d, *J* = 8.5 Hz, 1H), 7.15 (d, *J* = 7.7 Hz, 1H), 7.21 – 7.27 (m, 1H), 7.40 – 7.46 (m, 2H), 7.53 (t, *J* = 7.9 Hz, 1H), 9.54 – 9.67 (m, 1H), 11.79 – 11.87 (m, 1H) ppm.

^{13}C NMR (101 MHz, Chloroform-*d*) δ = 23.6, 24.6, 30.2, 117.0, 118.9, 121.7, 125.4, 125.7, 128.9, 130.2, 135.8, 136.6, 146.9, 147.3, 162.6, 197.1 ppm.

The NMR spectrum data are consistent with the literature.[13]

Melting Point: 60-62°C.

(7) Synthesis of 5-Bromo-2-oxo-2*H*-chromene-3-carboxylic acid (8)

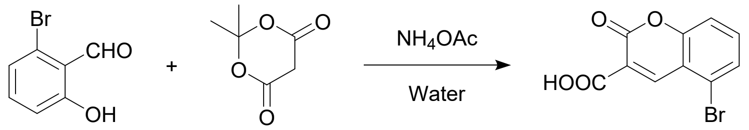

2-Bromo-6-hydroxybenzaldehyde (201 mg, 1 mmol) and 2,2-dimethyl-1,3-dioxane-4,6-dione (171 mg, 1.5 mmol) were mixed in 15 mL water at room temperature. Catalytic amount of ammonium acetate were added, and the reaction mixture was stirred under the room temperature for 3 h. Hydrochloric acid solution (2*N*) was added to adjust pH to 4.5. The resulted solid was filtered, washed three times with 5 mL of methanol, and dried under vacuum at 50 °C to afford 5-bromo-2-oxo-2*H*-chromene-3-carboxylic acid as a white solid (242 mg, 90% yield).

^{1}H NMR (400 MHz, DMSO-d_6) δ = 7.44 – 7.49 (m, 1H), 7.64 (t, *J* = 8.1 Hz, 1H), 7.70 (dd, *J* = 8.0, 1.2 Hz, 1H), 8.60 (s, 1H), 13.53 (br s, 1H) ppm.

^{13}C NMR (101 MHz, DMSO-d_6) δ = 116.3, 117.6, 119.7, 123.0, 128.7, 135.1, 145.7, 155.3, 155.8, 163.6 ppm.

The NMR spectrum data are consistent with the literature.[14]

Melting Point: 249-252°C.

VI. References

1. G. W. Gribble and C. F. Nutaitis, [1.1.1.1.1] paracyclophane and [1.1.1.1.1.1] paracyclophane, *Tetrahedron Lett.*, 1985, **26**, 6023-6026.
2. L. Bu, Y. Li, J. Wang, M. Sun, M. Zheng, W. Liu, S. Xue and W. Yang, Synthesis and piezochromic luminescence of aggregation-enhanced emission 9,10-bis (*N*-alkylcarbazol-2-yl-vinyl-2) anthracenes, *Dyes Pigm.*, 2013, **99**, 833-838.
3. F. Yang, K. Rauch, K. Kettelhoit and L. Ackermann, Aldehyde-assisted Ruthenium(II)-catalyzed C–H Oxygenations, *Angew. Chem., Int. Ed.*, 2014, **53**, 11285-11288.
4. L. Ochmann, M. Fuhrmann, F. J. Gössl, A. Makaveev and P. R. Schreiner, All That *metas*—Synthesis of Dispersion Energy Donor-Substituted Benzenes, *Org. Lett.*, 2022, **24**, 6968-6972.
5. B. Kokić, Ž. Selaković, A. M. Nikolić, A. Andrijević, B. Anđelković, V. Ajdačić and I. M. Opsenica, Low-Valent Cobalt-Catalyzed Deprotection of Allyloxyarenes, *Eur. J. Org. Chem.*, 2022, **2022**, e202201112.
6. D. Ding, Y. Zhao, Q. Meng, D. Xie, B. Nare, D. Chen, C. J. Bacchi, N. Yarlett, Y.-K. Zhang and V. Hernandez, Discovery of novel benzoxaborole-based potent antitrypanosomal agents, *ACS Med. Chem. Lett.*, 2010, **1**, 165-169.
7. N. I. Saper and B. B. Snider, 2, 2, 6, 6-Tetramethylpiperidine-catalyzed, *ortho*-selective chlorination of phenols by sulfuryl chloride, *J. Org. Chem.*, 2014, **79**, 809-813.
8. X. Y. Chen, S. Ozturk and E. J. Sorensen, Pd-Catalyzed *Ortho* C–H Hydroxylation of Benzaldehydes Using a Transient Directing Group, *Org. Lett.*, 2017, **19**, 6280-6283.
9. S. Saha, A. A. Bhosle, A. Chatterjee and M. Banerjee, Mechanochemical Duff Reaction in Solid Phase for Easy Access to Mono-and Di-formyl Electron-Rich Arenes, *J. Org. Chem.*, 2023, **88**, 10002-10013.
10. A. Skotnicka, E. Kolehmainen, K. Laihia and R. Gawinecki, NMR studies of benzoannulation in lithium, sodium and potassium *ortho*-formylphenolates, *J. Mol. Struct.*, 2010, **977**, 12-16.
11. X. He, Z. Yan, X. Hu, Y. Zuo, C. Jiang, L. Jin and Y. Shang, $FeCl_3$-catalyzed cascade reaction: An efficient approach to functionalized coumarin derivatives, *Synth. Commun.*, 2014, **44**, 1507-1514.
12. T. Pariat, M. Munch, M. Durko‐Maciag, J. Mysliwiec, P. Retailleau, P. M. Vérité, D. Jacquemin, J. Massue and G. Ulrich, Impact of Heteroatom Substitution on Dual‐State Emissive Rigidified 2‐(2'‐hydroxyphenyl) benzazole Dyes: Towards Ultra‐Bright ESIPT Fluorophores, *Chemistry –A European Journal*, 2021, **27**, 3483-3495.
13. S. T. Linde, V. Corti, V. H. Lauridsen, J. N. Lamhauge, K. A. Jørgensen and N. M. Rezayee, Atroposelective brominations to access chiral biaryl scaffolds using high-valent Pd-catalysis, *Chem. Sci.*, 2023, **14**, 3676-3681.
14. A. Chilin, R. Battistutta, A. Bortolato, G. Cozza, S. Zanatta, G. Poletto, M. Mazzorana, G. Zagotto, E. Uriarte and A. Guiotto, Coumarin as attractive casein kinase 2 (CK2) inhibitor scaffold: an integrate approach to elucidate the putative binding motif and explain structure–activity relationships, *J. Med. Chem.*, 2008, **51**, 752-759.

VII. Copies of NMR spectra

^{1}H NMR spectra of compound **2a**:

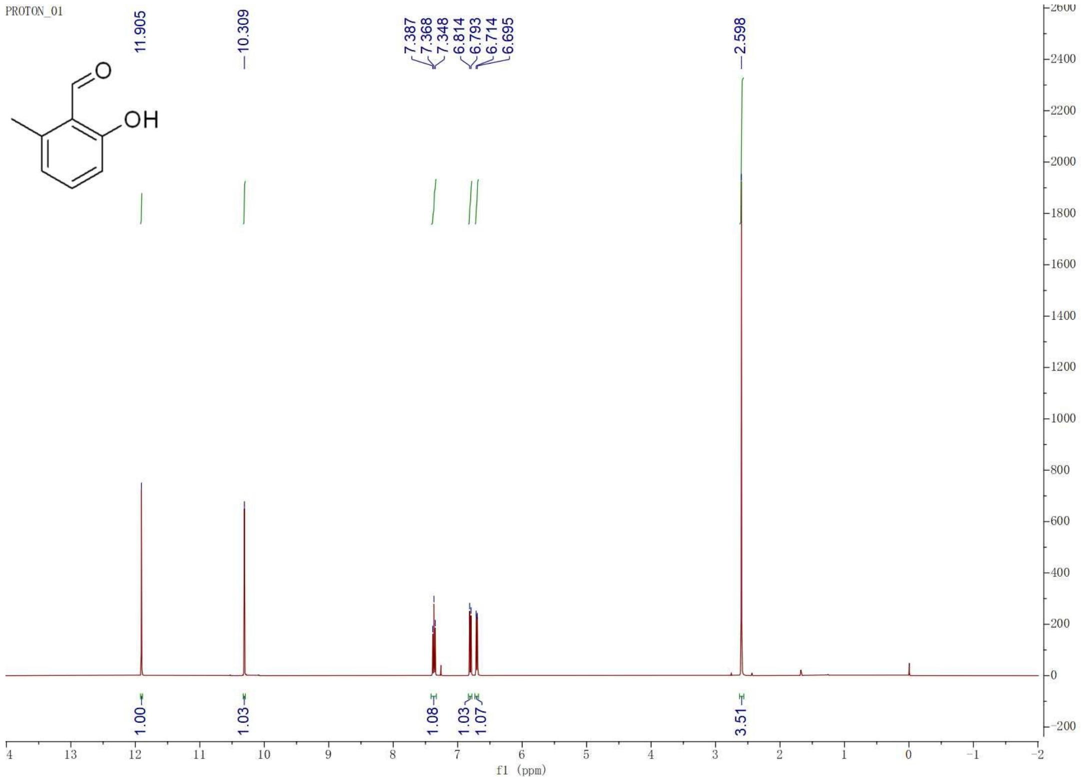

^{13}C NMR spectra of compound **2a**:

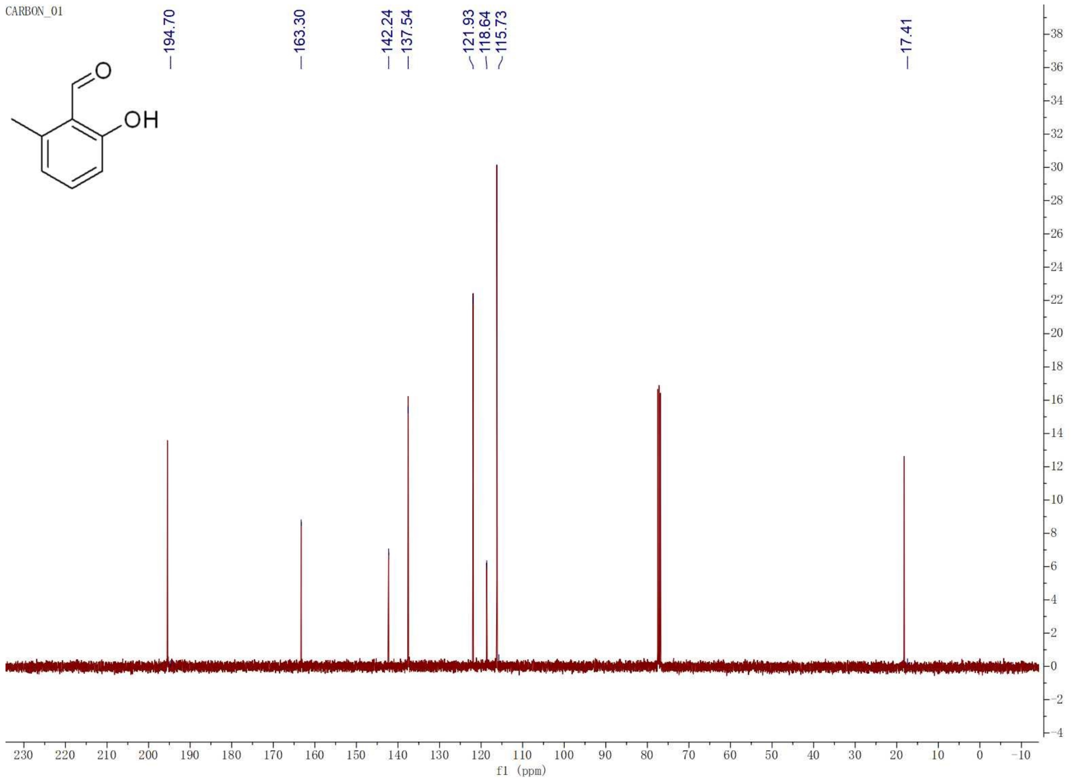

^{1}H NMR spectra of compound **2b**:

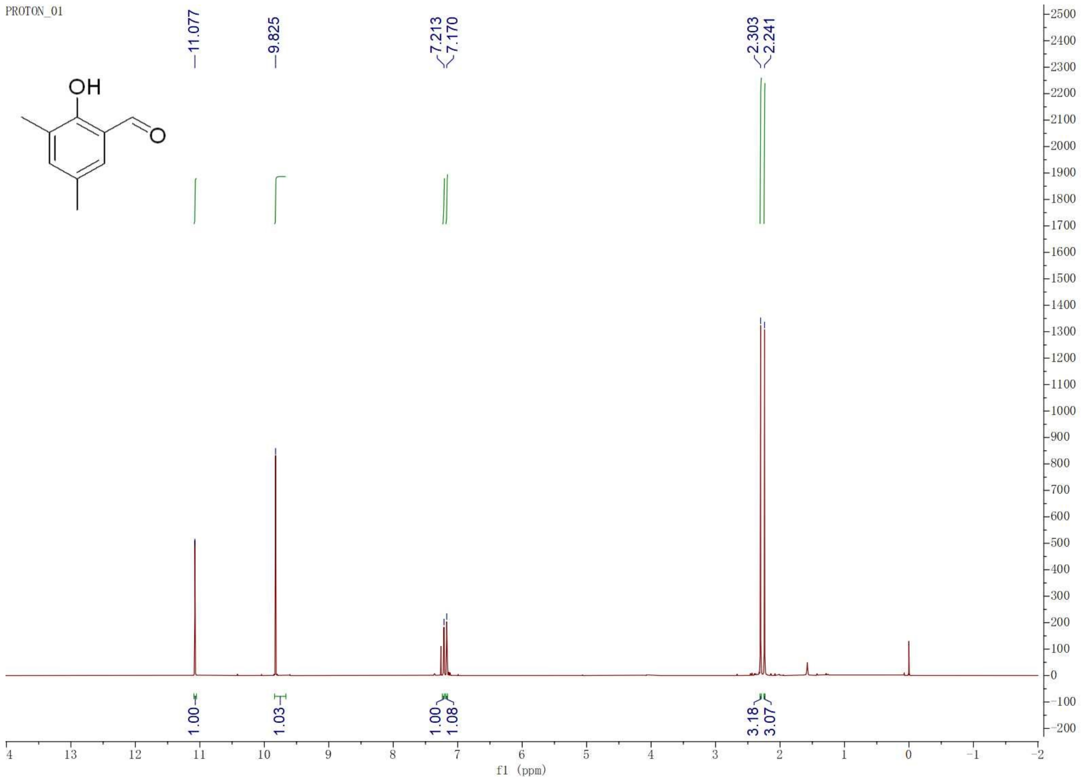

^{13}C NMR spectra of compound **2b**:

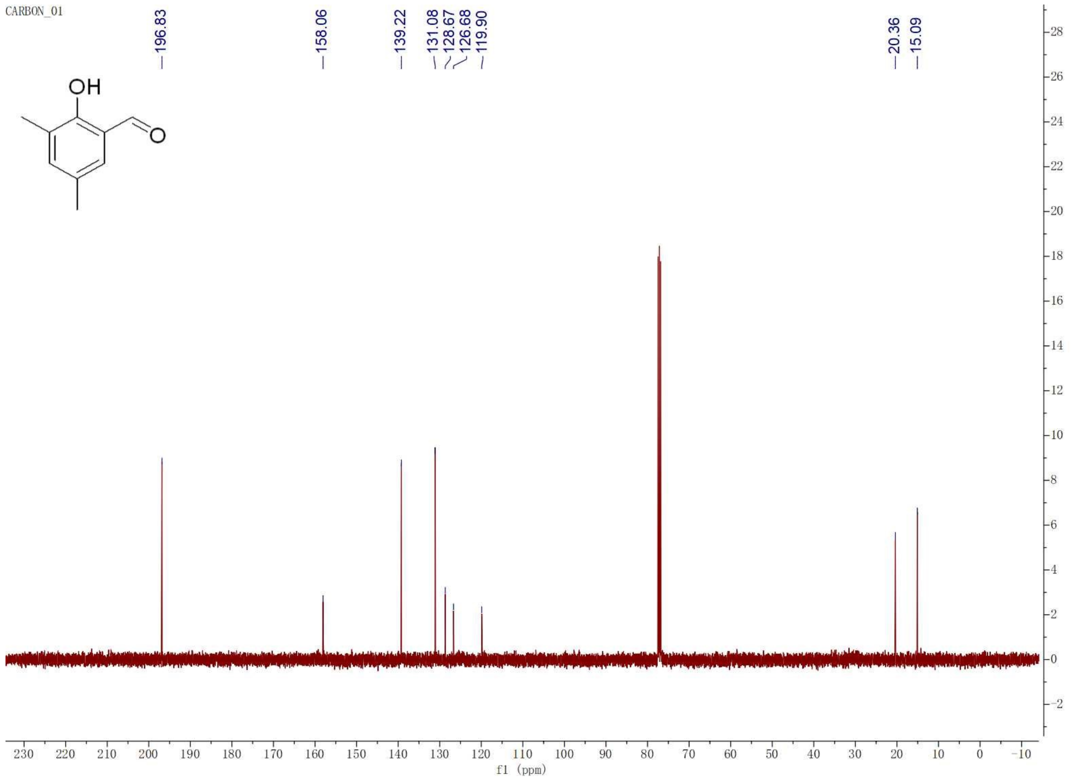

^{1}H NMR spectra of compound **2c**:

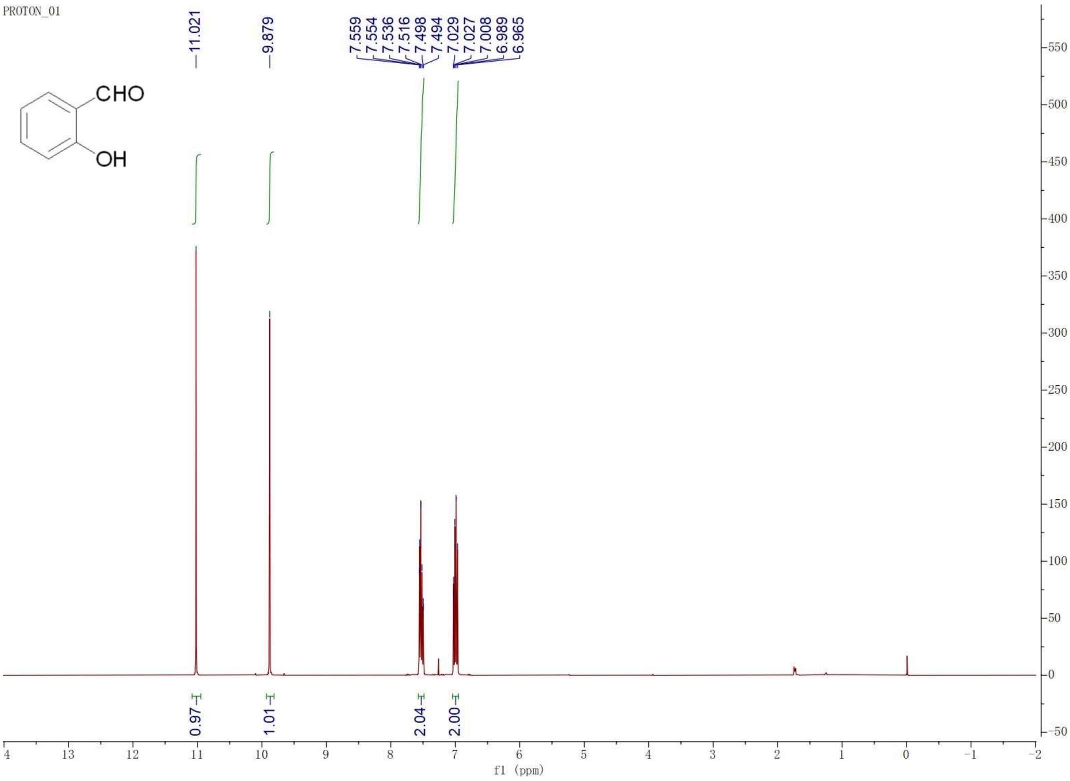

^{13}C NMR spectra of compound **2c**:

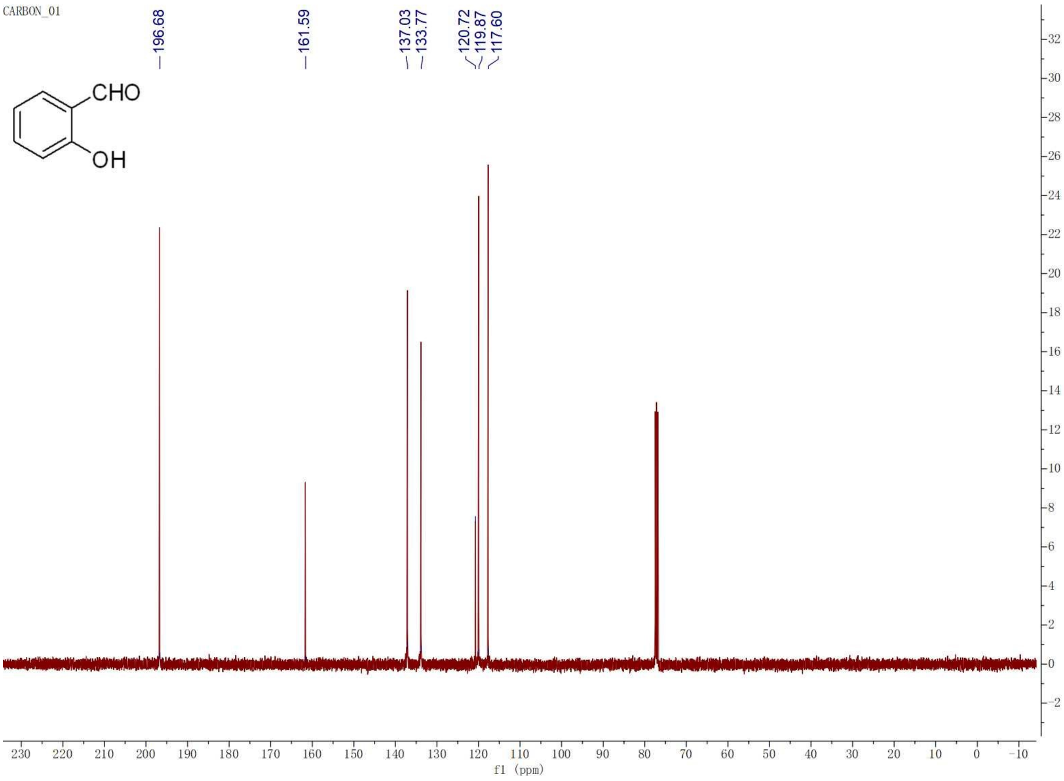

^{1}H NMR spectra of compound **2d**:

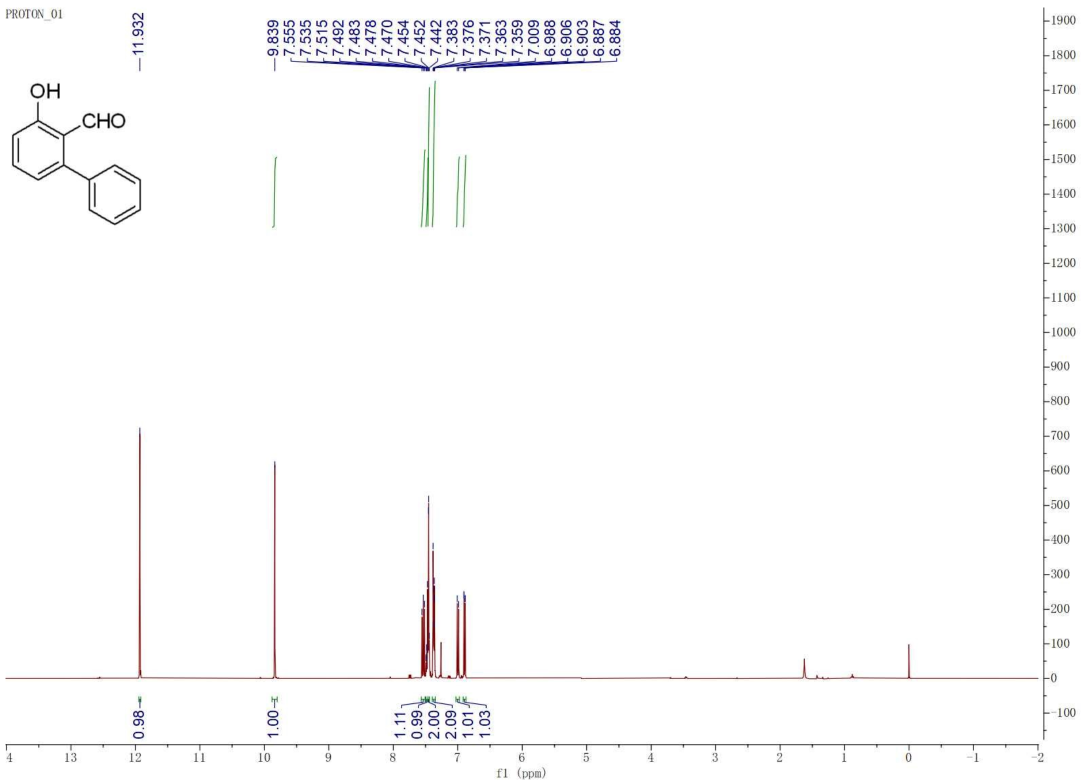

^{13}C NMR spectra of compound **2d**:

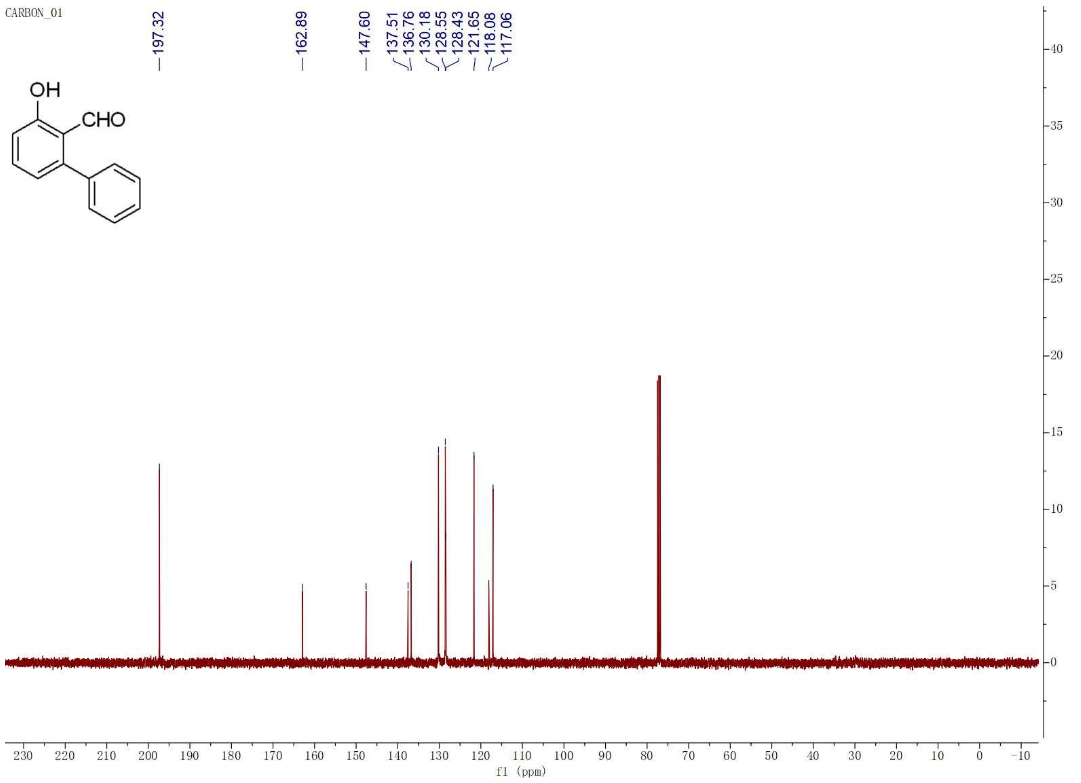

^{1}H NMR spectra of compound **2e**:

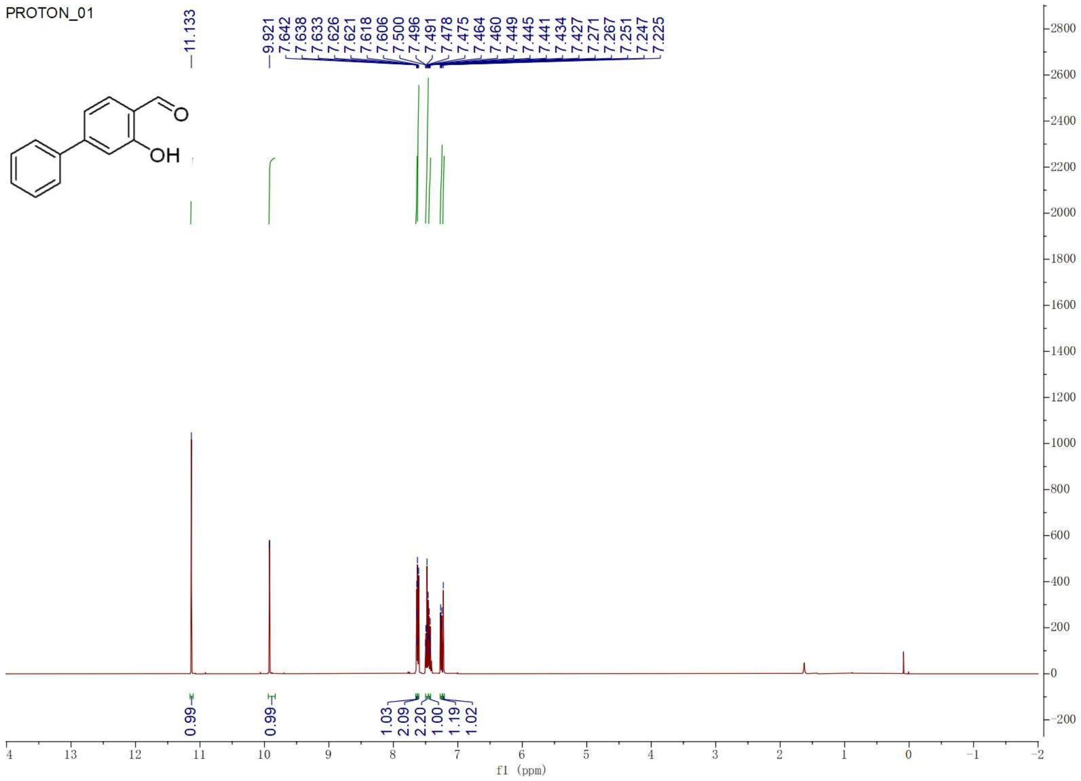

^{13}C NMR spectra of compound **2e**:

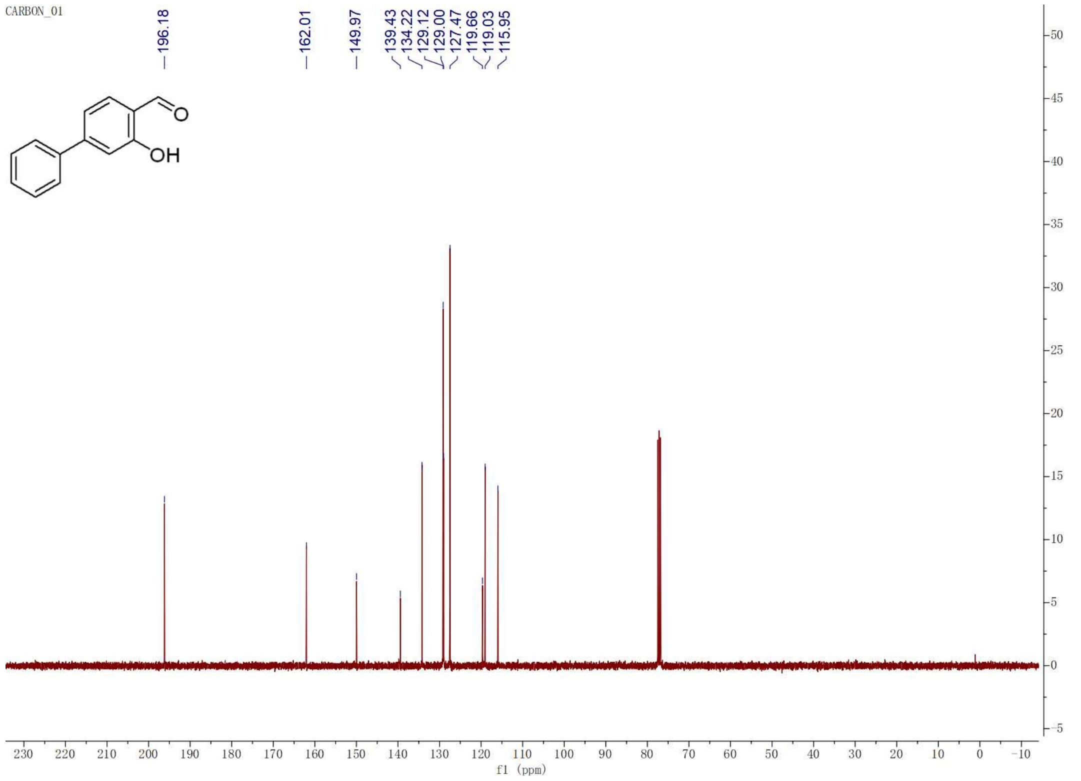

^{1}H NMR spectra of compound **2f**:

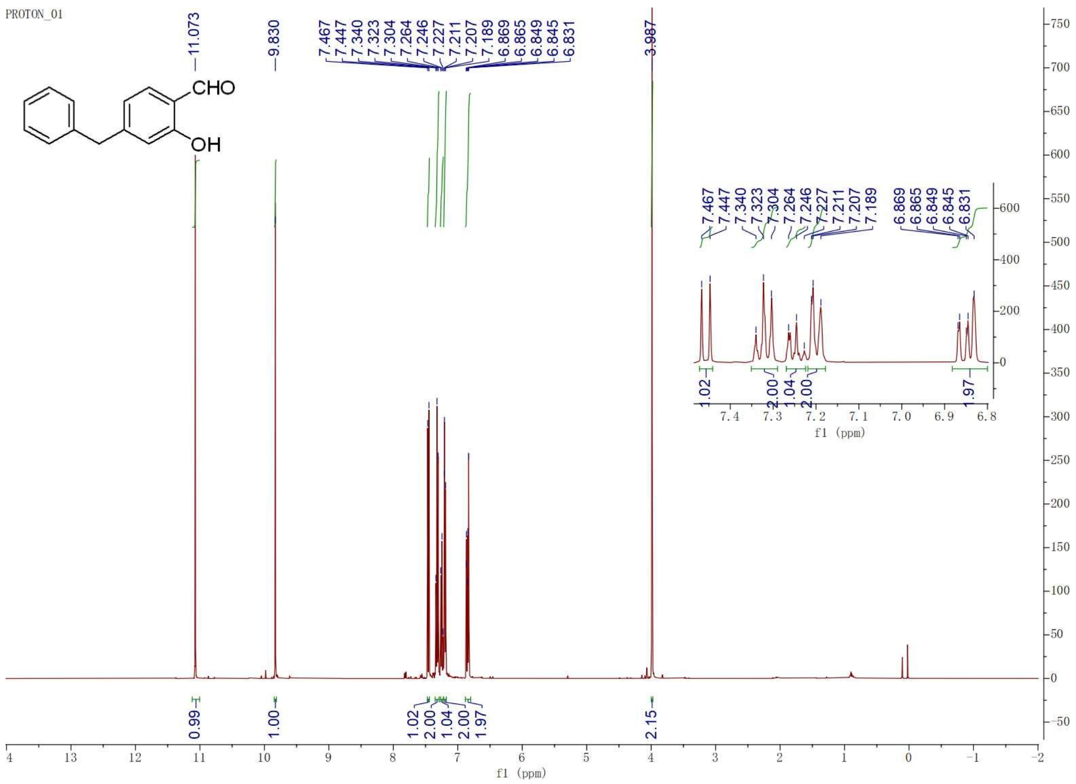

^{13}C NMR spectra of compound **2f**:

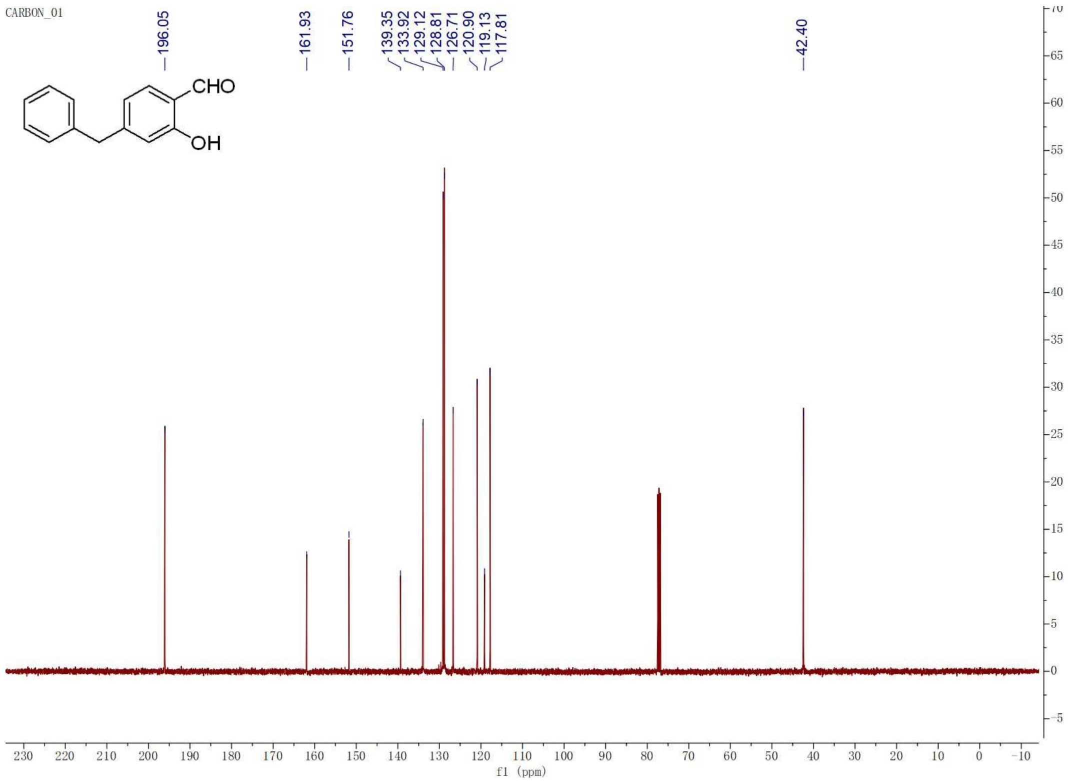

^{1}H NMR spectra of compound **2g**:

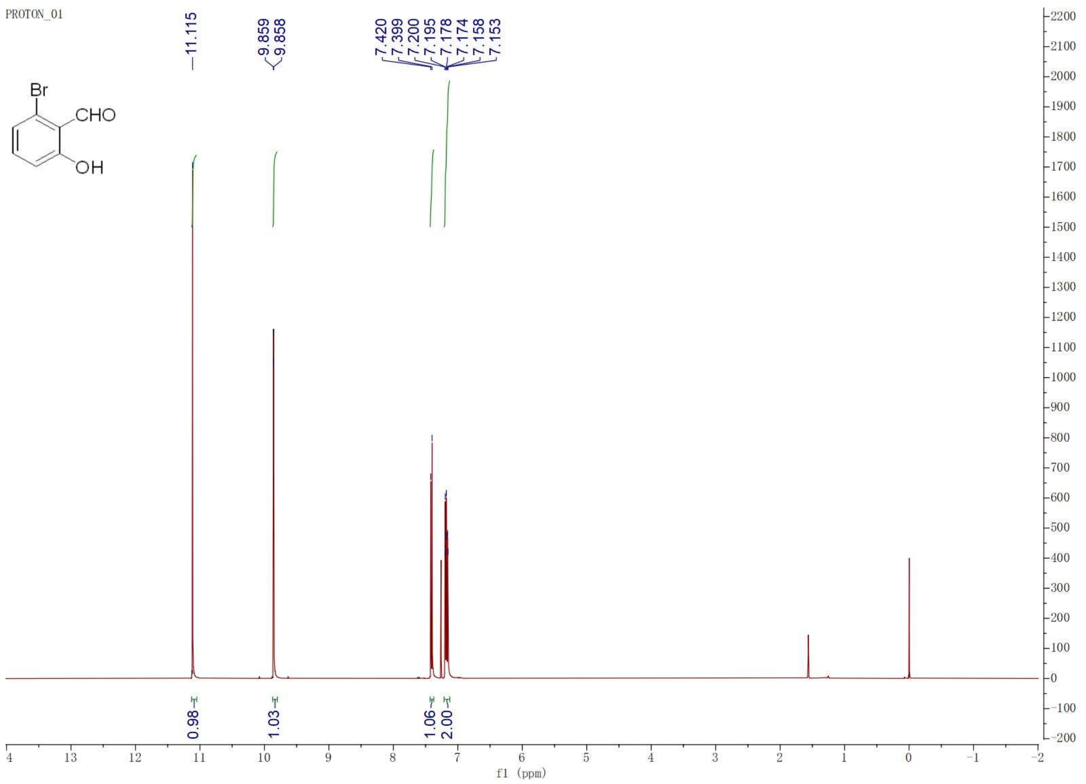

^{13}C NMR spectra of compound **2g**:

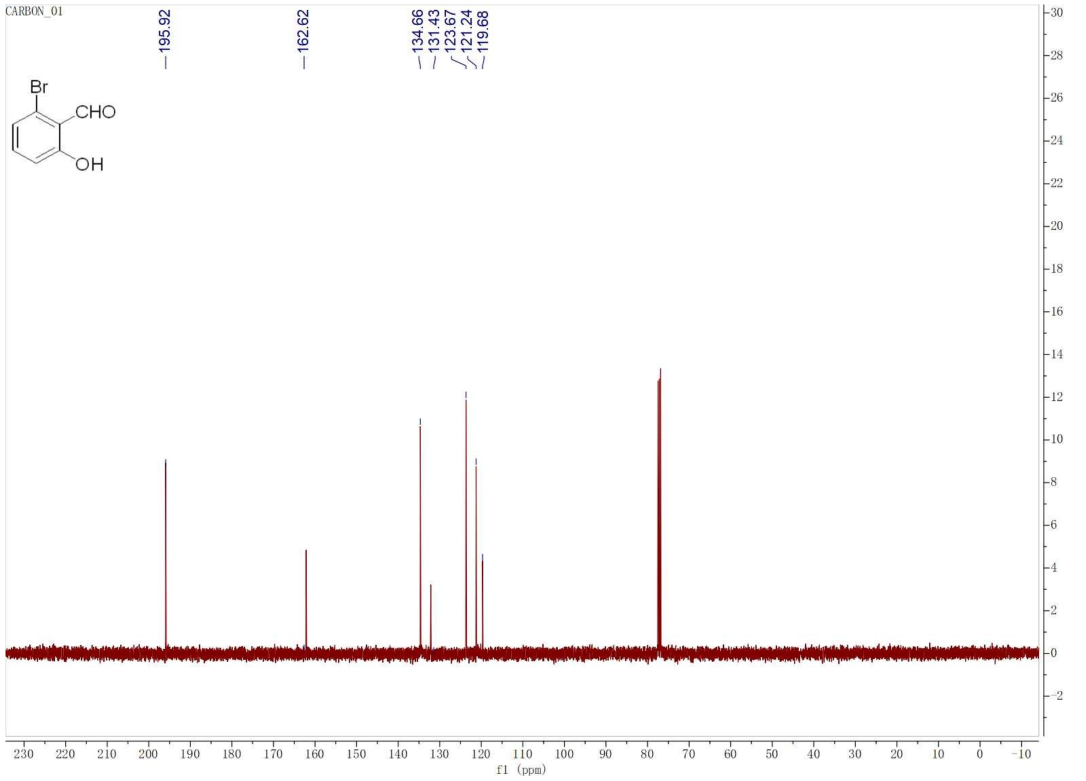

^{1}H NMR spectra of compound **2h**:

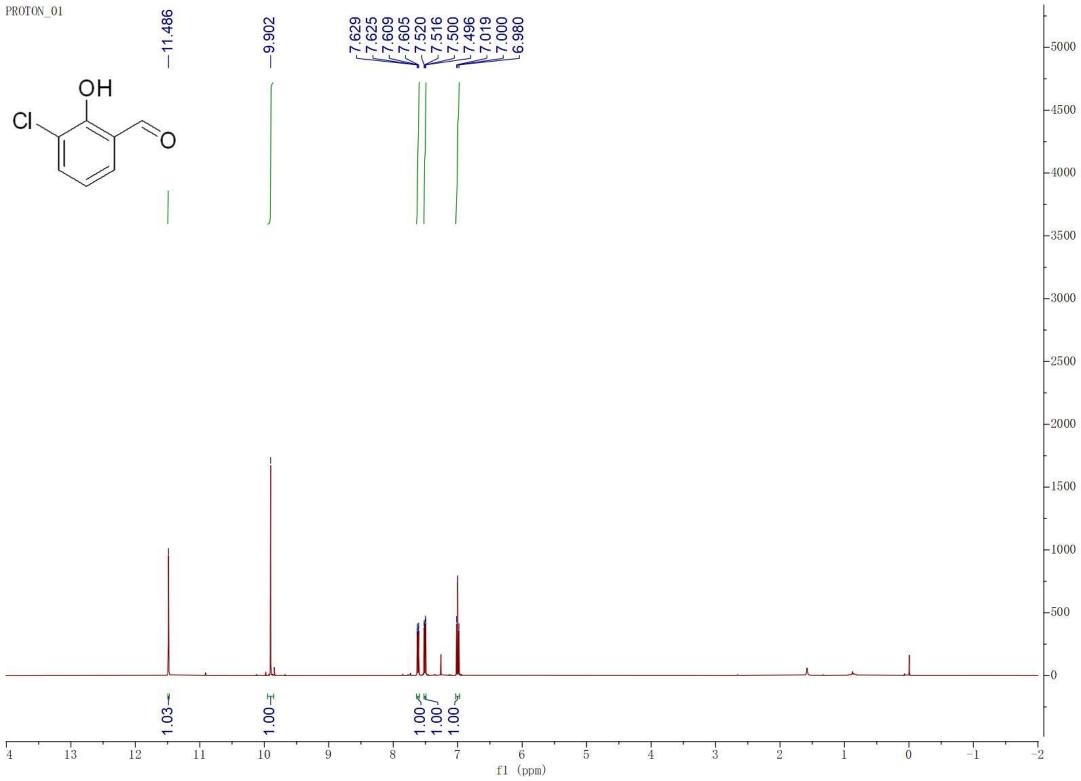

^{13}C NMR spectra of compound **2h**:

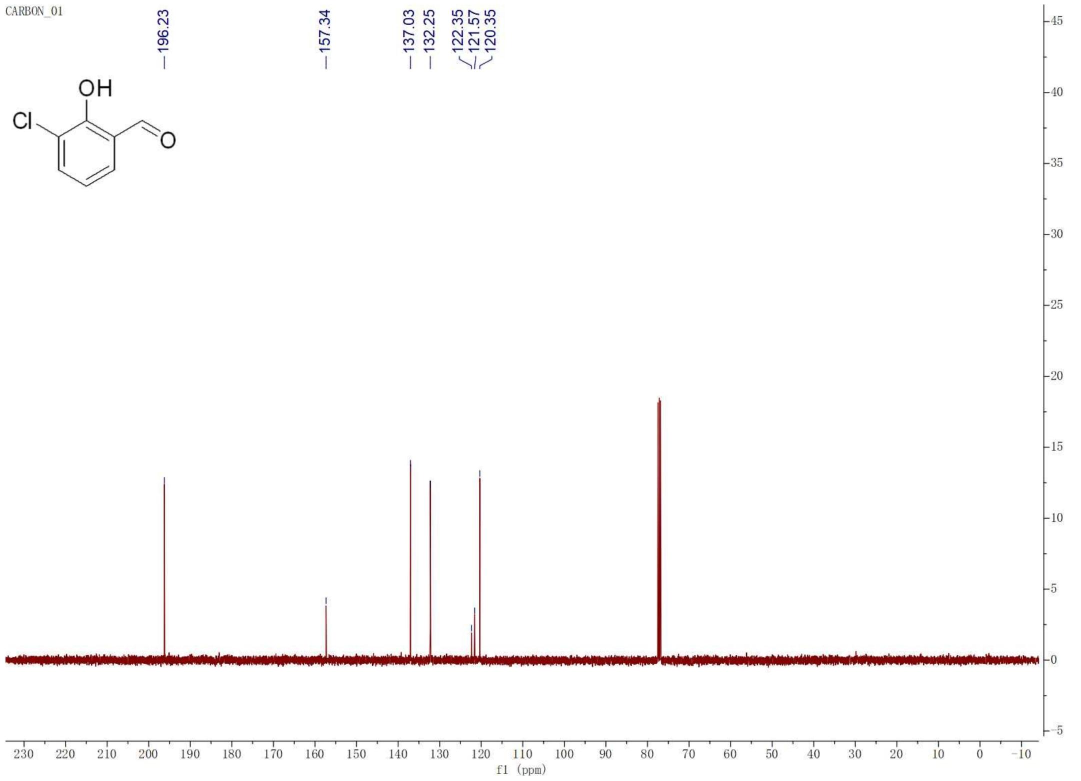

^{1}H NMR spectra of compound **2i**:

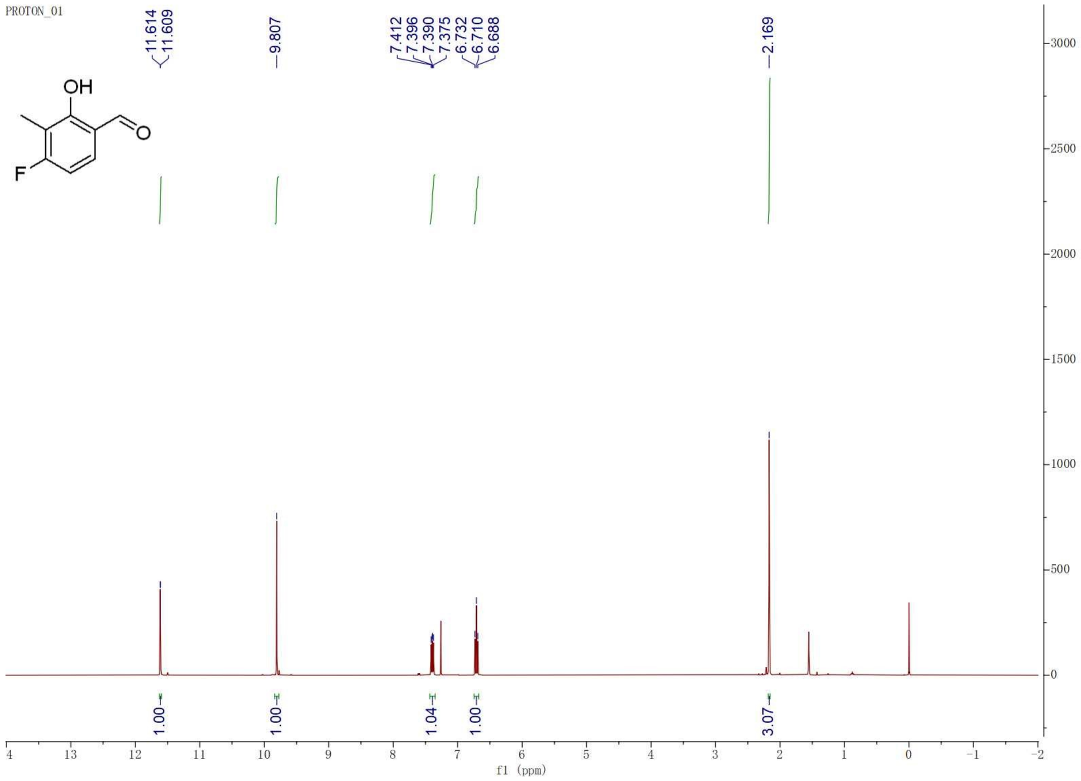

^{13}C NMR spectra of compound **2i**:

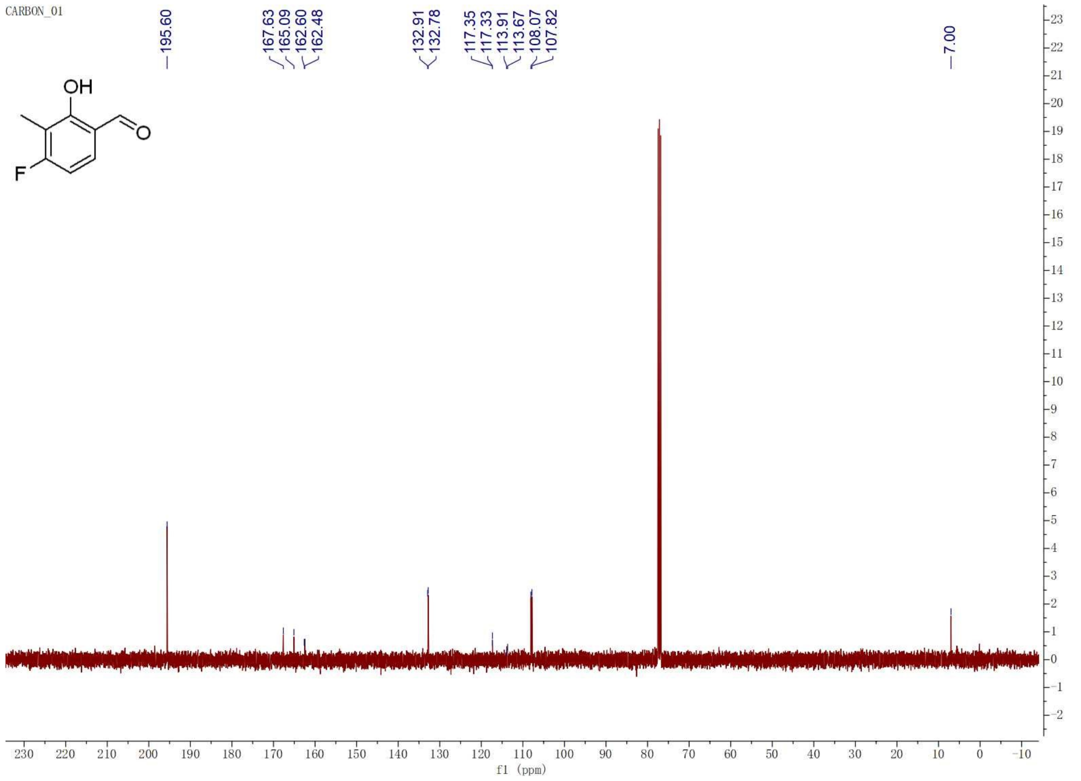

^{19}F NMR spectra of compound **2i**:

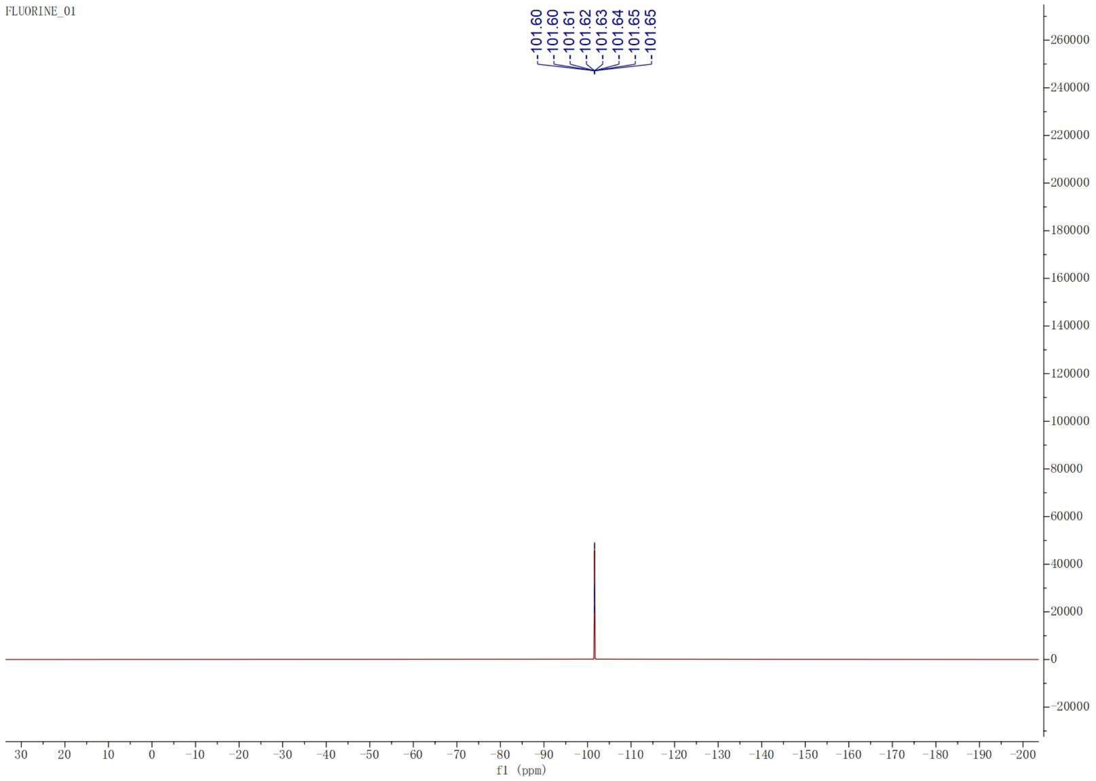

^{1}H NMR spectra of compound **2j**:

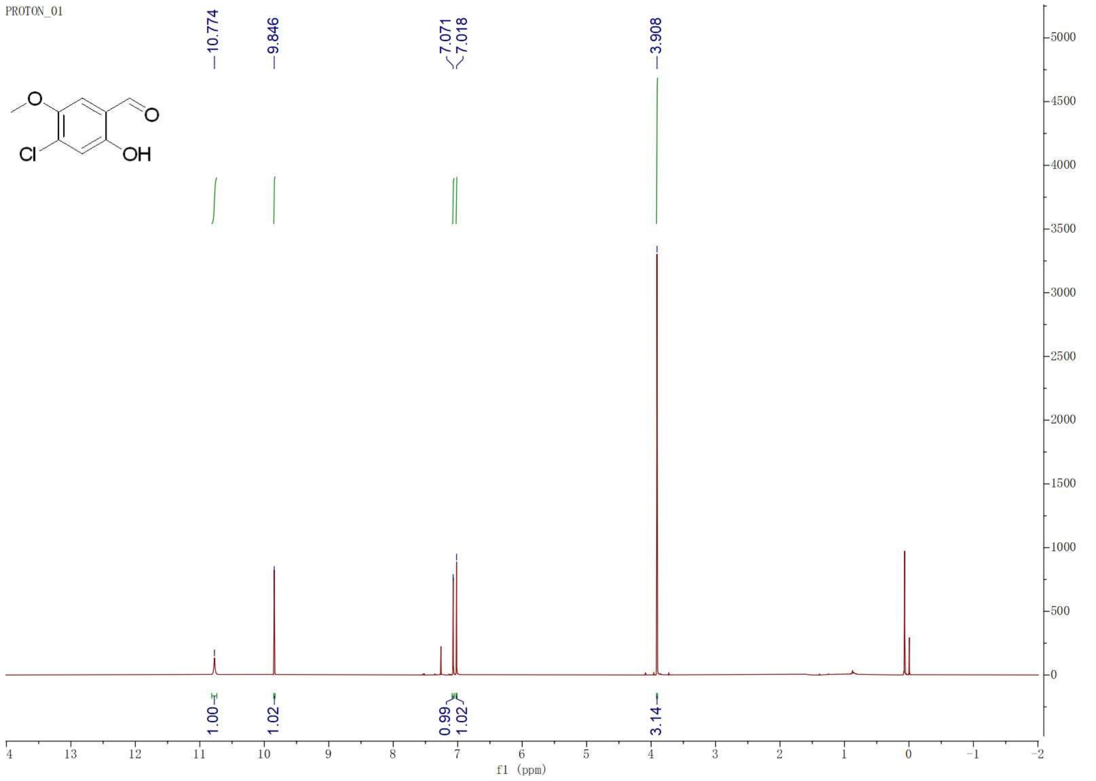

^{13}C NMR spectra of compound **2j**:

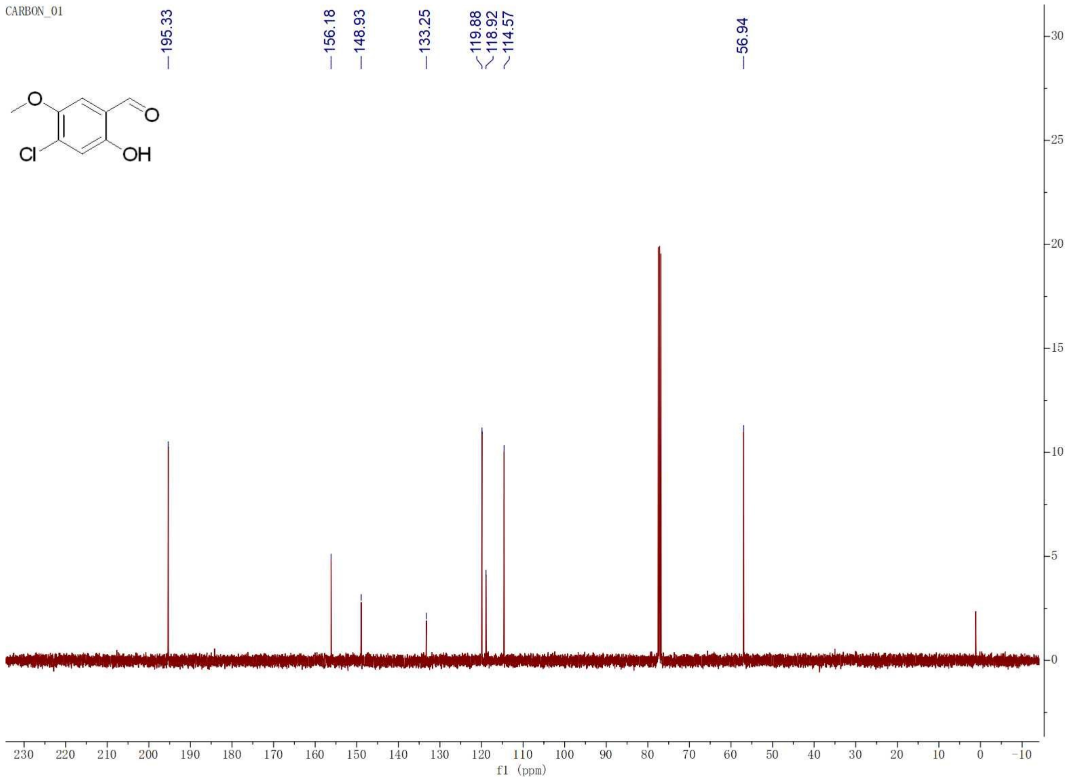

^{1}H NMR spectra of compound **2k**:

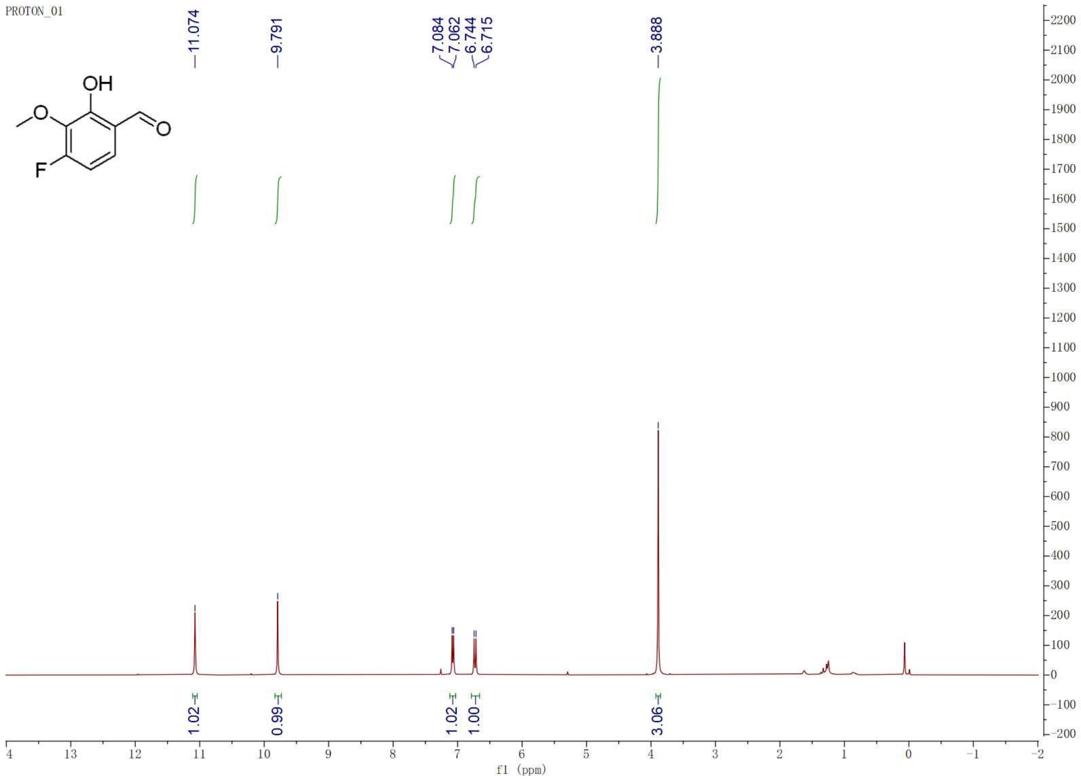

^{13}C NMR spectra of compound **2k**:

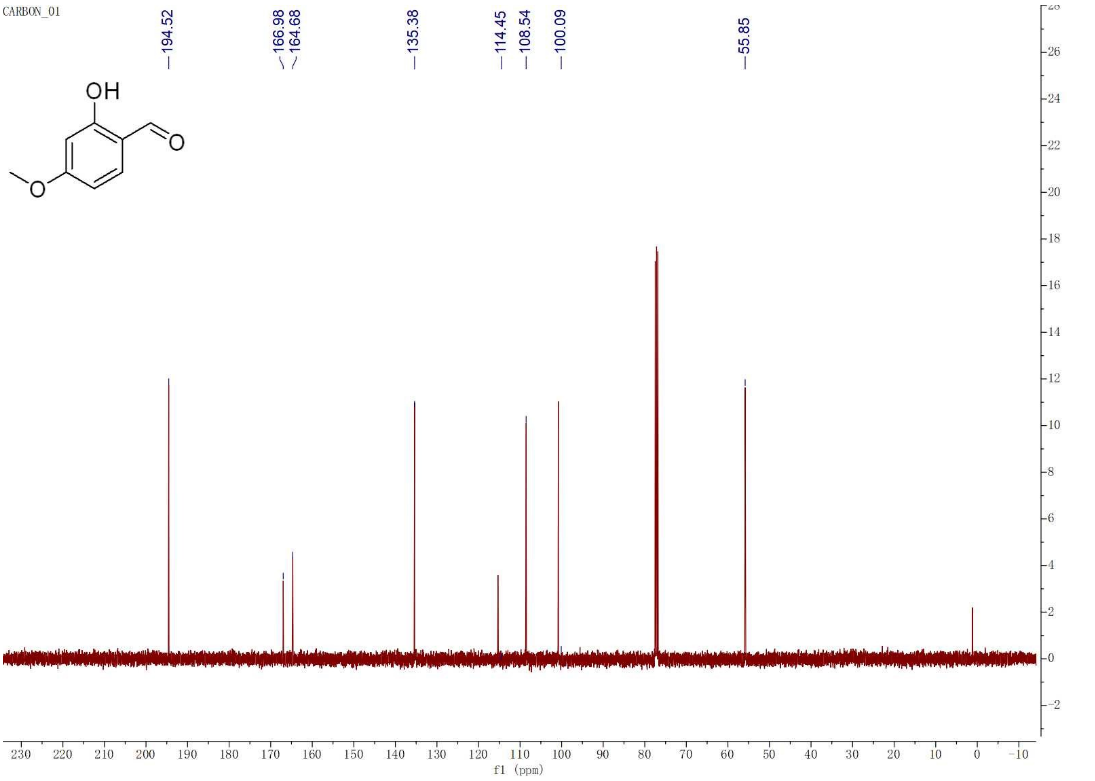

^{19}F NMR spectra of compound **2k**:

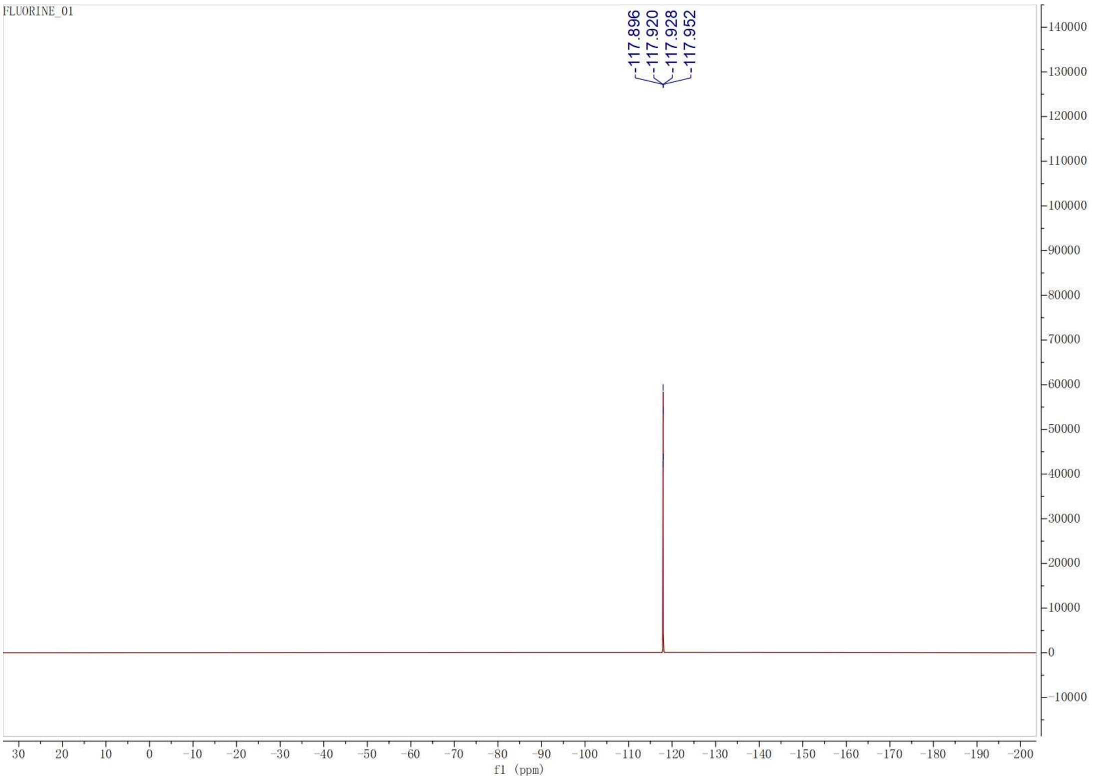

^{1}H NMR spectra of compound **2l**:

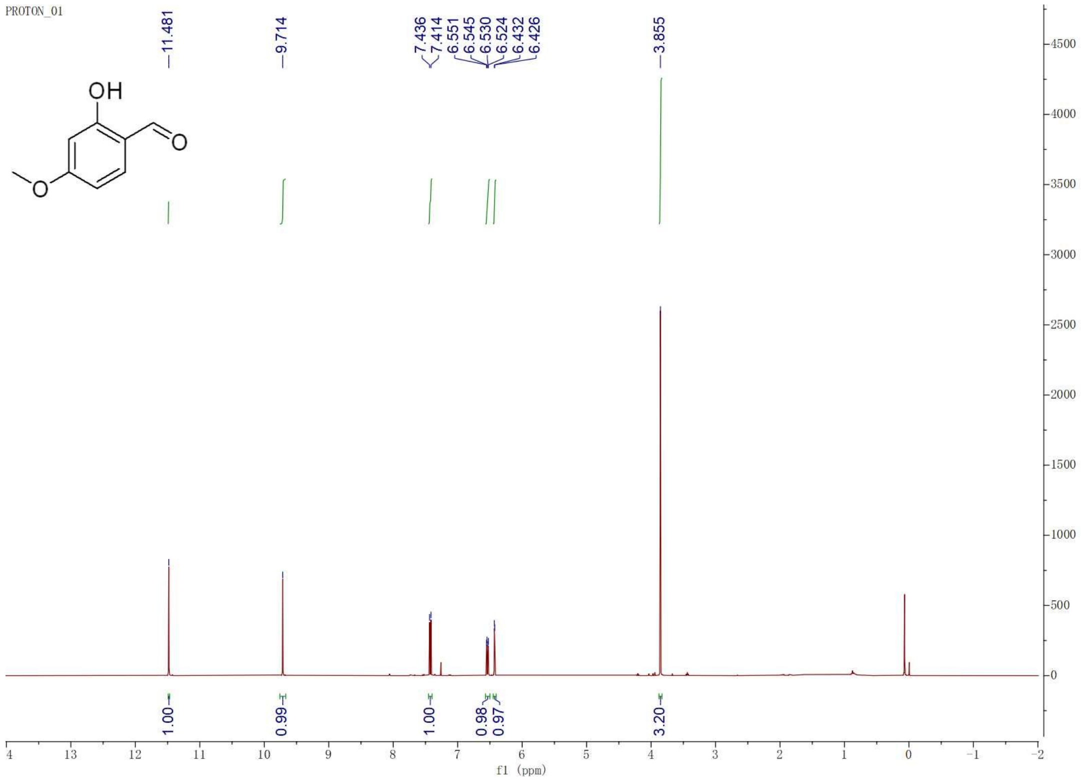

^{13}C NMR spectra of compound **2l**:

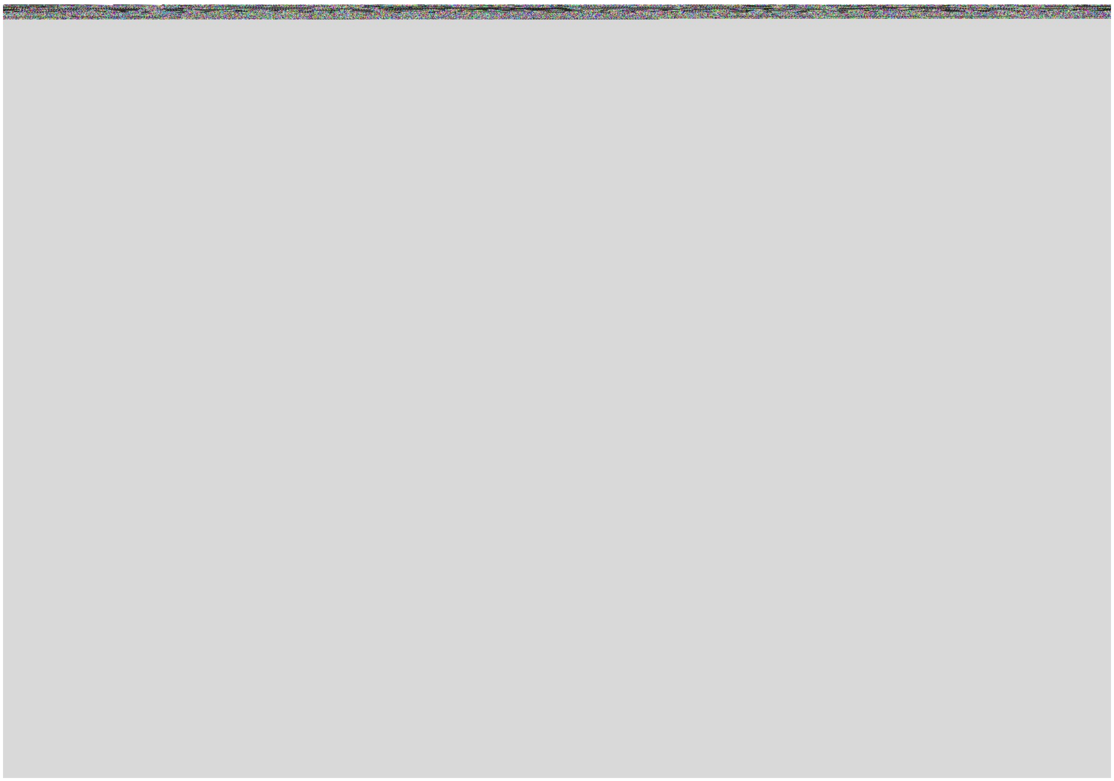

^{1}H NMR spectra of compound **2m**:

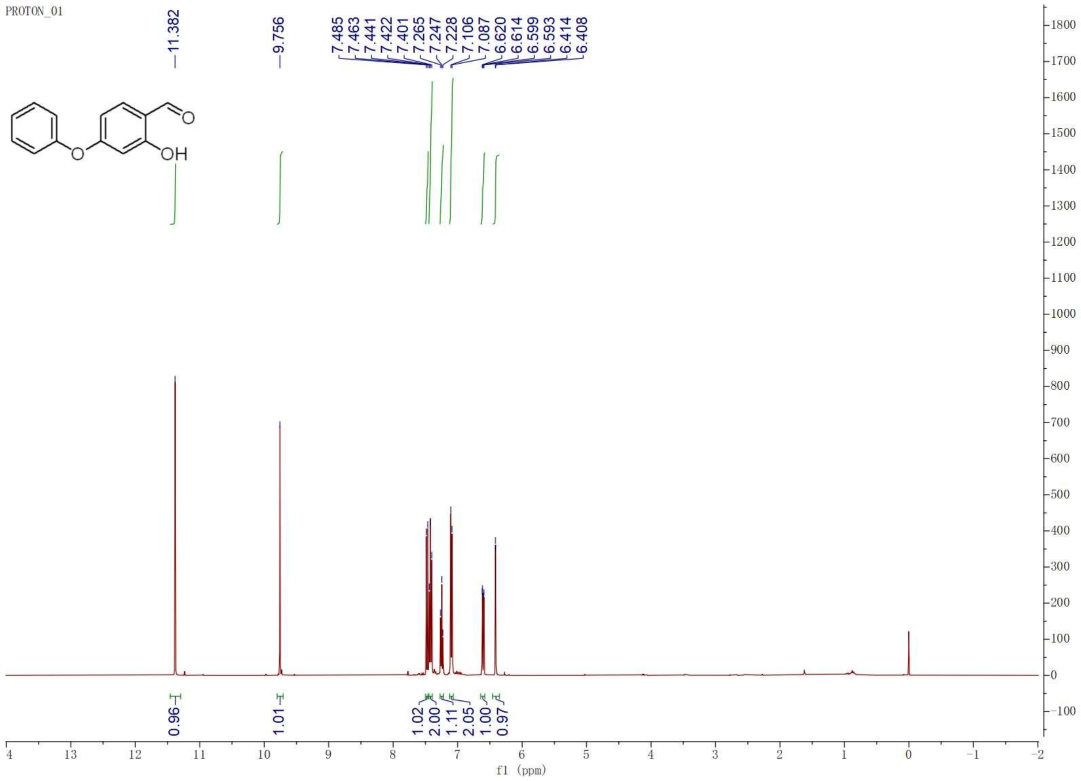

^{13}C NMR spectra of compound **2m**:

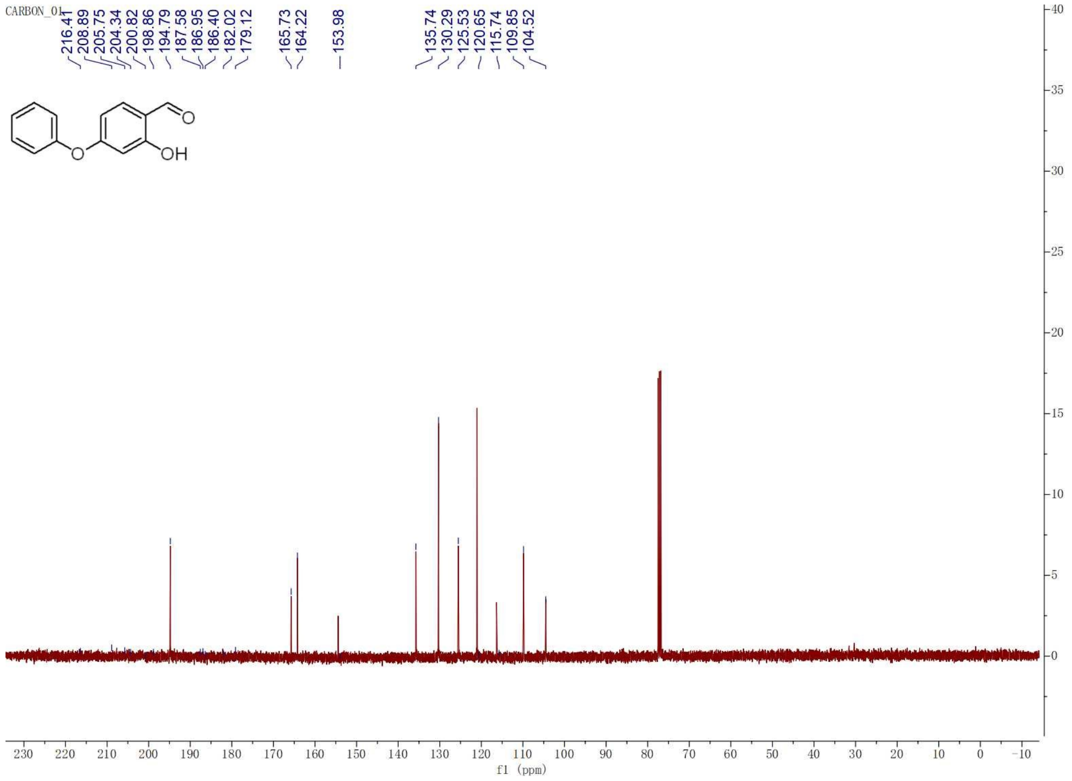

^{1}H NMR spectra of compound **2n**:

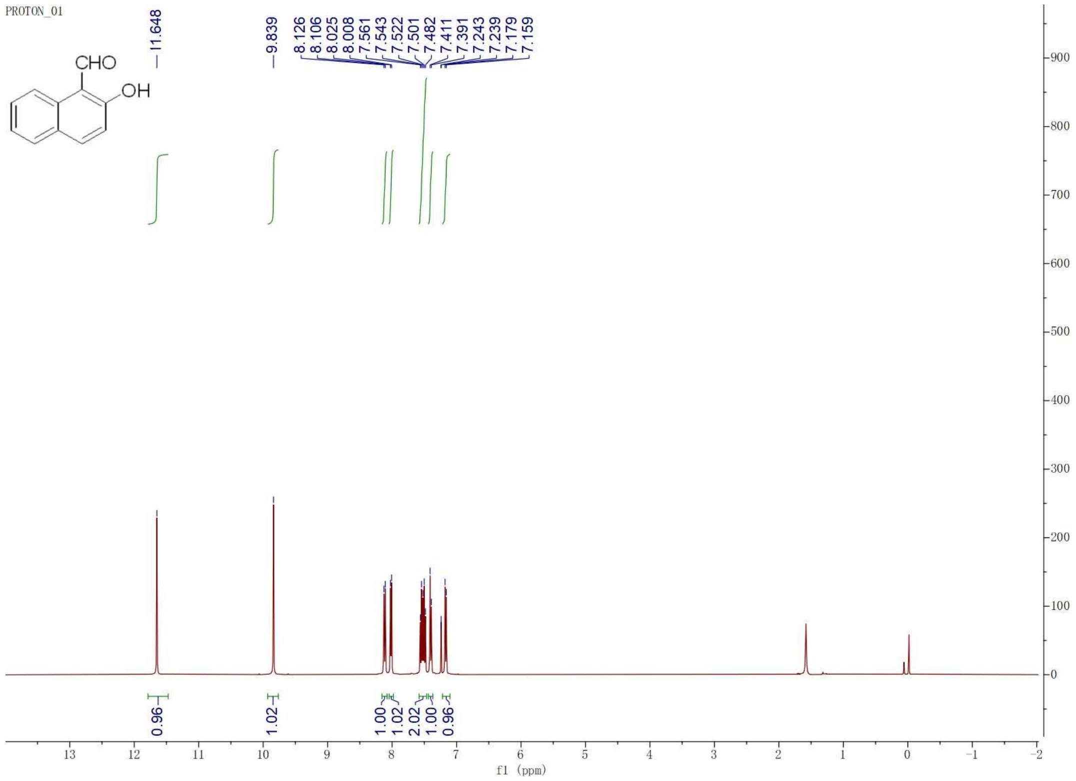

^{13}C NMR spectra of compound **2n**:

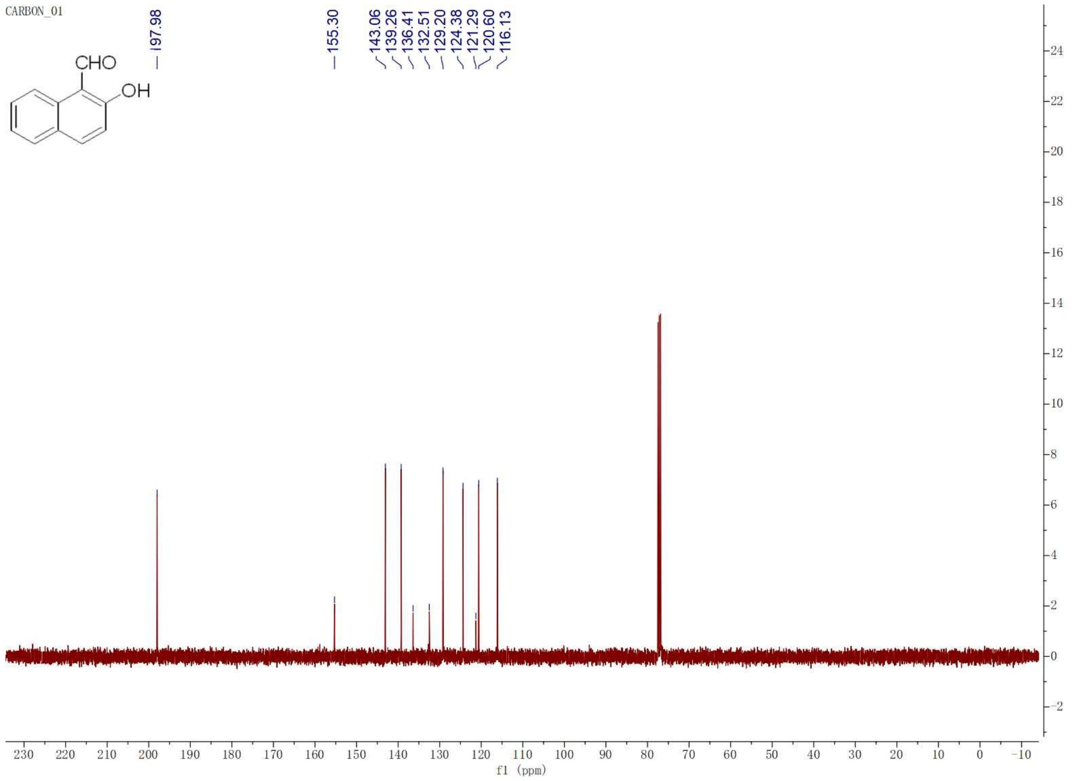

^{1}H NMR spectra of compound **2o**:

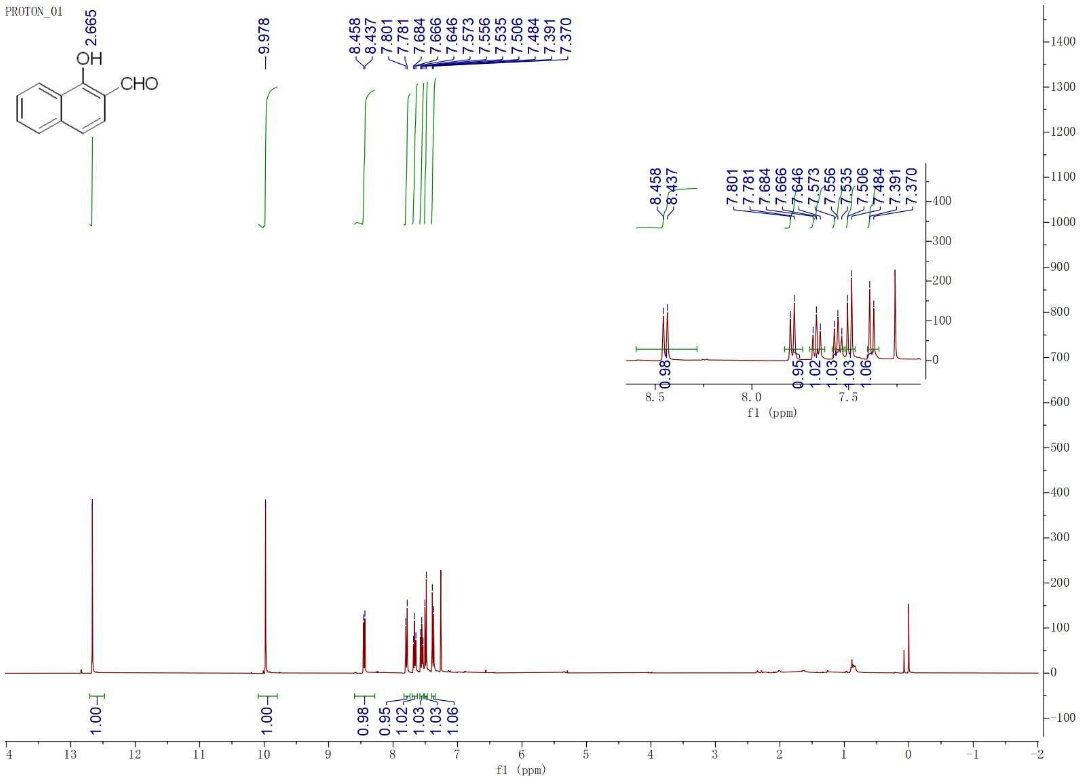

^{13}C NMR spectra of compound **2o**:

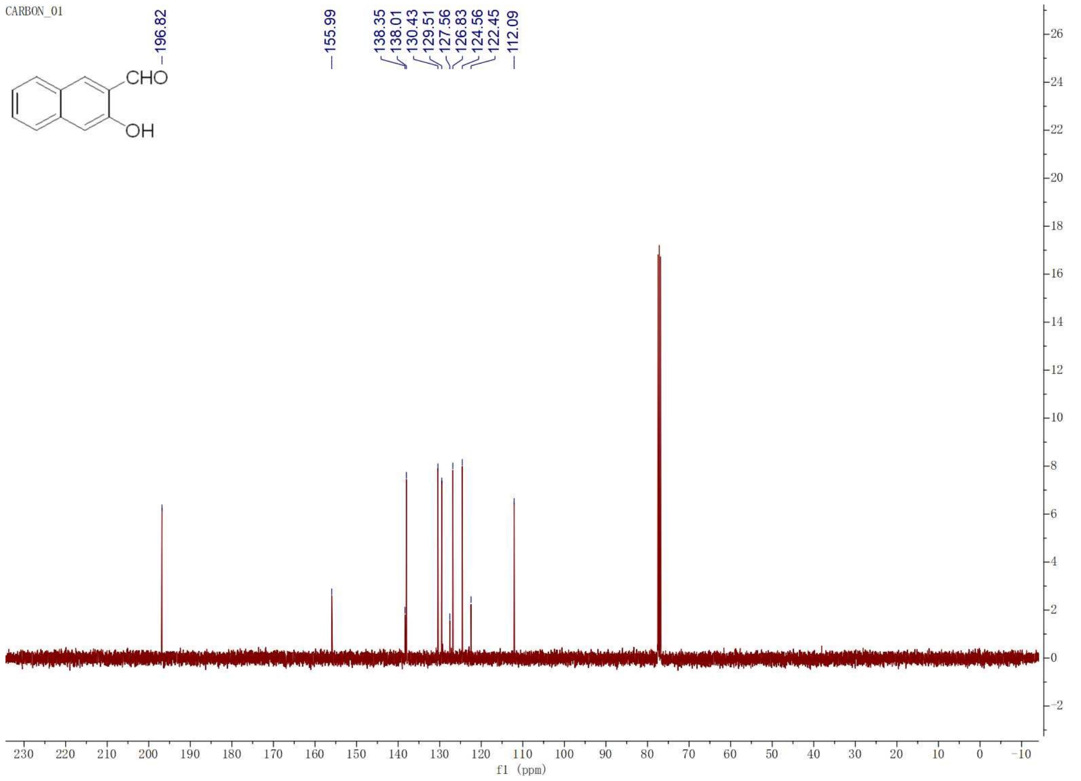

^{1}H NMR spectra of compound **2o'**:

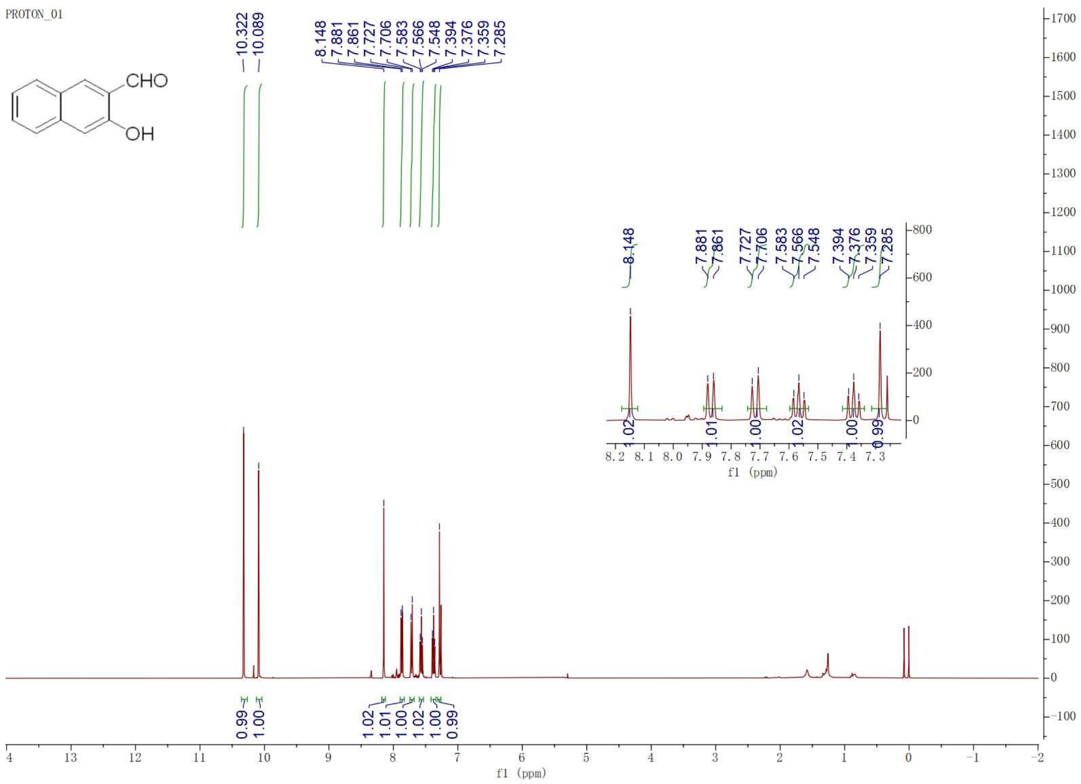

^{13}C NMR spectra of compound **2o'**:

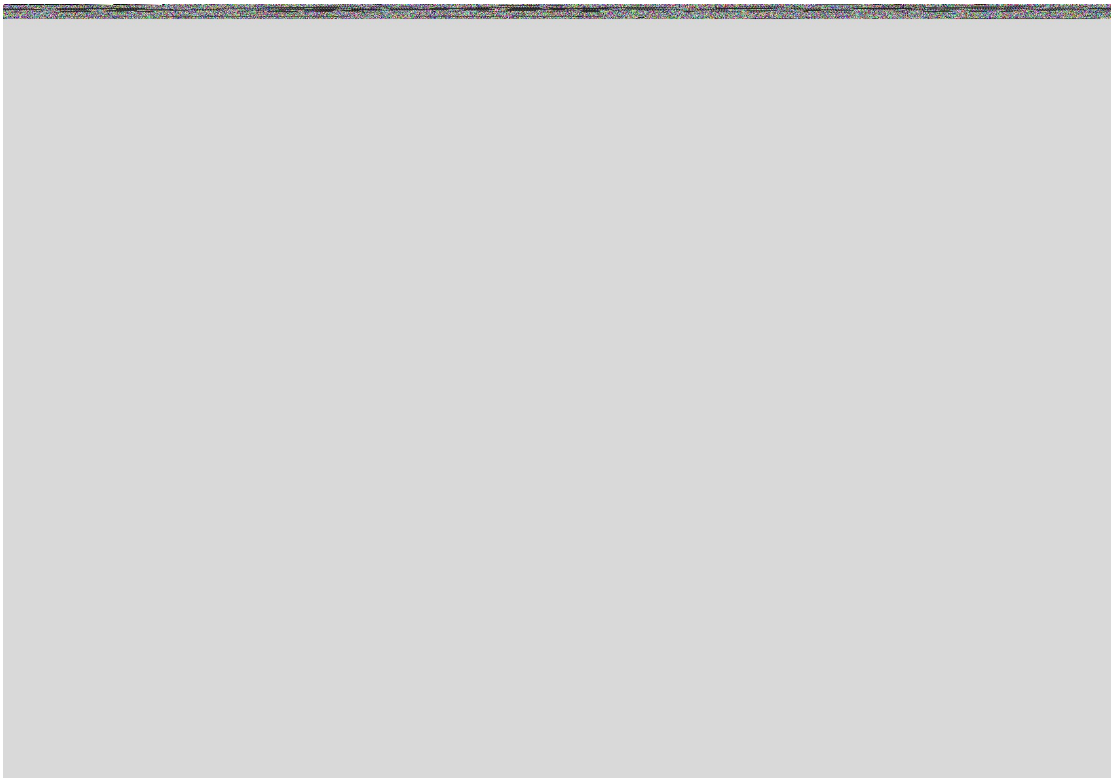

^{1}H NMR spectra of compound **2p**:

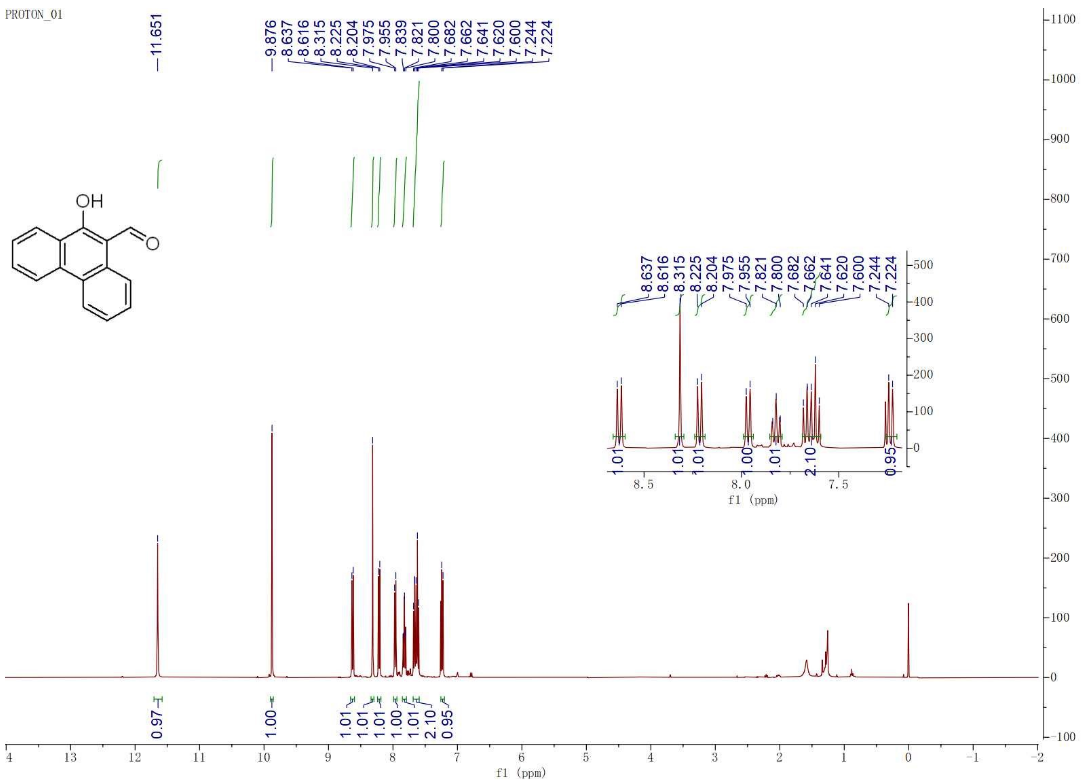

^{13}C NMR spectra of compound **2p**:

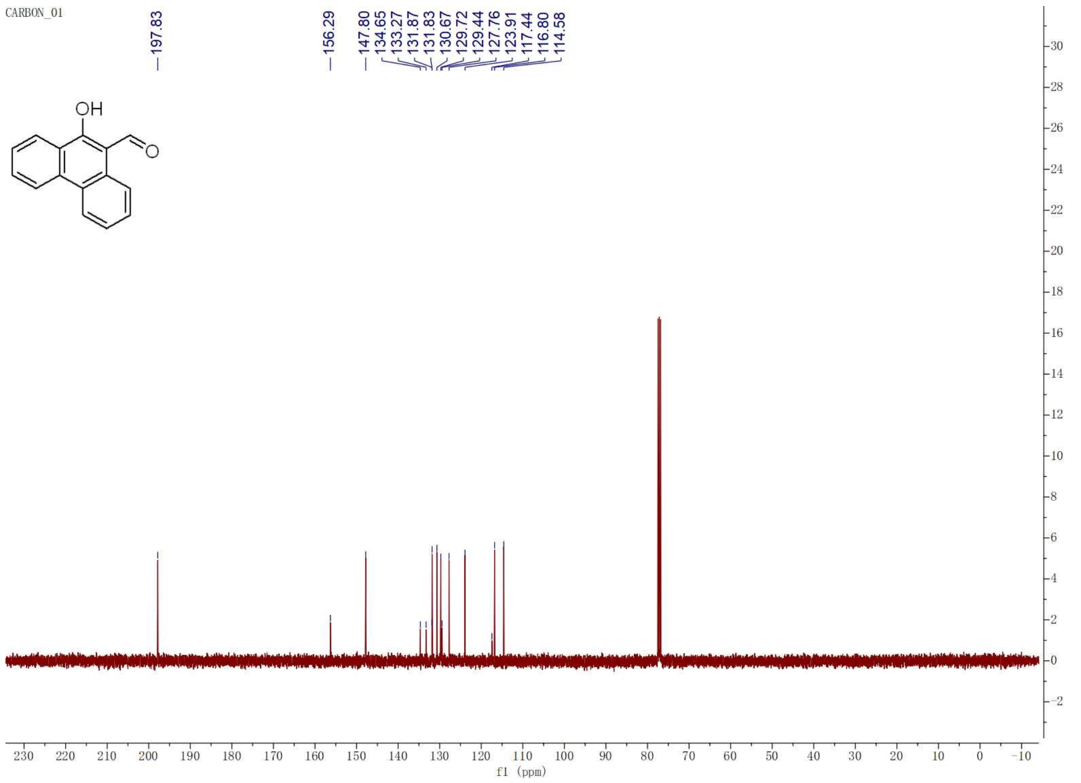

^{1}H NMR spectra of compound **2q**:

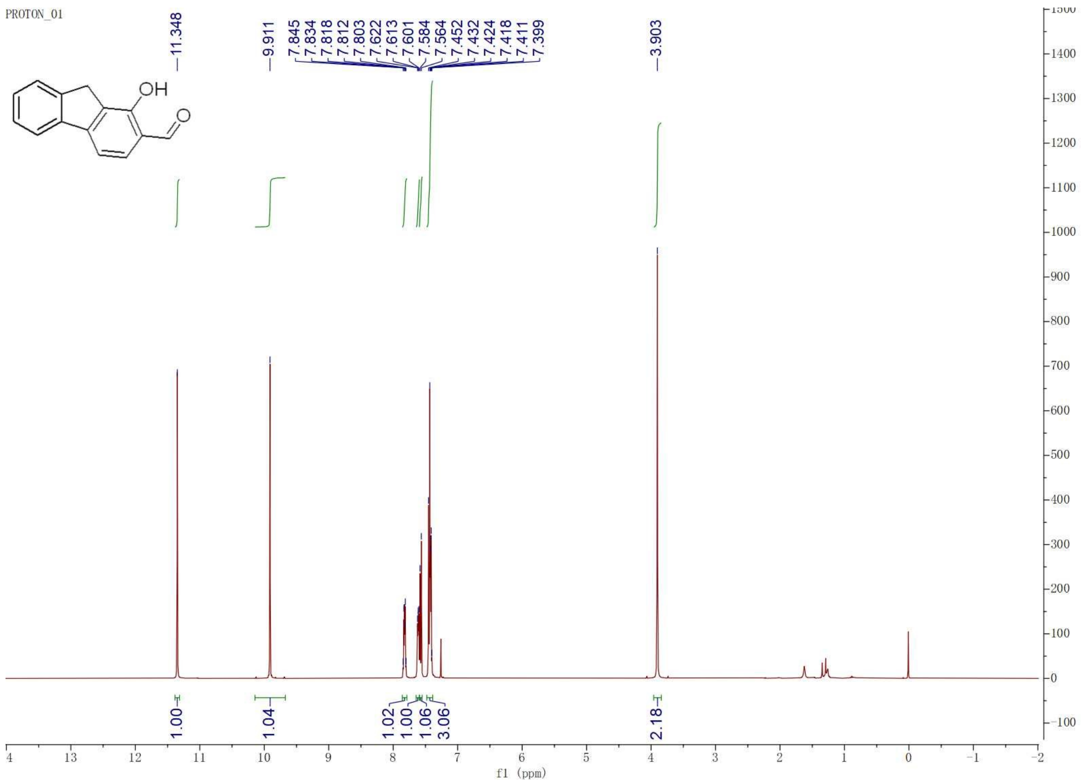

^{13}C NMR spectra of compound **2q**:

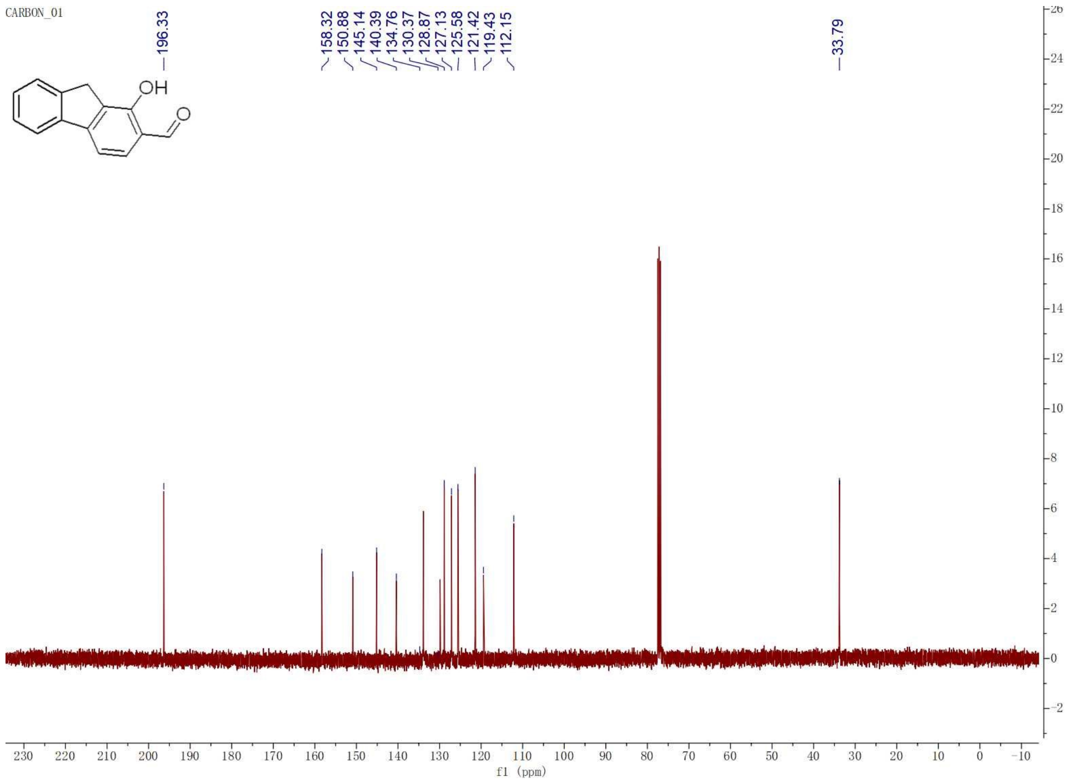

Full HMBC NMR spectra of compound **2q**:

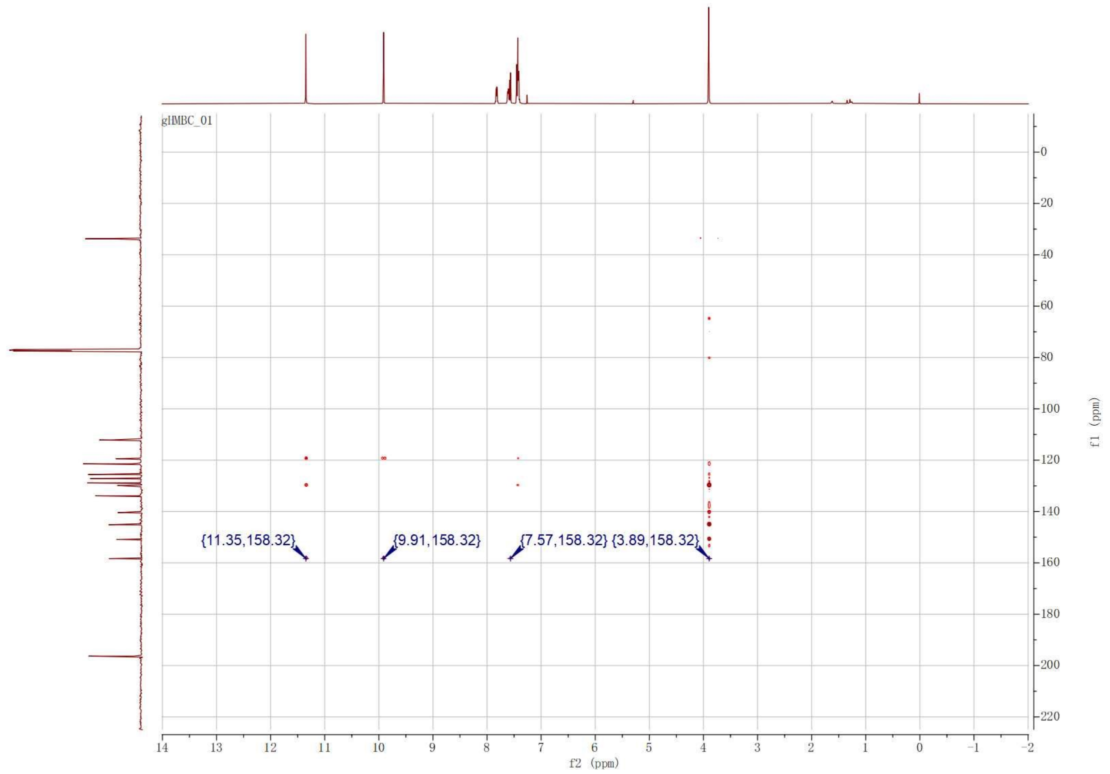

^{1}H NMR spectra of compound **2r**:

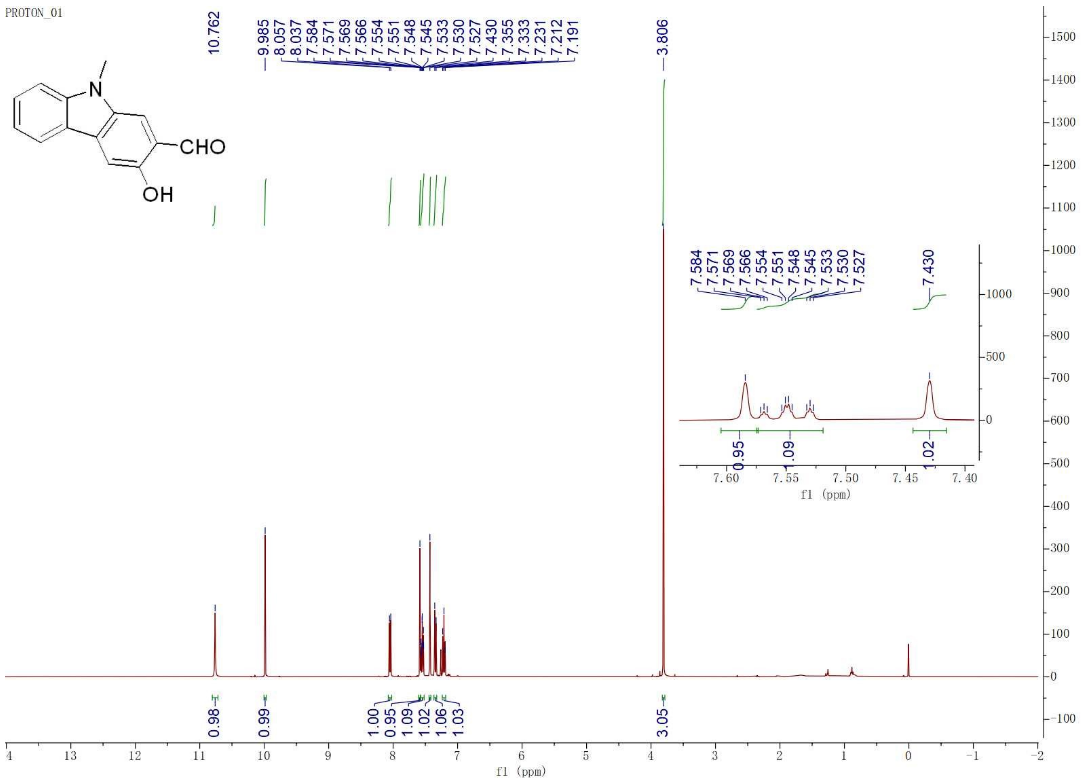

^{13}C NMR spectra of compound **2r**:

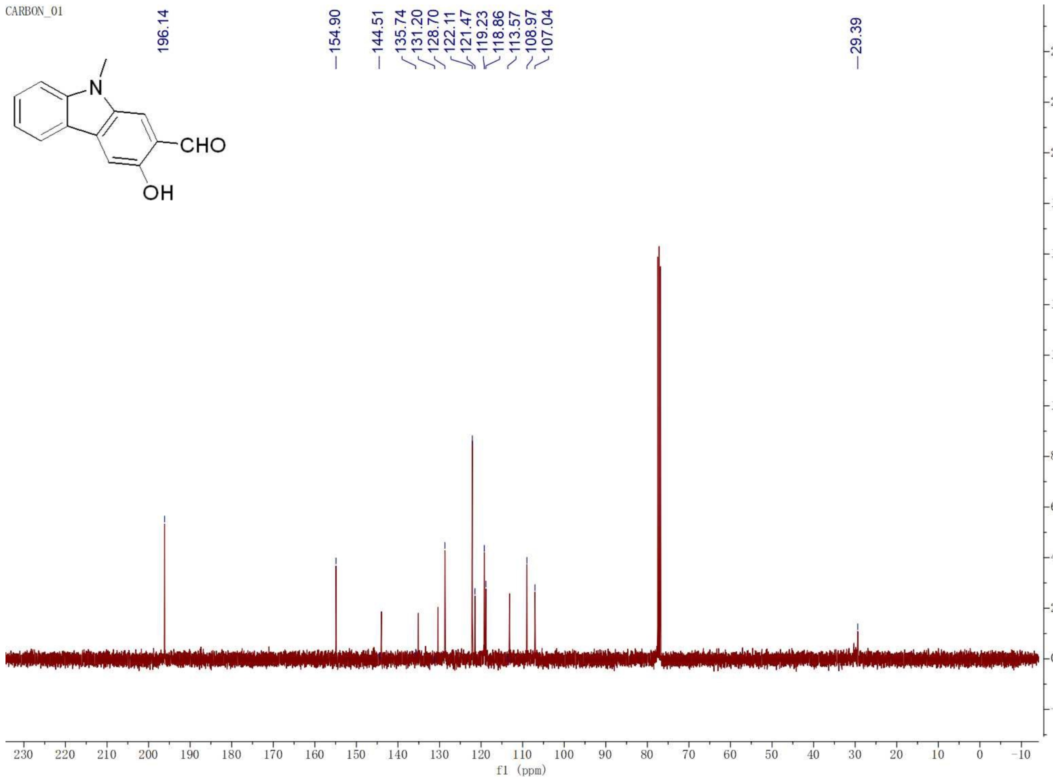

Full HMBC NMR spectra of compound **2r**:

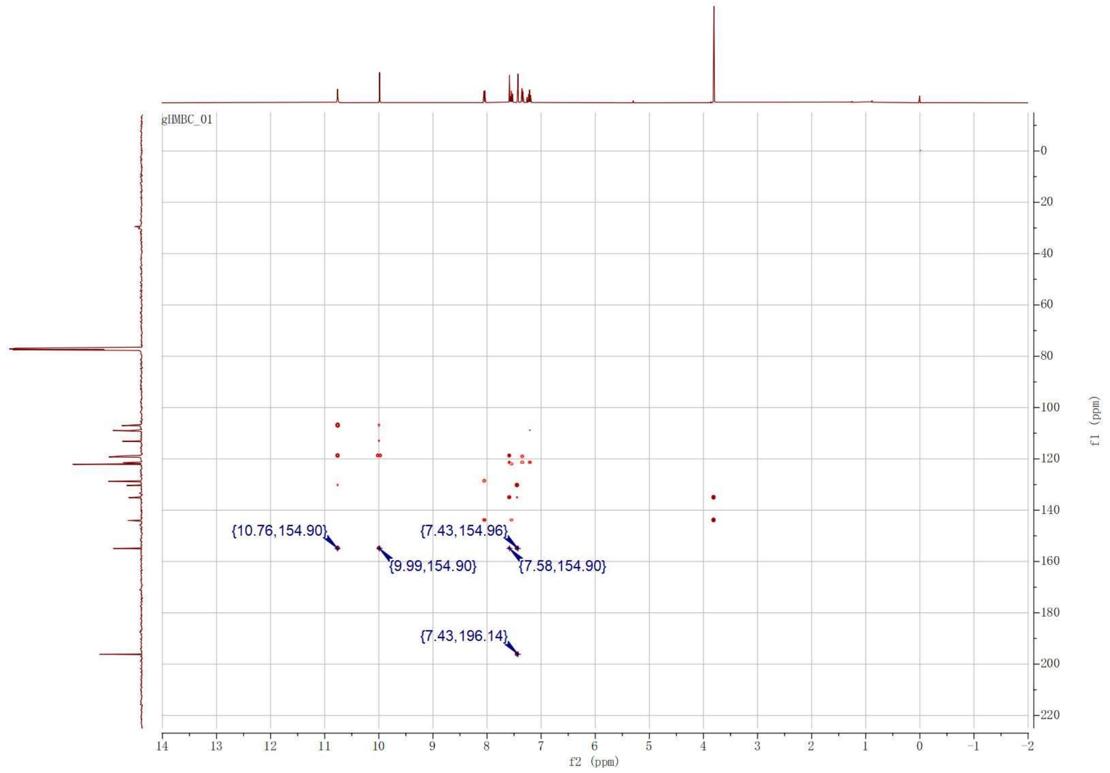

^{1}H NMR spectra of compound **2s**:

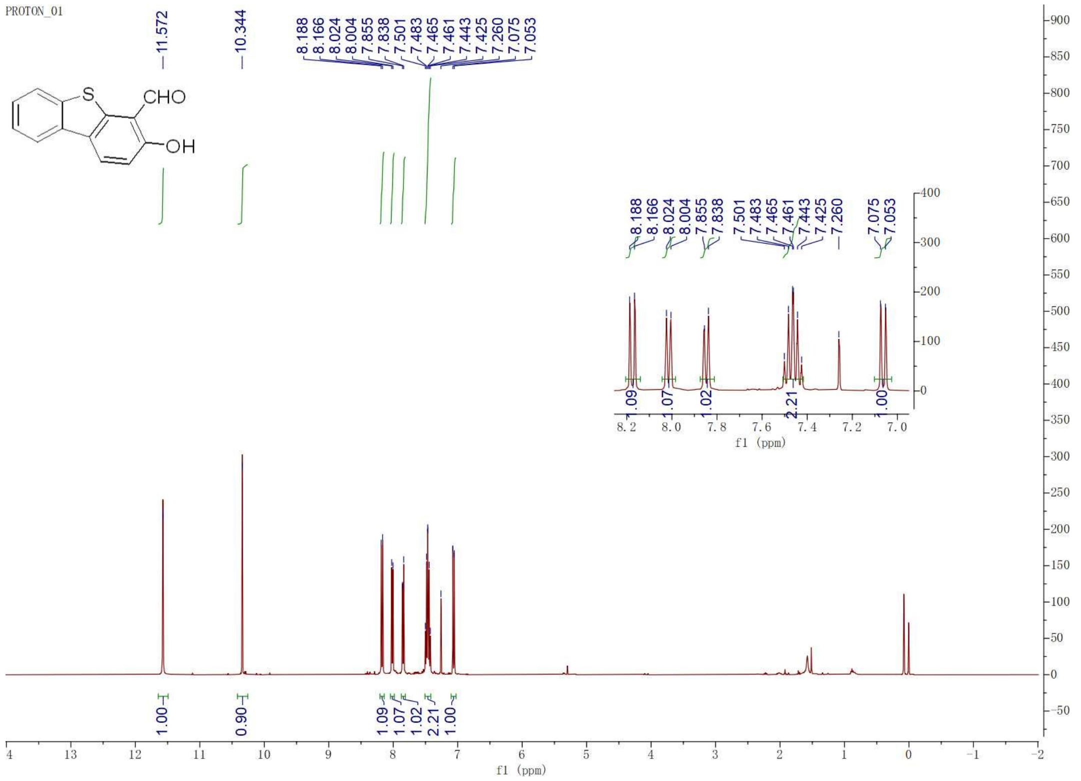

^{13}C NMR spectra of compound **2s**:

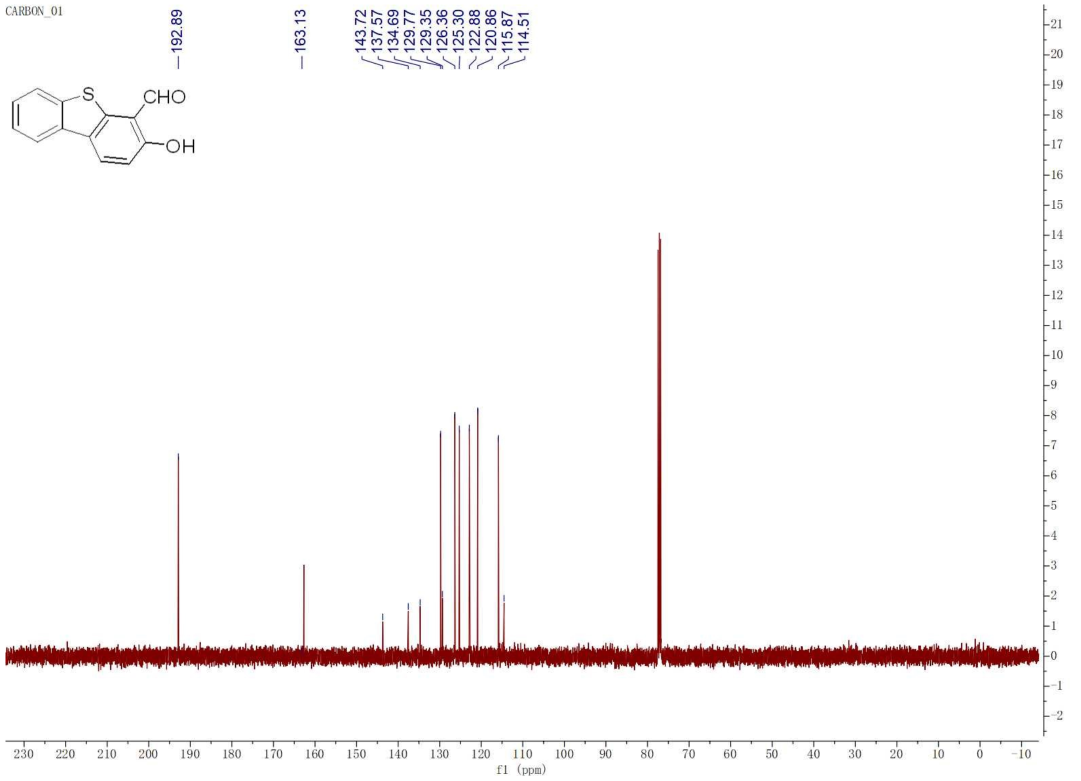

^{1}H NMR spectra of compound **3**:

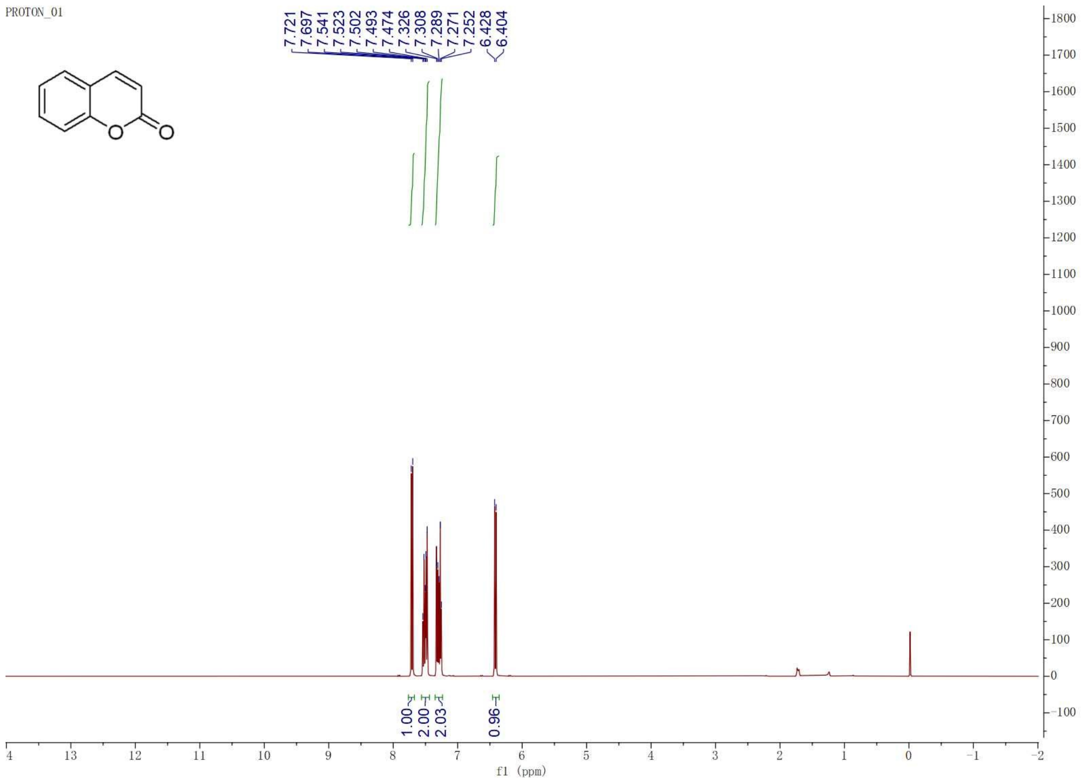

^{13}C NMR spectra of compound **3**:

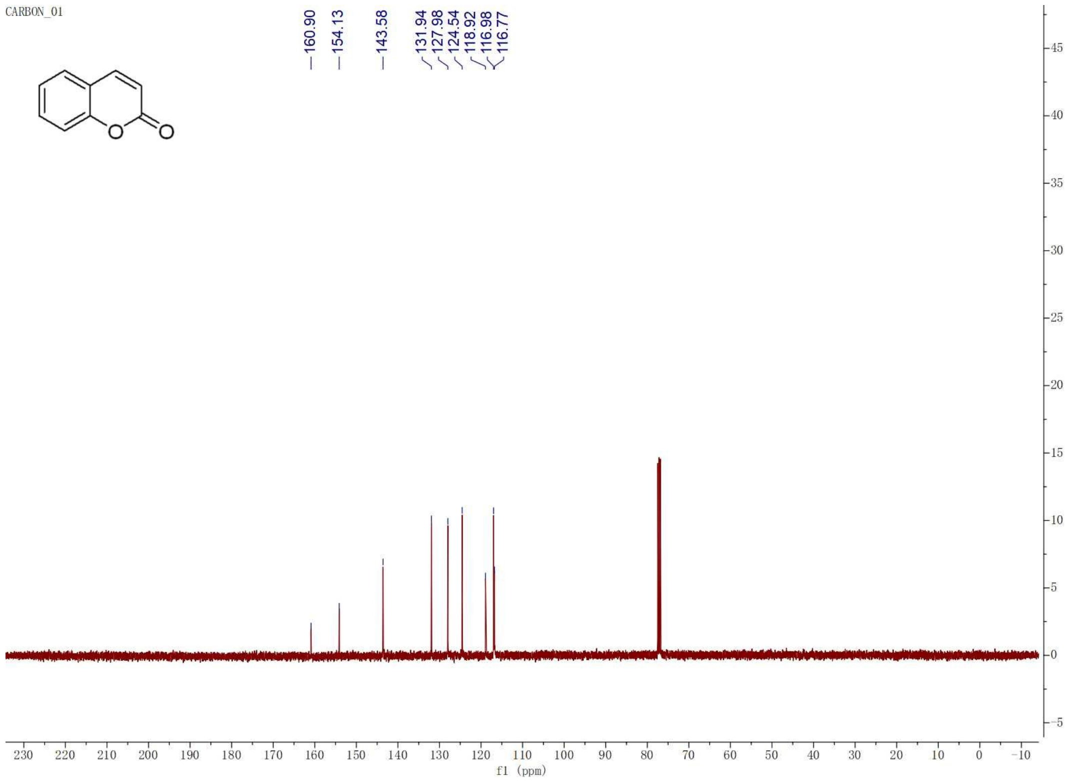

^{1}H NMR spectra of compound **4**:

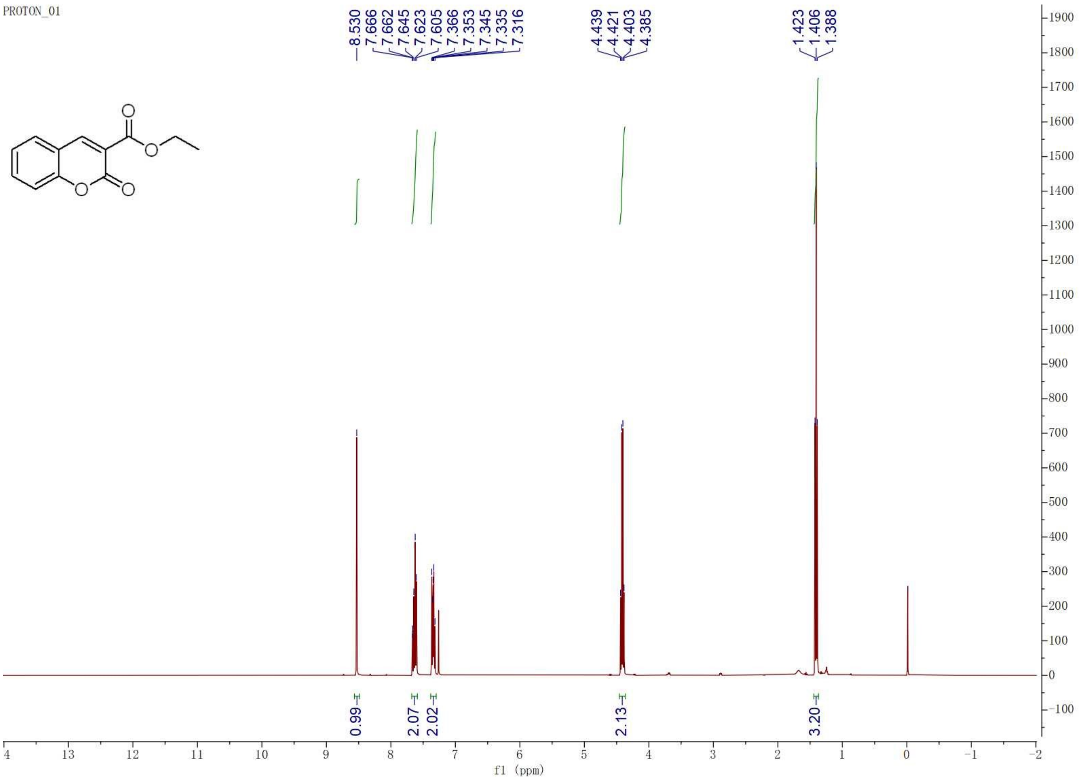

^{13}C NMR spectra of compound **4**:

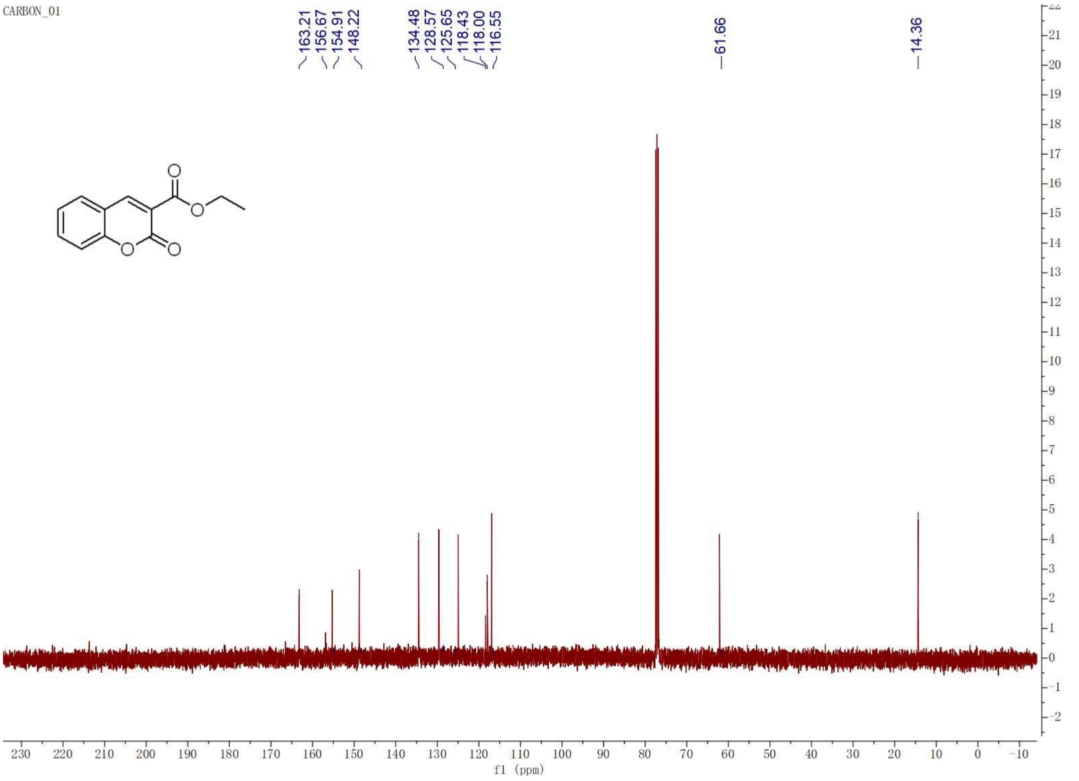

^{1}H NMR spectra of compound **5**:

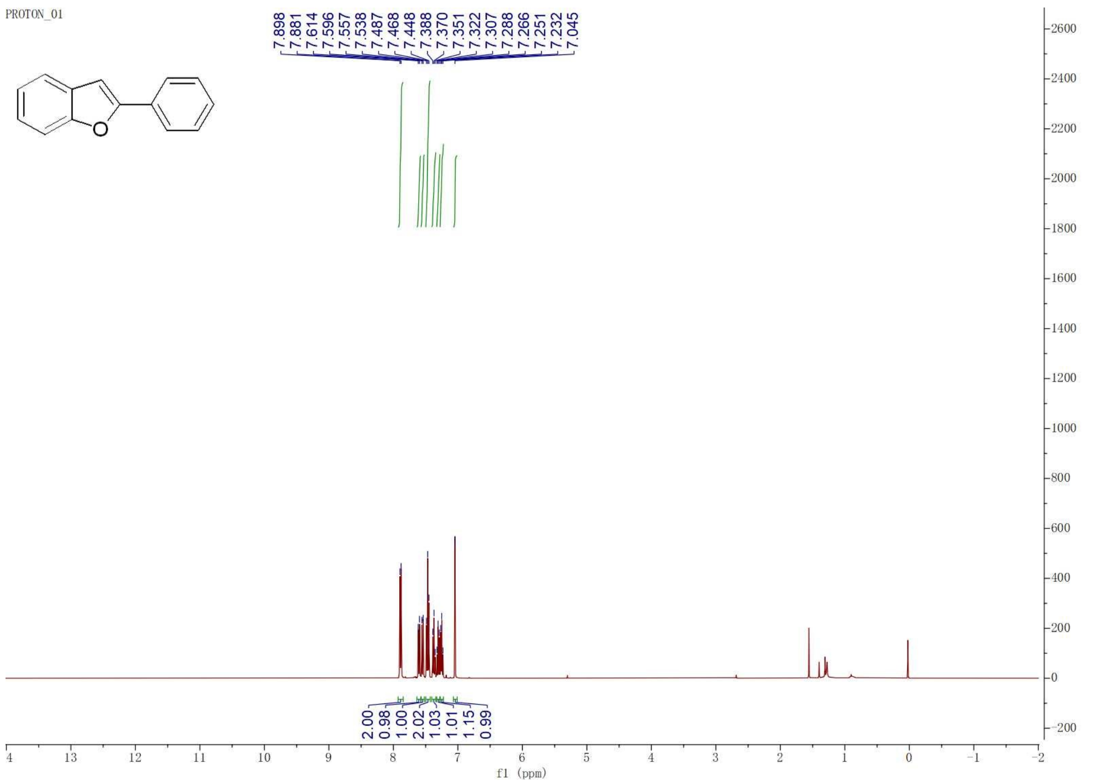

^{13}C NMR spectra of compound **5**:

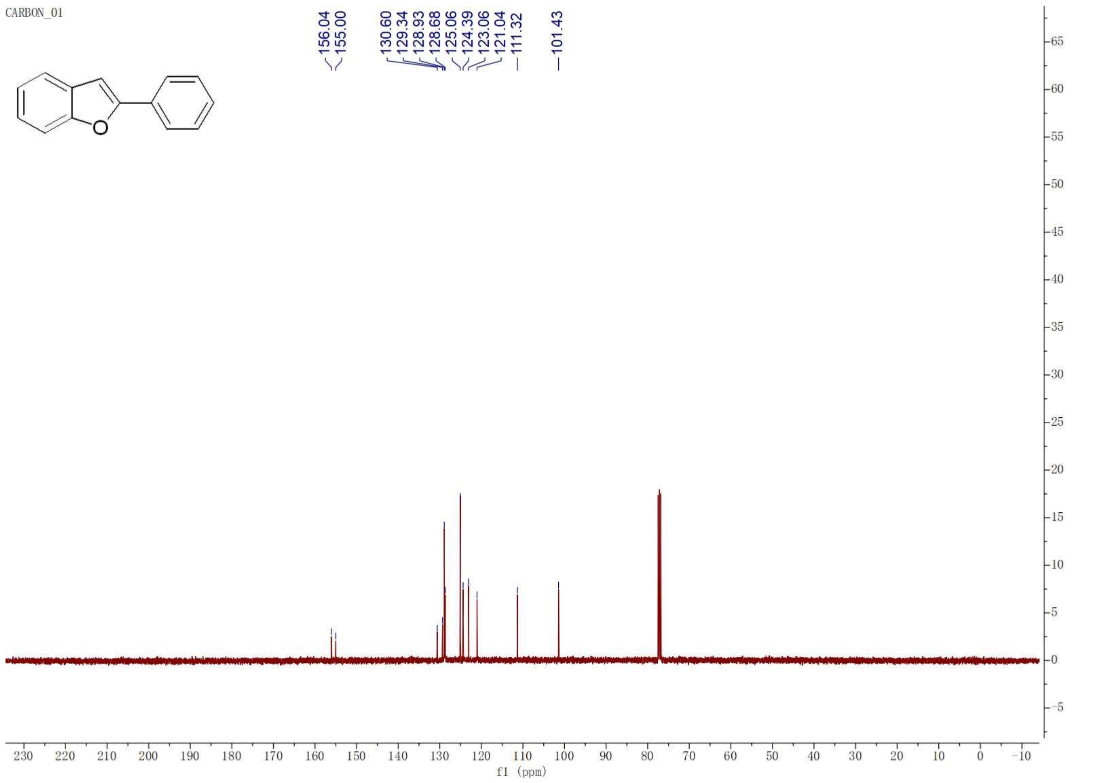

^{1}H NMR spectra of compound **6**:

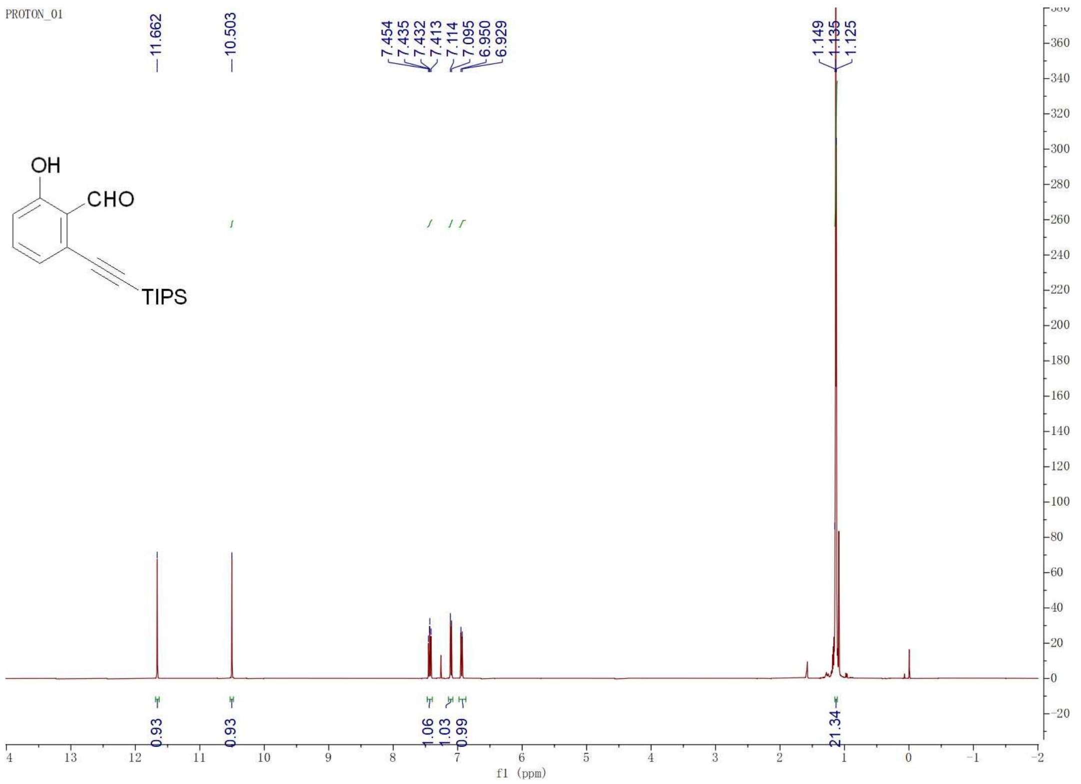

^{13}C NMR spectra of compound **6**:

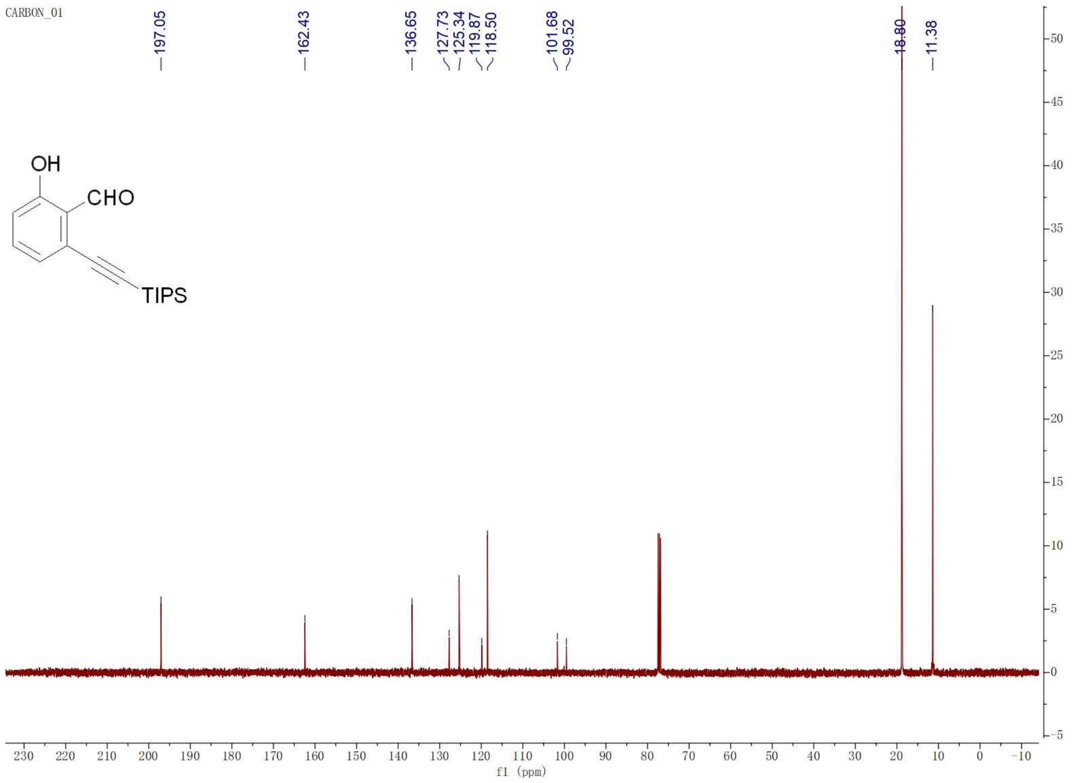

^{1}H NMR spectra of compound **7**:

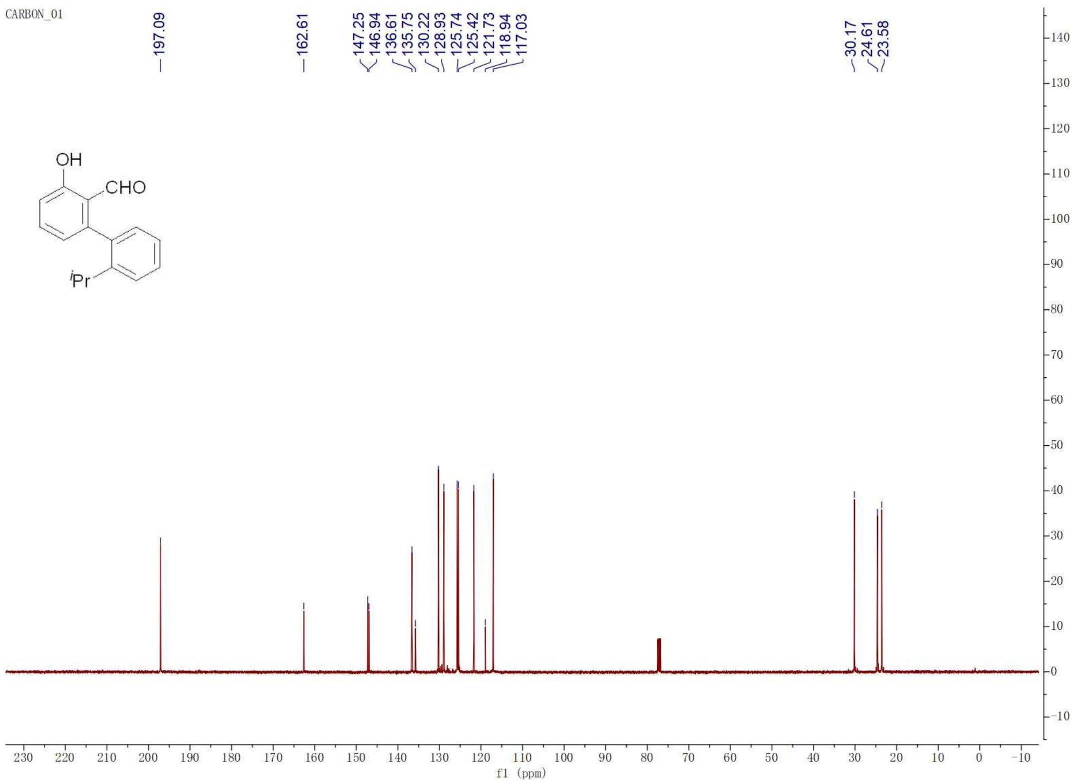

^{13}C NMR spectra of compound **7**:

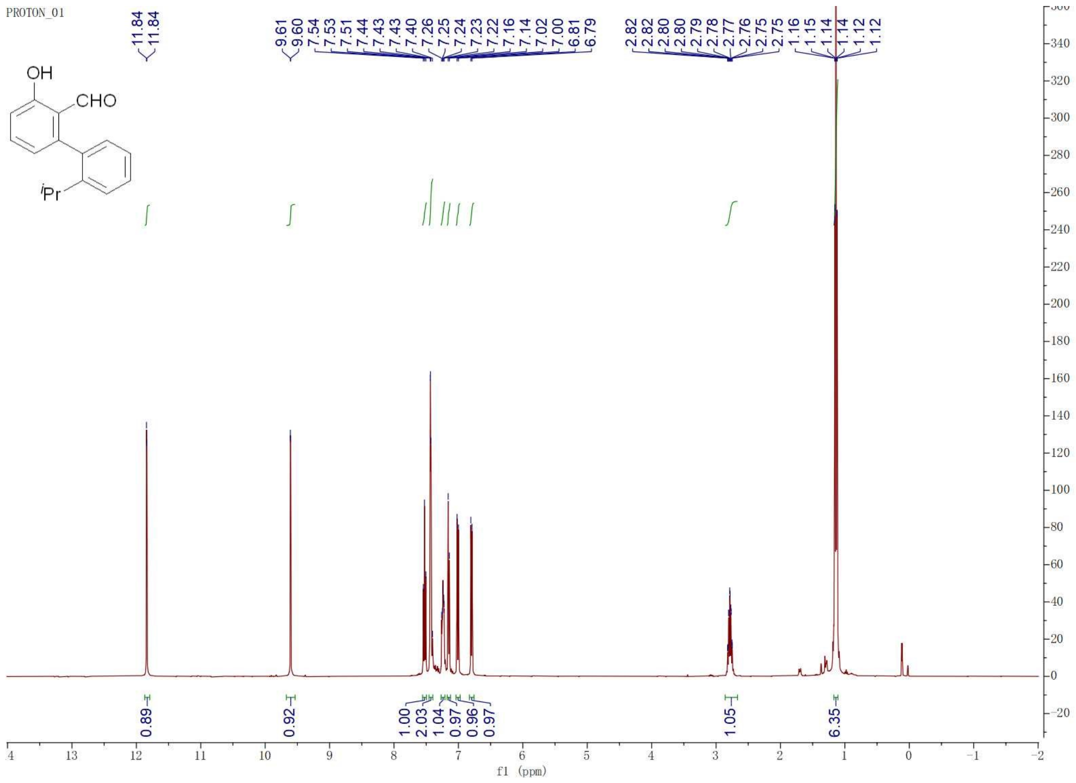

^{1}H NMR spectra of compound **8**:

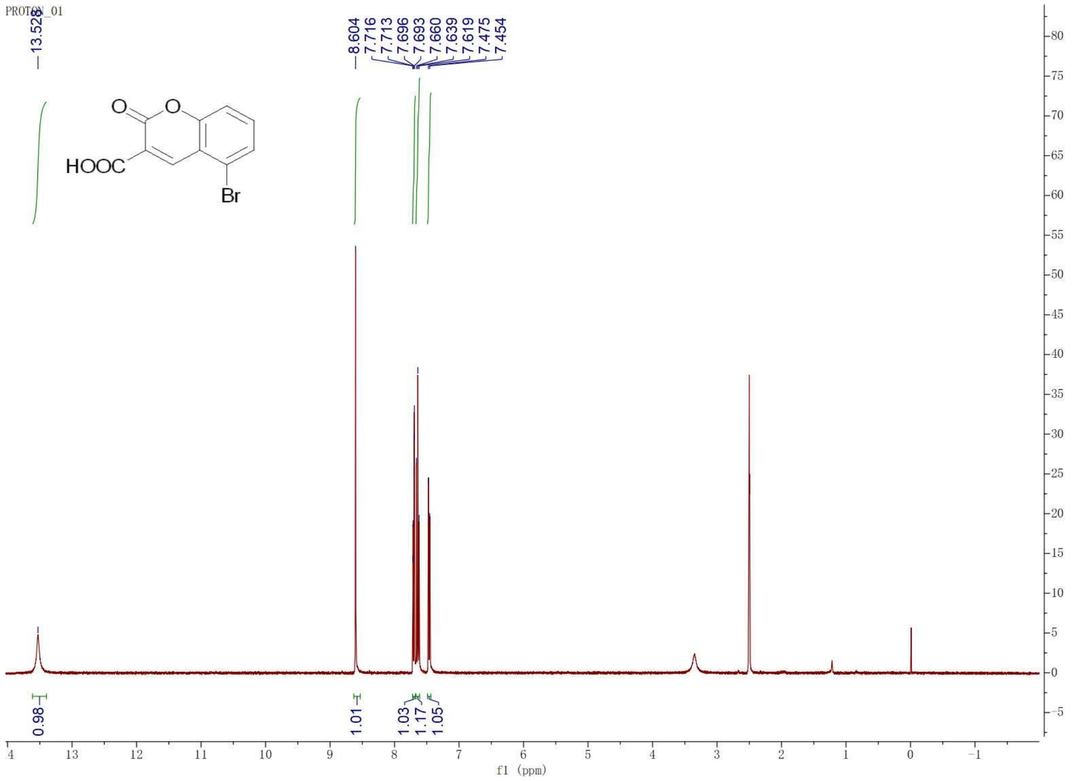

^{13}C NMR spectra of compound **8**:

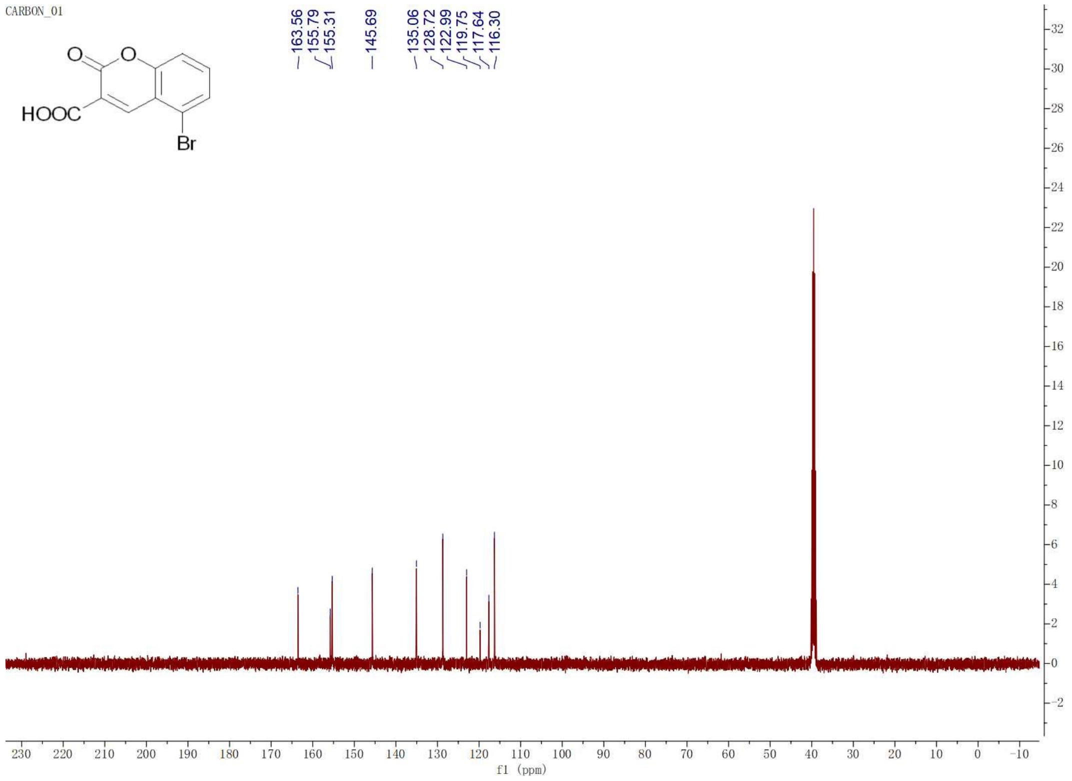

Supplement: RA-014-D4RA02994A-s001 [file RA-014-D4RA02994A-s001.pdf]
